# Novel Prenylated Indole Alkaloids with Neuroprotection on SH-SY5Y Cells against Oxidative Stress Targeting Keap1–Nrf2

**DOI:** 10.3390/md20030191

**Published:** 2022-03-04

**Authors:** Xueyang Xiao, Zhou Tong, Yuexing Zhang, Hui Zhou, Mengying Luo, Tianhui Hu, Ping Hu, Luqi Kong, Zeqin Liu, Chan Yu, Zhiyong Huang, Linzhen Hu

**Affiliations:** 1 National & Local Joint Engineering Research Centre of High-Throughput Drug Screening Technology, Hubei Key Laboratory of Biotechnology of Traditional Chinese Medicine, State Key Laboratory of Biocatalysis and Enzyme Engineering, School of Life Sciences, Hubei University, Wuhan 430062, China; xueyangxiao2021@163.com (X.X.); 202021107011179@stu.hubu.edu.cn (Z.T.); 201822110714021@stu.hubu.edu.cn (H.Z.); 201931107014028@stu.hubu.edu.cn (M.L.); 201931107014041@stu.hubu.edu.cn (T.H.); 13696476116@163.com (P.H.); 202121107012453@stu.hubu.edu.cn (L.K.); 202021107011053@stu.hubu.edu.cn (Z.L.); yuchan72@hubu.edu.cn (C.Y.); 2 College of Chemistry and Chemical Engineering, Hubei University, Wuhan 430062, China; zhangyuexing@sdu.edu.cn; 3 Tianjin Institute of Industrial Biotechnology, Chinese Academy of Sciences, Tianjin 300308, China

**Keywords:** prenylated indole alkaloids, neuroprotection, oxidative stress, Keap1–Nrf2

## Abstract

Oxidative stress has been implicated in the etiology of Parkinson’s disease (PD). Molecules non-covalently binding to the Keap1–Nrf2 complex could be a promising therapeutic approach for PD. Herein, two novel prenylated indole alkaloids asperpenazine (**1**), and asperpendoline (**2**) with a scarce skeleton of pyrimido[1,6-*a*]indole were discovered from the co-cultivated fungi of *Aspergillus ochraceus* MCCC 3A00521 and *Penicillium* sp. HUBU 0120. Compound **2** exhibited potential neuroprotective activity on SH-SY5Y cells against oxidative stress. Molecular mechanism research demonstrated that **2** inhibited Keap1 expression, resulting in the translocation of Nrf2 from the cytoplasm to the nucleus, activating the downstream genes expression of HO-1 and NQO1, leading to the reduction in reactive oxygen species (ROS) and the augment of glutathione. Molecular docking and dynamic simulation analyses manifested that **2** interacted with Keap1 (PDB ID: 1X2R) via forming typical hydrogen and hydrophobic bonds with residues and presented less fluctuation of RMSD and RMSF during a natural physiological condition.

## 1. Introduction

As one of the most commonplace neurodegenerative movement disorders, Parkinson’s disease (PD) affects more than 10 million individuals worldwide [1]. A wealth of evidence indicated that oxidative stress, characterized by the excessive production of reactive oxygen species, may be directly or indirectly implicated in the etiology of PD [2,3,4]. It is commonly identified that nuclear factor (erythroid-derived 2)-like 2 (Nrf2) regulates the expression level of intracellular ROS and glutathione (GSH), which also is a positive transcriptional regulation factor for modulating the expression of genes carrying with antioxidant response elements (ARE) [5]. Normally, Nrf2 binds with Kelch-like ECH-associated protein 1 (Keap1), which sequesters the former in the cytoplasm to promote its degradation via the ubiquitylation system, then leads to blocking the nuclear translocation of Nrf2. Disruption of the Keap1–Nrf2 protein–protein interaction (PPI) was beneficial to release Nrf2, which stimulated Nrf2 translocating from cytoplasm to nucleus, binding to ARE, and then activating the antioxidative defense system to generate antioxidative enzymes such as heme oxygenase 1 (HO-1) and NADPH quinone oxidoreductase 1 (NQO1) [4]. Although accumulating research developed Nrf2 activators involving covalently bonding with Keap1, the small-molecule non-covalently targeting of the Keap1–Nrf2 complex may offer better safety owing to improved efficacy and selectivity [6,7].

**Figure 1:**
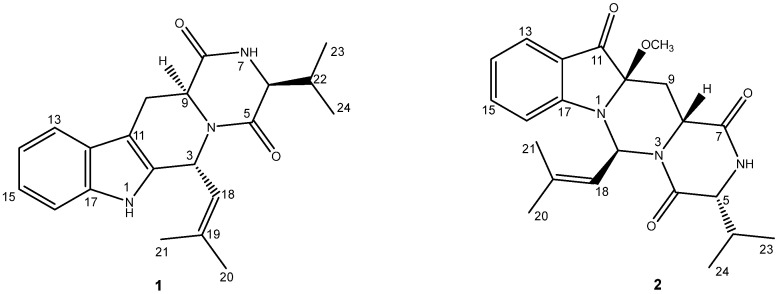
Structures of compounds **1** and **2**.

Small molecules metabolized from fungi possess versatile skeletons, whose fascinating bioactivities of treating human maladies are well documented and have been an original driving force for continuously exploring lead drugs [8,9,10]. Prenylated indole alkaloids were obtained mainly from diverse species of *Aspergillus* and *Penicillium* fungi [10,11,12], presenting neuroprotective effects on SH-SY5Y cells or BV-2 cells and primary microglial cells [13,14,15]. During our ongoing pharmaceutical investigation on exploring new neuroprotective lead compounds from fungi [14,16], two novel prenylated indole alkaloids with diketopiperazine motif, termed asperpenazine (**1**) and asperpendoline (**2**) (Figure 1), were discovered from an ethyl acetate extract of co-cultured *Aspergillus ochraceus* and *Penicillium* sp. HUBU 0120. Structurally, compound **2** is an unexpected prenylated diketopiperazine derivative possessing the rare skeleton of pyrimido[1,6-*a*]indole, resulting from the key steps of putative biosynthesis such as indole oxidation, pinacol rearrangement, regiospecific *N*-prenylation at the indole ring [17], and intramolecular oxidation. Metabolites were screened for neuroprotective effects on H_2_O_2_-injured SH-SY5Y cells, which demonstrated compound **2** predominantly protected cells from an oxidative stress injury. Further mechanism studies implied that **2** attenuated ROS accumulation, augmented GSH level, suppressed Keap1 protein and mRNA expressions, enhanced Nrf2 protein expression in the nucleus, and then upregulated HO-1 and NQO1 protein and their mRNA expressions. We delineated herein the isolation, structural determination, plausible biosynthetic pathway, the pharmacologically active evaluation, and molecular simulation, which is elucidated in what follows.

## 2. Results and Discussion

The marine-derived fungus *Aspergillus ochraceus* and soil-derived *Penicillium* sp. HUBU 0120 were co-inoculated on the potato dextrose agar (PDA) for 7 days and then cultivated in Erlenmeyer flasks (150 × 500 mL) containing sterilized rice at 25 °C for one month (Figure S1). The ethyl acetate (EtOAc) extract was chromatographed exhaustively to afford novel prenylated indole alkaloids trivially termed asperpenazine (**1**) and asperpendoline (**2**).

### 2.1. Chemical Structure Elucidation

**Figure 2:**
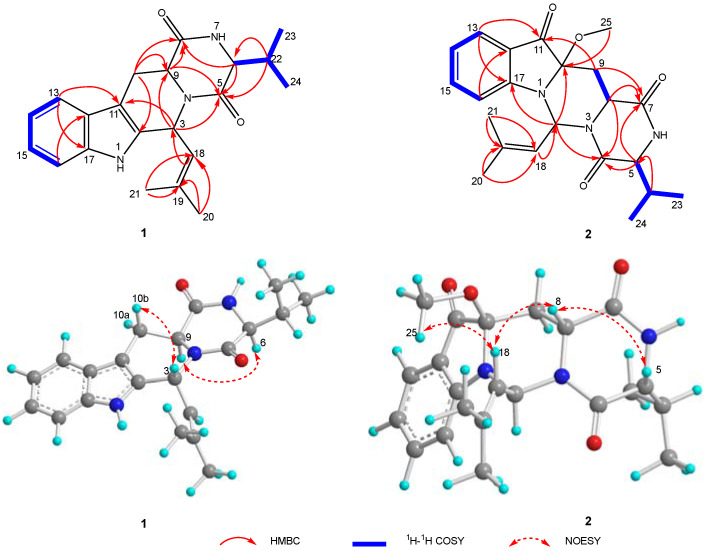
Key 2D correlations of **1** and **2**.

Asperpenazine (**1**), afforded as light-yellow needle crystals, whose molecular formula was deduced as C_21_H_25_N_3_O_2_ by the analysis of the high-resolution electrospray ionization mass spectroscopy (HRESIMS) with a quasimolecular ion at *m*/*z* 374.1903 ([M + Na]^+^ calcd. 374.1839). ^1^H and ^13^C nuclear magnetic resonance (NMR) spectra combining HSQC data analyses deciphered an *ortho*-disubstituted phenyl ring existence with the characteristic resonances [*δ*_C_ 126.7 (C-12), 118.4 (C-13), 120.1 (C-14), 122.5 (C-15), 111.2 (C-16), and 136.3 (C-17); *δ*_H_ 7.42 (d, *J* = 7.7 Hz, H-13), 7.06 (td, *J* = 7.6 and 1.1 Hz, H-14), 7.12 (td, *J* = 7.6 and 1.1 Hz, H-15), and 7.25 (d, *J* = 7.9 Hz, H-16)], four methyls [*δ*_C_ 26.2 (C-20), 19.1 (C-21), 19.0 (C-23), and 16.6 (C-24); *δ*_H_ 1.71 (d, *J* = 1.1 Hz, H-20), 1.98 (d, *J* = 1.1 Hz, H-21), 0.86 (d, *J* = 6.8 Hz, H-23), and 1.02 (d, *J* = 7.0 Hz, H-24)], one methylene [*δ*_C_ 28.2 (C-10); *δ*_H_ 3.46 (dd, *J* = 15.4 and 4.2 Hz, H-10a), and 2.87 (ddd, *J* = 15.3, 11.8, and 1.3 Hz, H-10b)], one sp^2^ methine [*δ*_C_ 120.7 (C-18); *δ*_H_ 5.27 (dt, *J* = 9.6 and 1.3 Hz, H-18)], four sp^3^ methines [*δ*_C_ 48.3 (C-3), 60.9 (C-6), 53.6 (C-9), and 32.7 (C-22); *δ*_H_ 6.45 (d, *J* = 9.5 Hz, H-3), 3.98 (brt, *J* = 2.4 Hz, H-6), 4.38 (dd, *J* = 11.7 and 4.0 Hz, H-9), and 2.38 (ddt, *J* = 10.5, 6.8, and 3.7 Hz, H-22)], two amide carbonyls [*δ*_C_ 163.8 (C-5) and 168.4 (C-8)], and three sp^2^ quaternary carbons [*δ*_C_ 132.6 (C-2), *δ*_C_ 106.6 (C-11), and *δ*_C_ 139.0 (C-19)]. Conjunction analysis of HSQC, ^1^H–^1^H COSY, and HMBC NMR data designated the ^1^H and ^13^C NMR signals of **1** (Table 1), which resulted in a proposal that **1** was likely to possess an indole diketopiperazine motif within [18,19]. ^1^H–^1^H COSY spin–spin systems of H-13/H-14/H-15/H-16, and HMBC correlations from H-13 to C-11/C-17 and from H-16 to C-12, together with the presence of a sp^2^ quaternary carbon [*δ*_C_ 132.6 (C-2)], roughly established an indole functional group (Figure 2). Further analyses of the HMBC data with the correlated resonances of H-6/C-5 and H-6/C-8, H-9/C-5 and H-9/C-8, and H-22/C-5 and H-22/C-6, along with the ^1^H–^1^H COSY coupling systems of H-6/H-22/H-23/H-24, constructed a diketopiperazine entity with an isopropyl group located at C-6. Finally, the HMBC spectrum with the correlated signals from H-3 to C-5, C-9, and C-11, and from H-10 to C-2, C-8, and C-9, connected the aforementioned motifs to the indole diketopiperazine skeleton with the C-3 isobutenyl substituent, forming an atypical fumitremorgin-type alkaloid [12], due to the HMBC correlations from Me-20 (and Me-21) to C-18 and C-19, and from H-18 to C-3, C-20, and C-21 (Figure 2).

**Figure 3:**
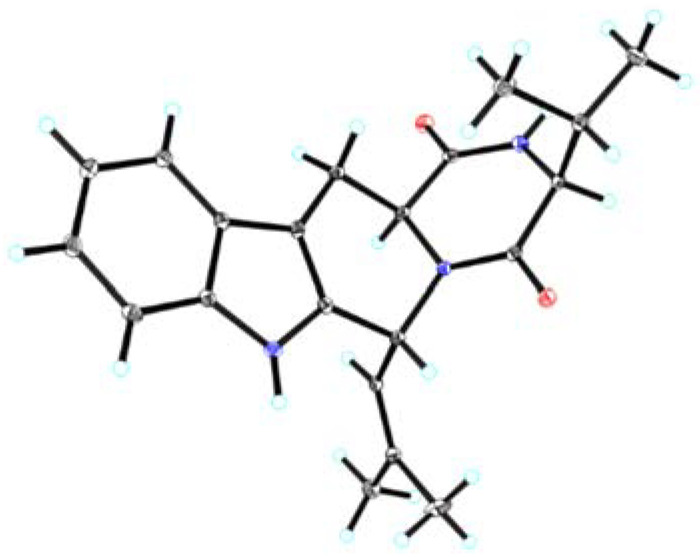
The ORTEP drawing structure of **1**.

The NOESY experiments were then performed to deduce the relative stereo-characteristics of **1** (Figure 2). Pivotal NOESY cross-peaks of H-6/H-9 and H-9/H-10a permitted an equatorial direction for these protons as an *α*-oriented assignment. Attributed to the key signal absence between H-3 and H-9, the signal of H-3/H-10b observed in the NOESY spectrum suggested the axial-directed for H-3 and H-10b as the *β* orientation. Completely, the absolute stereochemistry confirmation was finished via the single-crystal X-ray diffraction spectroscopy (XRD) on the single crystal of **1**. The chiral features of C-3, C-6, and C-9 were established as 3*R*,6*S*,9*S* (Figure 3), since the XRD data were collected with CuK*α* radiation, providing the Flack parameter of 0.08(4) (CCDC 2070312).

Asperpendoline (**2**) was isolated as a greenish-yellow powder with the molecular formula C_22_H_27_N_3_O_4_, possessing the eleven degrees of unsaturation, which was deduced by the HRESIMS positive ion peak at *m/z* 420.1897 ([M + Na]^+^ calcd. 420.1894). Interpretation of the ^1^H, ^13^C, and DEPT NMR spectra along with HSQC correlations provided the ^1^H and ^13^C chemical shift assignments of **2** (Table 1). The 1D and HSQC NMR spectra deduced the presence of 1,2-disubstituted phenyl ring with the typical resonances [*δ*_C_ 119.6 (C-12), 125.5 (C-13), 120.3 (C-14), 138.7 (C-15), 110.7 (C-16), and 157.7 (C-17); *δ*_H_ 7.59 (d, *J* = 8.0 Hz, H-13), 6.87 (t, *J* = 7.5 Hz, H-14), 7.55 (d, *J* = 7.7 Hz, H-15), and 7.17 (d, *J* = 8.4 Hz, H-16)], four methyls [*δ*_C_ 26.1 (C-20), 18.8 (C-21), 18.7 (C-23) and 16.0 (C-24); *δ*_H_ 1.79 (s, Me-20), 1.91 (s, Me-21), 0.88 (d, *J* = 7.1 Hz, Me-23), and 0.52 (d, *J* = 6.8 Hz, Me-24)], one methoxyl [*δ*_C_ 52.0 (C-25); *δ*_H_ 3.14 (s, Me-25)], one methylene [*δ*_C_ 36.4 (C-9); *δ*_H-9a_ 1.53 (t, *J* = 13.2 Hz) and *δ*_H-9b_ 2.64 (dd, *J* = 13.8 and 2.6 Hz)], four sp^3^ methines [*δ*_C_ 58.5 (C-2), 60.1 (C-5), 48.9 (C-8), and 32.2 (C-22); *δ*_H_ 7.23 (d, *J* = 9.8 Hz, H-2), 3.91 (brt, *J* = 2.4 Hz, H-5), 4.66 (dd, *J* = 13.0 and 2.3 Hz, H-8), and 2.29 (ddt, *J* = 13.7, 7.0, and 3.2 Hz, H-22)], one sp^2^ methine [*δ*_C_ 117.9 (C-18); *δ*_H_ 5.77 (d, *J* = 8.6 Hz, H-18)], one oxygenated carbon [*δ*_C_ 91.1 (C-10)], two amide carbonyls [*δ*_C_ 163.0 (C-4) and 166.6 (C-7)], one carbonyl [*δ*_C_ 198.4 (C-11)], and one sp^2^ quaternary carbon [*δ*_C_ 139.4 (C-19)]. Based on the ^1^H–^1^H COSY and HMBC NMR signals and the shift value analyses, the indolin-3-one unit presented in **2** rather than the indole motif as in **1**, was substantiated by the ^1^H–^1^H COSY coupling systems of H-13/H-14/H-15/H-16, the HMBC signals between H-13 and C-11/C-17, H-15 and C-17, and H-16 and C-12, and particularly, the presence of the C-10−N-1 bond, which reasonably supported the unusual downfield shift of C-10 (*δ*_C_ 91.1), although as an oxygenated carbon, C-10, with a methoxyl substituent resulting from the HMBC cross-peaks of Me-25/C-10 (Figure 2). Compound **2** has the identical diketopiperazine unit along with a C-6 isopropyl substituent to **1**, which is confirmed by the similar chemical shift values of C-4, C-5, C-7, C-8, C-22, C-23, and C-24, to those of **1**, and the HMBC correlated signals of H-5 to C-4 and C-7, H-8 to C-4 and C-7, and H-22 to C-5, as well as the ^1^H–^1^H COSY signals of H-5/H-22/H-23/H-24 observed in the spectrum. Furthermore, the key correlations in the HMBC spectrum of H-2 to C-4, C-10, and C-17, and H-9 to C-7, C-10, and C-11, as well as the COSY cross-peak of H-8/H-9, demonstrated the fusion of the indolin-3-one unit with the diketopiperazine motif, meeting the eleven indices of hydrogen deficiency, along with the isobutenyl functional group at C-2 for the observed signals of Me-20/21 to C-18 and C-19, and H-18 to C-2, C-20, and C-21 in the HMBC correlations (Figure 2). Architecturally, to the best of our knowledge, **2** with an unprecedented skeleton, which was forged via C-9–C-10–N-1–C-2 incorporating the indoline into the diketopiperazine motif, forming the scarce skeleton of pyrimido[1,6-*a*]indole, rather than via C-10–C-11=C-12–C-3 combination such as in that reported ones [18,19].

**Figure 4:**
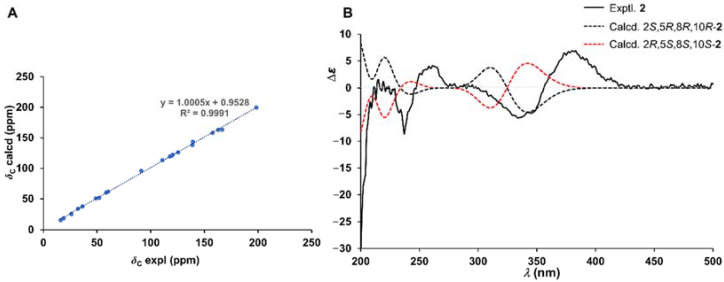
(**A**) Linear correlations between the experimental and calculated ^13^C NMR shifts of **2**; (**B**) the experimental and calculated ECD spectrum of **2**.

Detailed NOESY correlation analyses revealed the relative chiral features of **2**. The observed key signals in the NOESY spectrogram of H-5/H-8, H-8/H-18, and Me-25/H-18 suggested that these protons were co-facial, and then H-5, H-8, and the C−O bond were arbitrarily designated as the *α* orientation. As in the six-membered ring unit, the absence of NOESY cross-peaks of Me-25/H-2, Me-25/H-8, and H-2/H-8 illustrated the *β* orientation of H-2, which could be further supported due to the upfield shift of C-2 (*δ*_C_ 58.5) resulting from the *γ*-gauch effect. Therefore, the relative configuration as 2S*,5R*,8R*,10R*-**2** was constructed, and then the quantum chemical prediction on the ^13^C NMR shifts of which was executed via scaling methods [20,21] at the mPW1PW91/6-31G(d)-SCRF//M062X/6-31G(d) level. The calculated chemical shifts (*δ*) were obtained via the equation *δ* = (intercept − *σ*)/(−slope) (*σ* was the calculated isotropic value for a given nucleus; the values of the intercept and the slope were 193.2179 and −0.9537, respectively) [21], and then constructed the linear regression correlations between the calculated with the experimental ^13^C NMR shifts to acquire scaled calculated NMR shifts (Scal. Calc) (Table S1). The results with the high *R*^2^ value of 0.9991 (Figure 4A), the low average absolute deviation (AveDev) of 1.25, and the maximum absolute deviation (MaxDev) of 4.09 (Table S1), strongly supported the proposed relative configuration. Subsequently, the electronic circular dichroism (ECD) calculation was performed at the CAM-B3LYP-SCRF/def2-TZVPP//PBE0-SCRF/6-311G(d,p) level to simulate the Cotton effects of **2**, using the Gaussian 16 program. ECD simulation results matched well with the experimental curve (Figure 4B), which implied the absolute structure assignment 2*S*,5*R*,8*R*,10*R* of **2**.

**Figure 5:**
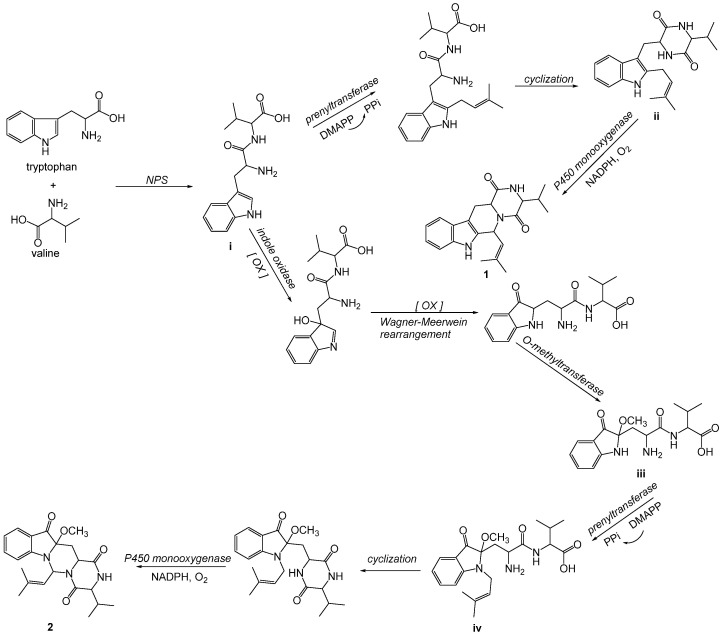
Plausible biosynthetic pathways for **1** and **2**.

Prenylated indole alkaloids, as well as derivatives thereof, whose skeletons are biogenetically originated from the condensation product involving tryptophan and other amino acids biocatalyzed by the nonribosomal peptide synthetase (NPS) [10,11,12]. The putative biosynthetic routes of compounds **1** and **2** are proposed in Figure 5. Firstly, tryptophan and valine occur condensation to form the key intermediate **i** via NPS catalyzation, which then proceeds along the following pathways. After bearing the prenylation via the dimethylallyl pyrophosphate (DMAPP) with the prenyltransferase [22], **i** successively undergoes cyclization to form intermediate **ii**, which then endures an intramolecular oxidative ring-closure reaction, a pivotal step involving C-3−N-4 bond formation mediated by the cytochrome P450 monooxygenase [23,24], and finally affording **1**. Alternatively, intermediate **i** occurs the oxidation attributable to indole oxidase catalyzation [10], Wagner–Meerwein rearrangement, and methoxylation involving *O*-methyltransferase due to structurally owning nucleophilic characteristics, to yield intermediate **iii**. Then, **iii** is regiospecifically *N*-prenylated by DMAPP at the indole ring [17], forming intermediate **iv**, which consecutively bears the aforementioned procedures, resulting in compound **2**.

### 2.2. Neuroprotection on SH-SY5Y Cells against Oxidative Stress

**Figure 6:**
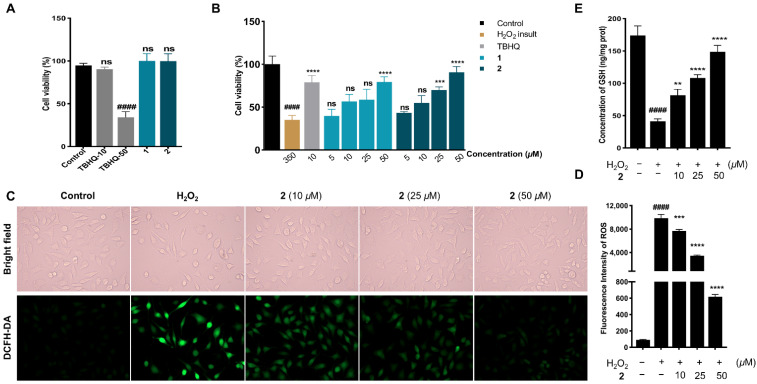
(**A**) Cell viabilities of **1** and **2** on SH-SY5Y cells with the concentration at 50 µM; (**B**) cytoprotective effects on H_2_O_2_ insult SH-SY5Y cells of TBHQ (10 µM), **1**, or **2** with the concentration ranging from 5–50 µM; (**C**) compound **2** attenuated ROS accumulation in H_2_O_2_ insult SH-SY5Y cells: the morphology changes and the fluorescent signals of the control (untreated), H_2_O_2_ insult, and H_2_O_2_ and **2** (10–50 µM) co-treated cells were captured by an inverted fluorescence microscope; (**D**) statistical analyses of DCFH–DA fluorescence intensity on the ROS production; (**E**) the concentration of GSH evaluation by ELISA. TBHQ was used as the positive control. H_2_O_2_ (350 µM) was adopted to insult SH-SY5Y cells. ^####^ *p* < 0.0001 vs. control group; ** p < 0.01, *** p < 0.001, and **** p < 0.0001 vs. H_2_O_2_ insult group; ns means no statistical significance. Statistical analyses were evaluated with two-way or one-way ANOVA; the values represent mean ± SD. All experiments were parallelly repeated three times in triplicate.

Compounds **1** and **2** contain carboline motifs incorporated into diketopiperazine units, both of which are considered favorable templates for the drug scaffolds design [25]. As human dopaminergic cells, SH-SY5Y cells have been typically applied on neuroprotection evaluation and molecular mechanism studies [26]. H_2_O_2_, a major ROS, is involved in neurodegenerative maladies including PD, which has been universally used to insult neuronal cells for the investigation of neuron injury under the oxidative stress condition [27,28]. The cytotoxicities of **1** and **2** on SH-SY5Y cells were firstly examined using the CCK-8 assay, and both compounds presented no cytotoxic activity with the concentration of 50 μM, whereas the positive control *tert*-butylhydroquinone (TBHQ) showed such activity (Figure 6A). The concentration of 50 μM thereby was adopted as the maximum one for isolates in the subsequent neuroprotection assessments. Then, the protection from H_2_O_2_ injury on cells of compounds was evaluated, and as shown in Figure 6B, both metabolites exhibited cytoprotective activity in dose-dependently on H_2_O_2_-induced cells, with **2** being more efficacious at the concentration of 50 μM than TBHQ at 10 μM. The overexpression of intracellular ROS plays a pivotal role in the proceeding of neuronal cells death via H_2_O_2_ insult [29]. DCFH–DA fluorescence staining and photography were carried out to assess the ROS level, and as shown in Figure 6C,D, with the concentration of 50 μM, **2** restored the morphology of H_2_O_2_-injured cells and attenuated the fluorescence intensity produced by ROS. As one of the most plentiful endogenous antioxidants, GSH has an essential effect on ROS detoxification and redox homeostasis regulation [30]. The level of GSH was determined using ELISA assay, and as Figure 6E shows, **2** promoted GSH concentration in a dose-dependent manner toward H_2_O_2_-induced cells.

**Figure 7:**
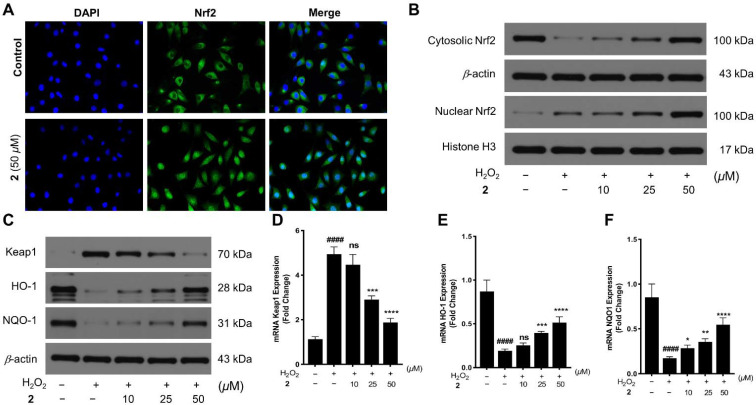
(**A**) Compound **2** promoted the Nrf2 nuclear translocation in SH-SY5Y cells. Cells were stained using DAPI and the Nrf2 antibody after being treated with **2** (50 μM) for 4 h. The represented signals were detected using the fluorescence microscope; (**B**) further, **2** (10–50 μM) promoted the protein expressions of Nrf2 in the cytoplasm and nucleus in H_2_O_2_ insult cells; (**C**) compound **2** enhanced the expressions of HO1 and NQO1 and inhibited the expression of Keap1. Protein expression levels were determined using immunoblot methods; (**D**−**F**) effects of **2** promoting the mRNA expression of Keap1, HO-1, and NQO1. Cells were treated using H_2_O_2_ (350 μM) or co-treated using H_2_O_2_ (350 μM) with **2** (10–50 μM) for 24 h. The relative levels of mRNA were measured by qRT−PCR analysis. In B−F, cells were cultivated using H_2_O_2_ (350 μM) or co-cultivated using H_2_O_2_ (350 μM) with **2** (10–50 μM) for 24 h. ^####^ *p* < 0.0001 vs. control (untreated cells) group; * p < 0.05, ** p < 0.01, *** p < 0.001, and **** p < 0.0001 vs. H_2_O_2_ insult group; ns means no statistical significance. Statistical analyses were evaluated with one-way ANOVA. The values represent mean ± SD. All experiments were parallelly repeated three times in triplicate.

The activation of antioxidative genes of Nrf2, such as HO-1 and NQO1, predominantly depends on the nuclear translocation of Nrf2 [31]. Based on the immunofluorescence assay, the effect of **2** toward Nrf2 translocation in cells from the cytosol to the nucleus was measured. The results showed that the **2**-treated group distinctly increased translocation of Nrf2 for the green fluorescence in the nucleus of cells (Figure 7A). Furthermore, Nrf2 expression in the cytosol, and the nucleus after cells treated with or without **2** was also detected via Western blotting. The level of cytosolic and nuclear Nrf2 protein exhibited the reverse trend in control and H_2_O_2_-insult groups, while consistent uptrend presented after H_2_O_2_-induced cells were administrated **2** with the concentrations ranging from 10–50 μM (Figure 7B), which may be attributable to disturbance of Keap1–Nrf2 PPI by **2**, releasing Nrf2 from ubiquitylation system. As phase-II detoxification enzymes, HO-1 and NQO1 are regulated by Nrf2, which is translocated into the nucleus and combines ARE to activate genes transcription of the formers. The further assessments of protein and mRNA expression on Keap1, HO-1, and NQO1 were measured using Western blotting and qRT–PCR assays, respectively. The protein expression levels of both HO-1 and NQO1 were enhanced, the ones of Keap1 were suppressed, along with the consistent expression trends of their mRNA levels, when H_2_O_2_-injured cells were treated with **2** (10–50 μM) (Figure 7C–F). Taken together, against H_2_O_2_-induced oxidative stress in SH-SY5Y cells, **2** inhibited expression of Keap1, leading to nuclear translocation of Nrf2, then inducing HO-1 and NQO1 expression via Nrf2 activation, which resulted in a reduced level of ROS and an augmented level of GSH, protecting cells from oxidative damage.

### 2.3. Molecular Docking and Dynamics Simulation of 2–1X2R

**Figure 8:**
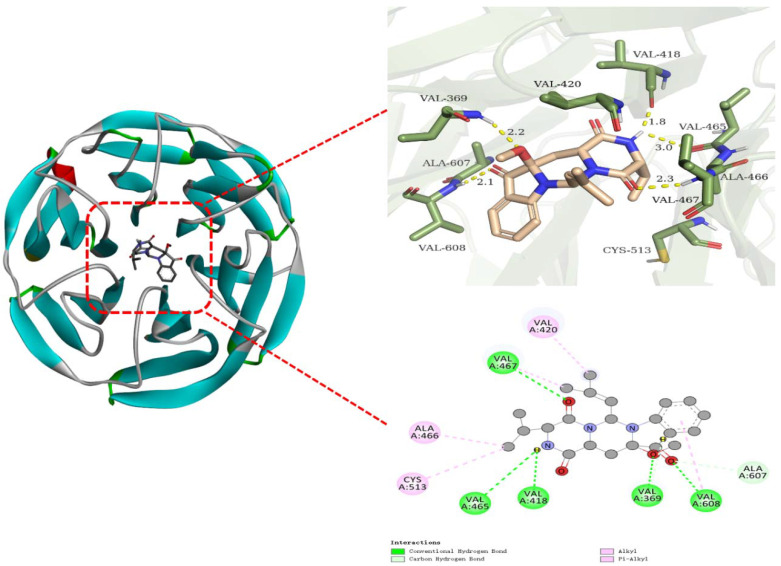
Visualization for molecular docking between **2** and 1X2R. **Left**: the whole perspective of the **2**–1X2R complex; **Right**: the amplified images of **2** docking in pocket sites of 1X2R (**upper**: 3D graphic; **lower**: 2D graphic).

The molecular docking approach is widely used to predict reliable binding dispositions between ligands and target proteins. As shown by the aforementioned results, **2** may target Keap1–Nrf2 PPI to modulate the Nrf2 signaling pathway, achieving neuroprotection toward SH-SY5Y cells from oxidative stress. Computational docking and molecular dynamics simulation, therefore, were carried out to predict the binding characteristics between **2** and Keap1 (PDB ID: 1X2R). The procedure of AutoDock 4.2.6 with MGLTools 1.5.6 (ADT) was performed for the virtual docking, which showed that **2** presented a high negative binding affinity (−8.46 kcal/mol), together with a low inhibition constant (K_i_) (632.06 nM) docking with 1X2R. The binding perspective of the **2**–1X2R complex was visualized via PyMOL Molecular Graphics System 2.4 and Discovery Studio 2020 (DS20). As shown in Figure 8, **2** bonded with 1X2R in a non-covalent manner, forming typical hydrogen bonds with amino acid residues of Val608, Val369, Val418, Val465, and Val467 along with the respective distance of 2.1, 2.2, 1.8, 3.0, and 2.3 Å, and hydrophobically interacting with residues of Cys513, Ala466, and Val420.

**Figure 9:**
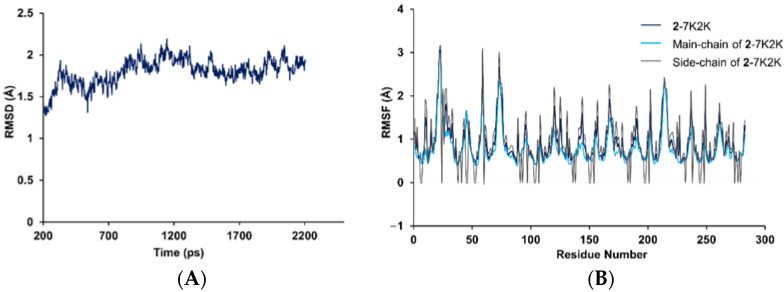
Molecular dynamics simulation analyses of RMSD and RMSF variations toward the **2**–1X2R complex under a natural physiological condition: (**A**) the RMSD of the conformational stabilities of the complex over time; (**B**) the RMSF, main-chain RMSF, and side-chain RMSF of the protein backbone atoms of the complex.

To examine the stability of the docked **2**–1X2R complex during physiological conditions, the molecular dynamics simulation (MDS) program of DS20 was used to calculate the root-mean-square deviation (RMSD) of the conformational stabilities over time and the root-mean-square fluctuation (RMSF) of the protein backbone atoms. After 4 ps CHARMm force field minimization and 200 ps of solvent equilibration, **2**–1X2R still exhibited thermal stability at 300 K from 204 ps to 2204 ps with the total energies from −59,517 to −59,919 kcal/mol. The average values of RMSD, RMSF, main-chain RMSF, and side-chain RMSF of the complex were detected as 1.70, 0.98, 0.83, and 1.01 Å, respectively (Figure 9), which illustrated the stabilization of the docked **2**–1X2R with less conformational fluctuations within a natural environment.

## 3. Materials and Methods

### 3.1. General Experimental Procedures

HRESIMS data were collected on a Bruker micro TOF II and SolariX 7.0 spectrometer (Bruker, Karlsruhe, Germany). Ultraviolet–visible (UV–Vis) absorption spectroscopy data were measured using a Bruker Vertex 70 spectrometer (Brucker Co., Karlsruhe, Germany). Infrared (IR) spectra were detected by a Fourier transform infrared spectrometer (Varian Cary 50 FT-IR, Varian Medical Systems, Salt Lake City, UT, USA). Optical rotation measurements were performed on a JASCO P-2200 digital polarimeter (JASCO, Tokyo, Japan) at 20 °C. ECD spectra were determined by a JASCO J-810 spectrometer (JASCO, Tokyo, Japan). The Bruker AM-400 spectrometer (Brucker Co., Karlsruhe, Germany) was performed to collect ^1^H NMR (400 MHz) and ^13^C NMR (100 MHz) data of compounds, whose chemical shifts were obtained in ppm via referring to the solvent peaks (CDCl_3_, *δ*_H_ 7.24 and *δ*_C_ 77.23). XRD data were recorded by a Bruker APEX DUO diffractometer (Brucker Co., Karlsruhe, Germany) with graphite-monochromated CuK*α* radiation. Silica gel (200–300 mesh) and reversed-phase C_18_ (RP-C_18_, spherical, 20 μM) were purchased from Santai Technologies, Inc., Suzhou, China. Sephadex LH-20 was afforded by Beijing Solarbio Science and Technology Co., Ltd., China. Thin-layer chromatography (TLC) was fulfilled using silica gel 60 F_254_ (GF_254_) (Qingdao Haiyang Chemical Co., Ltd., Qingdao, China). The semi-preparative high-performance liquid chromatography (HPLC) instrument (Waters 600, Milford, MA, USA) was performed to repurify compounds **1** and **2** over a Shim-Pack GIST-C_18_ column (5 μM, 10 × 250 mm, Shimadzu (Shanghai, China) Global Laboratory Consumables Co., Ltd., Shanghai, China).

### 3.2. Strain Material

*Aspergillus ochraceus* MCCC 3A00521, derived from the deep-sea water in the Pacific Ocean, the voucher specimens of which were provided by Marine Culture Collection of China. *Penicillium* sp. HUBU 0120, collected from the soil of Xishan Mountain, Kunming, Yunnan Province, China in May 2018, the identification of which was accomplished referring to the morphological features, and the sequence analyses of the internal transcribed spacer (ITS) region of the ribosomal RNA (rDNA) using ITS 1 and ITS 4, and the sequence data of which were submitted to GenBank with the accession number MW463395. Based on the BLAST consequences of ITS genes in NCBI, the phylogenetic tree of *Penicillium* sp. HUBU 0120 was constructed via MEGA 7.0 software using the neighbor-joining (N-J) method (Figure S2). The inoculated fungus *A. ochraceus* MCCC 3A00521 was deposited in the Strain Preservation Center, School of Life Sciences, Hubei University, China. The fungus *P.* sp. HUBU 0120 was preserved in China Center for Type Culture Collection, Wuhan University, China (preservation ID: CCTCC M2021412).

### 3.3. Fermentation, Extraction and Isolation

The co-cultured fungi of *A. ochraceus* MCCC 3A00521 and *P.* sp. HUBU 0120 were inoculated in PDA culture plates at 25 °C for a week. The agar containing two fungi was divided into small pieces and then subjected to sterilized Erlenmeyer flasks (150 × 500 mL), which were pre-added with 100 g rice, 150 mL H_2_O, 0.8% NaCl, 0.5% KCl, and 0.8% MgSO_4_, fermenting at 25 °C for 30 days. Then, the growing fungi were sequestered by adding 150 mL EtOAc to each flask. The fermentation was extracted six times using EtOAc (6 × 10 L) and then yielded a crude extract (400 g) under the vacuum evaporation. Subsequently, the extract was subjected to a silica gel column (silica gel 4.0 kg, column 20 × 150 cm), eluting with petroleum ether, methylene chloride (CH_2_Cl_2_), and EtOAc, in turn. The methylene chloride partition (100 g) was fractionated into seven fractions (Fr1–Fr7) by column chromatography (CC) with silica gel (2.0 kg, 15 × 100 cm), eluting with CH_2_Cl_2_−CH_3_OH (300:1 → 10:1). After being detected with TLC, the fraction Fr4 (10 g) was selected and partitioned into six subfractions of Fr4.1–Fr4.6 by the gradient elution on the medium pressure liquid chromatography (MPLC, RP-C_18_, 5 × 50 cm) with MeOH−H_2_O (10:90 → 100:0). The subfraction Fr4.4 (2 g) was subjected to the Sephadex LH-20 CC (4 × 185 cm, MeOH) to obtain five subparts (Fr4.4.1–Fr4.4.5). The subpart Fr4.4.2 (400 mg) was obtained after removing the solvent using vacuum evaporation. Finally, Fr4.4.2 was further repurified by HPLC (MeOH–H_2_O, *v/v* 45:55, 2.0 mL/min, 254 nm) over a semi-preparative Shim-Pack GIST-C_18_ column to yield **1** (15 mg; retention time: 35 min) and **2** (5 mg; retention time: 55 min).

**Asperpenazine** (**1**): light-yellow needle crystals; ${\left[\alpha \right]}_{D}^{20}$ −283.4 (*c* 0.53, CH_3_OH); UV (CH_3_OH) *λ*_max_ (log *ε*) 227 (4.77) and 272 (4.03) nm; IR (KBr) *ν*_max_ 3279, 3059, 2963, 1679, 1450, and 1327 cm^–1^; ECD *λ*_max_ (∆*ε*) 216 (−12.16) and 269 (−3.62) nm (the experimental ECD spectrum was shown in Figure S3); ^1^H and ^13^C NMR data, see Table 1; HRESIMS: *m/z* 374.1903 [M + Na]^+^ (calcd for C_21_H_25_N_3_O_2_Na, 374.1839). HRESIMS, UV, IR, and NMR spectra of **1** were shown in Figures S4–S13.

**Asperpendoline** (**2**): greenish-yellow powder; ${\left[\alpha \right]}_{D}^{20}$ +96.8 (*c* 0.24, MeOH); UV (CH_3_OH) *λ*_max_ (log *ε*) 238 (4.61) and 399 (3.73) nm; IR (KBr) *ν*_max_ 3215, 2926, 1722, 1685, 1583, 1466, 1441, and 1319 cm^–1^; ECD *λ*_max_ (∆*ε*) 237 (−8.63), 261 (+4.09), 334 (−5.63), and 382 (+6.90) nm; ^1^H and ^13^C NMR data, see Table 1; HRESIMS: *m/z* 420.1897 [M + Na]^+^ (calcd for C_22_H_27_N_3_O_4_Na, 420.1894). HRESIMS, UV, IR, and NMR spectra of **2** were shown in Figures S14–S23.

### 3.4. Single-Crystal X-ray Data for Asperpenazine (1)

C_21_H_25_N_3_O_2_•H_2_O, *M* = 369.45, *a* = 8.9067(2) Å, *b* = 13.3081(3) Å, *c* = 15.9542(3) Å, *α* = 90°, *β* = 90°, *γ* = 90°, *V* = 1891.07(7) Å^3^, *T* = 100.(2) K, space group *P*212121, *Z* = 4, *μ*(Cu Kα) = 0.706 mm^−1^, 20,318 reflections measured, 3708 independent reflections (*R_int_* = 0.0294). The final *R_1_* values were 0.0283 (*I* > 2*σ*(*I*)). The final *wR*(*F*^2^) values were 0.0722 (*I* > 2*σ*(*I*)). The final *R_1_* values were 0.0284 (all data). The final *wR*(*F*^2^) values were 0.0723 (all data). The goodness of fit on *F*^2^ was 1.100. Flack parameter = 0.08(4).

### 3.5. Cytotoxicity and Cytoprotection Evaluation

The SH-SY5Y cell line was kindly provided by the Institute of Materia Medica, the Chinese Academy of Medical Sciences, and Peking Union Medical College. The cytotoxicity and cytoprotection of compounds on SH-SY5Y cells treated with or without H_2_O_2_ were determined using a CCK-8 assay. The detailed experiments were described in previous studies [14,16]. The CCK-8 kit was purchased from Beyotime Biotechnology Co., Ltd., Shanghai, China. Briefly, inoculated cells in 96-well plates were treated without or with H_2_O_2_ (350 μM) or being co-treated with H_2_O_2_ (350 μM) and compounds with designated concentrations in the incubator under 5% CO_2_ at 37 °C for 24 h. As cells grew to 75% confluence, the CCK-8 solution (10 μL) was then added and cultivated for 2 h. The envision 2104 multilabel reader (PerkinElmer, Waltham, MA, USA) was used to measure the optical density (OD) at 450 nm of each well. The cell viabilities were evaluated via the formula:

Cell viability% = [OD_(experimental group)_ − OD_(blank)_]/[OD_(control group)_ − OD_(blank)_] × 100% (means ± SD, *n* = 3).

### 3.6. ROS Level Evaluation

The intracellular ROS level of cells was determined by the ROS assay kit (Beyotime Biotechnology Co., Ltd., Shanghai, China) with the DCFH–DA as the probe [32], according to the protocol afforded by the manufacturer. After being treated without or with H_2_O_2_ (350 μM), or co-treated with H_2_O_2_ (350 μM) and **2** (10, 25, and 50 μM) for 24 h, cells were washed and then stained with a diluted DCFH–DA solution in dark for 20 min. A fluorescence microscope was used to observe and photograph cells. The value of integrated OD for each group was recorded for the expression of the fluorescence intensity.

### 3.7. GSH Level Evaluation

The GSH produced in cells was assessed through the GSH ELISA assay kit (ELK Biotechnology Co., Ltd., Wuhan, China), following the protocol provided by the manufacturer. The procedures in this assay were executed as previously described [16].

### 3.8. Nuclear Translocation of Nrf2

The microscopy immunofluorescence staining method was used in the Nrf2 translocation assay. Firstly, cells were cultured with or without **2** (50 μM) for 24 h, fixed with paraformaldehyde (4%) for 20 min. Then, cells were successively permeabilized using 0.1% Triton X-100, washed by PBS, and blocked with BSA (bovine serum albumin, 5%). After being treated with the primary antibody Nrf2 (Wuhan Sanying Biotechnology Co., Ltd., Wuhan, China) and secondary antibody (Wuhan Sanying Biotechnology Co., Ltd., Wuhan, China), cells were lastly stained by DAPI (Beyotime Biotechnology Co., Ltd., Shanghai, China). The photographs of cells were captured under the fluorescence microscope.

### 3.9. Western Blotting

The procedures of cells cultivation and treatment were identical to the above mentioned. The radioimmunoprecipitation (RIPA) assay was carried out to yield the lysates of cells, which were then centrifugated at 12,000 rpm for 5 min to obtain the supernatant for immunoblot analyses. The total protein concentrations were measured by the BCA kit (Aspen Biotechnology Co., Ltd., Wuhan, China) according to the instruction provided by the manufacturer. The procedures of the electrophoresis and Western blotting analyses referenced the reported literature [33].

### 3.10. Quantitative Real-Time Reverse Transcriptase—Polymerase Chain Reaction (qRT−PCR)

Cells treated without or with H_2_O_2_ (350 μM) alone or co-treated with H_2_O_2_ (350 μM) and **2** with doses ranging from 10–50 μM for 24 h and then harvested in TRIpure total RNA extraction reagent (ELK Biotechnology Co., Ltd., Wuhan, China). The cDNA was probed after reverse transcription via the EntiLink™ Reverse Transcriptase kit (ELK Biotechnology Co., Ltd., Wuhan, China) with the oligo(dT)_12–18_ primers. The primers in this research were synthesized by Wuhan Jin-Kai-Rui Biological Engineering Co., Ltd., Wuhan, China. The primer information is shown in Table S2. According to the protocol of the EnTurbo^TM^ SYBR Green PCR SuperMix kit (ELK Biotechnology Co., Ltd., Wuhan, China), the qRT−PCR experiments were performed on a StepOne^TM^ Real-Time PCR detection system (Life Technologies Corp., Carlsbad, CA, USA).

### 3.11. Molecular Docking

The molecular docking study on the binding dispositions between **2** and Keap1 (PDB ID: 1X2R) was performed using AutoDock 4.2.6 with MGLTools 1.5.6 (ADT). The details of the docking procedures were delineated in our previous research [14]. The coordinates of grid box size were determined via the AutoGrid program and designated at 126 × 126 × 126 (x, y, and z) points, centered at x, y, and z dimensions of −23.558 × −4.445 × 12.356, as well as the grid spacing set at 0.375 Å. The docking between **2** and 1X2R was executed using the default parameters of the ADT program.

### 3.12. Molecular Dynamics Simulation

The approach of the MDS on **2**–1X2R was shown in detail in a reported study [14]. The standard dynamics module in DS20 was carried out. The **2**–1X2R complex was assigned a CHARMm force field. Then, the solvation module for the complex was performed using the default parameters to simulate a natural physiological environment. Finally, the standard dynamics cascade program was performed under an equilibration time of 200 ps and a production time of 2000 ps with 32 processors. Other parameters were set as default values of the program.

## 4. Conclusions

In the present study, two novel prenylated indole alkaloids asperpenazine (**1**) and asperpendoline (**2**), with diketopiperazine motifs, were discovered from the co-cultivated fungi of *A. ochraceus* MCCC 3A00521 and *P.* sp. HUBU 0120. In particular, **2** possessed an unprecedented skeleton, incorporating the indoline into the diketopiperazine motif to forge a scarce skeleton of pyrimido[1,6-*a*]indole. The plausible biogenetic pathway suggested that the indole oxidase catalyzation, Wagner–Meerwein rearrangement, methoxylation, and regiospecific *N*-prenylation should be involved. Furthermore, compound **2** exhibited promising neuroprotective effects on SH-SY5Y cells from oxidative damage, which may be attributable to **2** non-covalently binding with Keap1, resulting in the nuclear translocation of Nrf2 to activate the expression of HO-1 and NQO1, then attenuating the ROS accumulation and enhancing the GSH level. Computational molecular docking and dynamic simulation analyses demonstrated that **2** formed typical hydrogen and hydrophobic bonds with residues of Keap1, presenting less fluctuation of RMSD and RMSF during a general physiological circumstance. Thus, compound **2** will shed light on the skeleton design of novel neuroprotective drugs non-covalently bonding with Keap1–Nrf2.

## Figures and Tables

**Table 1:** ^1^H (400 MHz) and ^13^C (100 MHz) NMR data of compounds **1** and **2** (recorded in CDCl_3_).

No.	1		2	
	*δ*_H_	*δ*_C_	*δ*_H_	*δ*_C_
2		132.6	7.23 d (9.8)	58.5
3	6.45 d (9.5)	48.3		
4				163.0
5		163.8	3.91 brt (2.4)	60.1
6	3.98 brt (2.4)	60.9		
7				166.6
8		168.4	4.66 dd (13.0, 2.3)	48.9
9	4.38 dd (11.7, 4.0)	53.6	Ha 1.53 t (13.2) Hb 2.64 dd (13.8, 2.6)	36.4
10	Ha 3.46 dd (15.4, 4.2) Hb 2.87 ddd (15.3, 11.8, 11.3)	28.2		91.1
11		106.6		198.4
12		126.7		119.6
13	7.42 d (7.7)	118.4	7.59 d (8.0)	125.5
14	7.06 td (7.6, 1.1)	120.1	6.87 t (7.5)	120.3
15	7.12 td (7.6, 1.1)	122.5	7.55 d (7.7)	138.7
16	7.25 d (7.9)	111.2	7.17 d (8.4)	110.7
17		136.3		157.7
18	5.27 dt (9.6, 1.3)	120.7	5.77 d (8.6)	117.9
19		139.0		139.4
20	1.71 d (1.1)	26.2	1.79 s	26.1
21	1.98 d (1.1)	19.1	1.91 s	18.8
22	2.38 ddt (10.5, 6.8, 3.7)	32.7	2.29 ddt (13.7, 7.0, 3.2)	32.2
23	0.86 d (6.8)	19.0	0.88 d (7.1)	18.7
24	1.02 d (7.0)	16.6	0.52 d (6.8)	16.0
25-OCH_3_			3.14 s	52.0

## Data Availability

Not applicable.

## Supplementary Materials

The following are available online at https://www.mdpi.com/article/10.3390/md20030191/s1, Figure S1: The co-cultured and the fermentation of fungi, Figure S2: *Penicillium* sp. HUBU 0120 and the phylogenetic tree of ITS genes, computational details of **2**, Figure S3: The experimental ECD spectrum of **1**, Table S1: Deviations between the calculated and the experimental ^13^C NMR chemical shifts for **2**, Table S2: Primer information of Keap1, HO-1, NQO-1, Histone H3, and *β*-Actin, Figures S4–S23: HRESIMS, UV, IR, ^1^H NMR, ^13^C NMR, DEPT 135, HSQC, HMBC, ^1^H–^1^H COSY, and NOESY spectra of compounds **1** and **2**, and X-ray crystallographic data of **1** in CIF format were also included. References [34,35,36] are cited in the supplementary materials.

## Abbreviations

DEPT	Distortionless enhancement by polarization transfer
HSQC	^1^H detected heteronuclear single quantum coherence spectroscopy
HMBC	^1^H detected heteronuclear multiple bond connectivity spectroscopy
^1^H–^1^H COSY	^1^H–^1^H chemical shift correlated spectroscopy
NOESY	Nuclear overhauser effect spectroscopy
ORTEP	Oak ridge thermal ellipsoid plot
CCK-8	Cell counting kit-8
DCFH–DA	2,7-Dichlorodihydrofluorescein diacetate
ELISA	Enzyme-linked immunosorbent assay
DAPI	4′,6-Diamidino-2-phenylindole
