# Supplementary material for: Novel Prenylated Indole Alkaloids with Neuroprotection on SH-SY5Y Cells against Oxidative Stress Targeting Keap1–Nrf2
Source: Mar Drugs. 2022 Mar 4;20(3):191. doi: 10.3390/md20030191 (PMC8952805; doi:10.3390/md20030191)

Novel Prenylated Indole Alkaloids with Neuroprotection on SH-SY5Y Cells against Oxidative Stress Targeting Keap1–Nrf2

Xueyang Xiao [1,†], Zhou Tong [1,†], Yuexing Zhang [2,†], Hui Zhou [1], Mengying Luo [1], Tianhui Hu [1], Ping Hu [1], Luqi Kong [1], Zeqin Liu [1], Chan Yu [1], Zhiyong Huang [3,*], and Linzhen Hu [1,*]

[1] National & Local Joint Engineering Research Centre of High-Throughput Drug Screening Technology, Hubei Key Laboratory of Biotechnology of Traditional Chinese Medicine, State Key Laboratory of Biocatalysis and Enzyme Engineering, School of Life Sciences, Hubei University, Wuhan 430062, Hubei Province, China; xueyangxiao2021@163.com (X.X.); 202021107011179@stu.hubu.edu.cn (Z.T.); 201822110714021@stu.hubu.edu.cn (H.Z.); 201931107014028@stu.hubu.edu.cn (M.L.); 201931107014041@stu.hubu.edu.cn (T.H.); 13696476116@163.com (P.H.); 202121107012453@stu.hubu.edu.cn (L.K.); 202021107011053@stu.hubu.edu.cn (Z.L.); yuch-an72@hubu.edu.cn (C.Y.)

[2] College of Chemistry and Chemical Engineering, Hubei University, Wuhan 430062, Hubei Province, China; zhangyuexing@sdu.edu.cn

[3] Tianjin Institute of Industrial Biotechnology, Chinese Academy of Sciences, Tianjin 300308, China

[†] These authors contributed equally to this work.

[*] Authors to whom correspondence should be addressed; E-mails: huang_zy@tib.cas.cn (Z. Y. H), Tel.: +86-22-84861931 (Z. Y. H.); linzhenhu@hubu.edu.cn (L. Z. H.), Tel.: +86-27-88661237-8023 (L. Z. H.)

Contents

Figure S1. The Coculture and the fermentation of fungi. 3
Figure S2. *Penicillium* sp. HUBU 0120 and the phylogenetic tree of ITS genes. 3
Figure S3. The experimental ECD spectrum of **1**. 3
Table S1. Deviations between the calculated and the experimental ^{13}C NMR chemical shifts for **2**. 4
Table S2. Primer information of Keap1, HO-1, NQO-1, and *β*-Actin in the present study. 4
Quantum-chemical computation for ECD 5
Quantum-chemical computation for ^{13}C NMR 35
Figure S4. HRESIMS of compound **1** 37
Figure S5. UV of compound **1** (in MeOH) 38
Figure S6. IR of compound **1** (KBr disc) 38
Figure S7. ^{1}H NMR spectrum of compound **1** (in $CDCl_3$) 39
Figure S8. ^{13}C NMR spectra of compound **1** (in $CDCl_3$) 40
Figure S9. DEPT135 spectrum of compound **1** (in $CDCl_3$) 41
Figure S10. HSQC spectrum of compound **1** (in $CDCl_3$) 42
Figure S11. HMBC spectrum of compound **1** (in $CDCl_3$) 43
Figure S12. ^{1}H–^{1}H COSY spectrum of compound **1** (in $CDCl_3$) 44
Figure S13. NOESY spectrum of compound **1** (in $CDCl_3$) 45
Figure S14. HRESIMS of compound **2** 46
Figure S15. UV of compound **2** (in MeOH) 46
Figure S16. IR of compound **2** (KBr disc) 46
Figure S17. ^{1}H NMR spectrum of compound **2** (in $CDCl_3$) 47
Figure S18. ^{13}C NMR spectrum of compound **2** (in $CDCl_3$) 48
Figure S19. DEPT135 spectrum of compound **2** (in $CDCl_3$) 49
Figure S20. HSQC spectrum of compound **2** (in $CDCl_3$) 50
Figure S21. HMBC spectrum of compound **2** (in $CDCl_3$) 51
Figure S22. ^{1}H–^{1}H COSY spectrum of compound **2** (in $CDCl_3$) 52
Figure S23. NOESY spectrum of compound **2** (in $CDCl_3$) 53

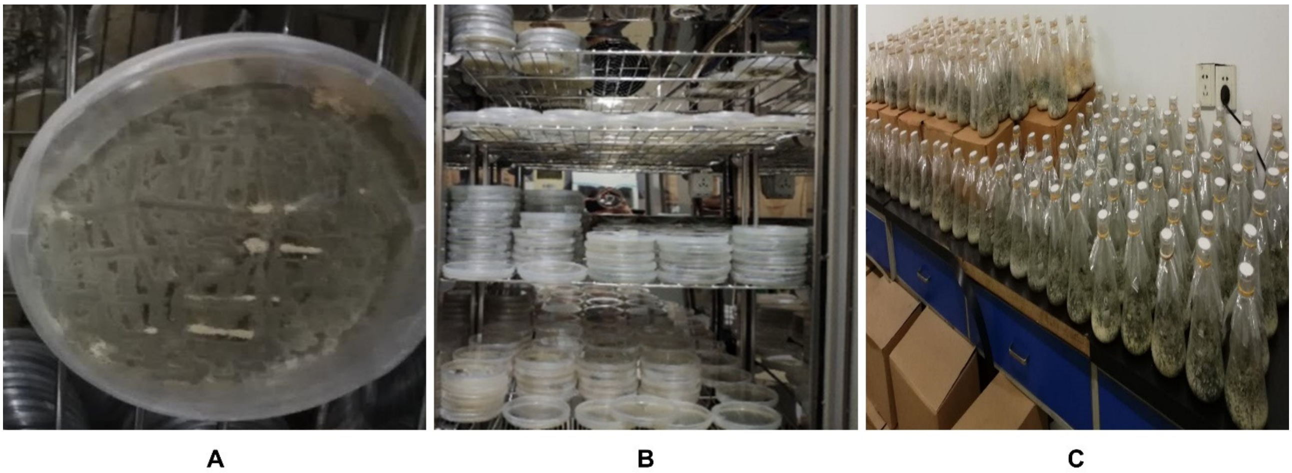

Figure S1. The Coculture and the fermentation of fungi. (A) Co-cultured fungi in a plate. (B) co-inoculated on the potato dextrose agar (PDA) in a 25°C incubator for 7 days. (C) the fermentation of fungi in a temperature-controlled room at 25°C for 30 days.

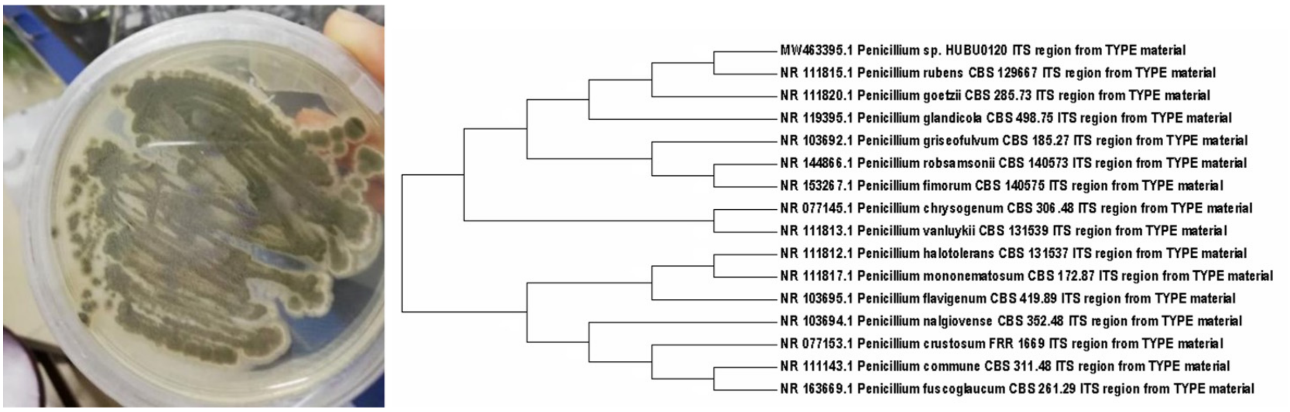

Figure S2. *Penicillium* sp. HUBU 0120 and the phylogenetic tree of ITS genes.

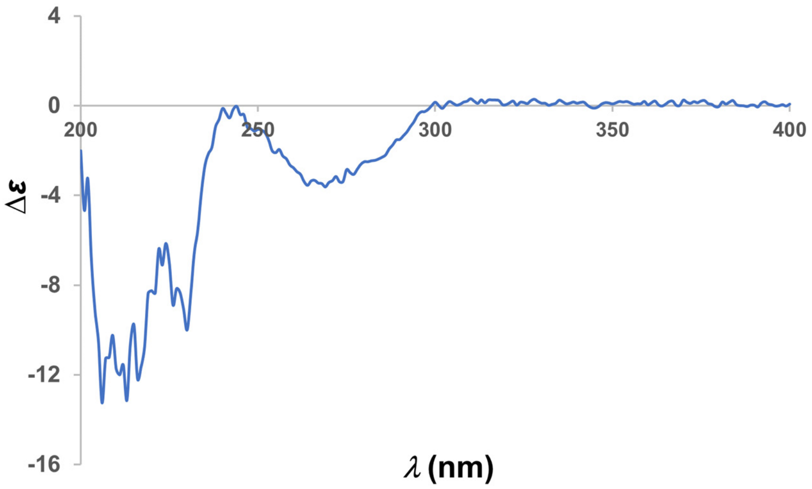

Figure S3. The experimental ECD spectrum of **1**.

Table S1. Deviations between the calculated and the experimental ^{13}C NMR chemical shifts for **2**.

**C**	**exp.**	**2**			
		**calc.**	**scal.calc.**	$\Delta\delta$	$\|\Delta\delta\|$
2	58.46	60.20	59.22	0.76	0.76
4	163.01	163.14	162.11	-0.90	0.90
5	60.14	61.92	60.94	0.80	0.80
7	166.56	163.50	162.47	-4.09	4.09
8	48.87	50.75	49.78	0.91	0.91
9	36.37	37.55	36.58	0.21	0.21
10	91.13	95.85	94.85	3.72	3.72
11	198.37	199.75	198.70	0.33	0.33
12	119.57	120.64	119.63	0.06	0.06
13	125.53	126.07	125.05	-0.48	0.48
14	120.33	122.32	121.31	0.98	0.98
15	138.69	138.00	136.97	-1.72	1.72
16	110.73	113.45	112.44	1.71	1.71
17	157.69	158.08	157.05	-0.64	0.64
18	117.92	119.45	118.44	0.52	0.52
19	139.36	143.63	142.61	3.25	3.25
20	26.06	25.38	24.42	-1.64	1.64
21	18.78	19.08	18.11	-0.68	0.68
22	32.15	33.67	32.70	0.55	0.55
23	18.66	18.33	17.37	-1.29	1.29
24	16.01	15.83	14.87	-1.14	1.14
25	51.96	51.82	50.84	-1.12	1.12
				AveDev	1.25
				MaxDev	4.09

Table S2. Primer information of Keap1, HO-1, NQO-1, and β-Actin in the present study.

**Primer Name**	**Accession Number**	**Primer sequence（5' – 3'）**		**Tm (°C)**	**CG%**	**Amplicon Length**
Keap1	NM_012289.3	Sense	CCAATGCTGACACGAAGGAT	58.1	50	194
		Antisense	ATACAGTTGTGCAGGACGCAG	58.6	52.4	
HO-1	NM_002133.2	Sense	GCCAGCAACAAAGTGCAAGA	59.6	50	100
		Antisense	TAAGGACCCATCGGAGAAGC	59.2	55	
NQO1	NM_000903.2	Sense	TGGTGGAGTCGGACCTCTATG	59.7	57.1	287
		Antisense	CATGGCAGCGTAAGTGTAAGC	58.8	52.4	
β-Actin	NM_001101	Sense	GTCCACCGCAAATGCTTCTA	58.7	50	190
		Antisense	TGCTGTCACCTTCACCGTTC	58.9	55	

Quantum-chemical computation for ECD

Conformational analyses were carried out whereby both BALLOON and confab programs [1,2] to confirm the stereochemistry structure of **2**. The BALLOON program explores conformational spaces with a genetic algorithm, and synchronously, the confab program systematically generates diverse low energy conformations that are proposed to be close to crystal structures. The conformations generated by the above programs were assembled via the removal of duplicated conformations whose root mean square (RMS) distance was less than 0.5 Å. Semi-empirical PM3 quantum mechanical geometry optimizations were fulfilled on conformations through the Gaussian 16 Revision A.03 [3]. Duplicated conformations after geometry optimization was subsequently identified and disposed. The remaining conformations were further optimized with PBE0 function applying 6-311G(d,p) basis set for C, N, and O atoms and 6-31G(d,p) basis set for H atoms in methanol solvent (IEFPCM solvation model) via Gaussian 16 program. Duplicated conformations emerged after these calculations were removed according to the same RMS criteria above. Harmonic vibrational frequencies were performed to establish the stability of the finally obtained conformers. Oscillator strengths and rotational strengths of 20 weakest electronic excitations of each conformer were calculated by the TDDFT methodology at the cam-b3lyp/def2tzvpp level for **2**, adopting methanol as solvent by the solvation model of IEFPCM for **2**, which were carried out in Gaussian 16 program. The ECD spectra data for each conformer were then simulated by using a Gaussian function with a bandwidth σ of 0.30 eV. Calculated spectra for each conformation were combined after Boltzmann weighting according to their population contribution.

1. M. J. Vainio, M. S. Johnson, **2007**. Generating conformer ensembles using a multiobjective genetic algorithm. *J. Chem. Inf. Model*, *47*, 2462–2474.

2. N. M.O′Boyle, T. Vandermeersch, C. J. Flynn, A. R. Maguire, G. R. Hutchison, **2011**. Confab-systematic generation of diverse low-energy conformers. *J. Cheminformatics*, *3*, 1–9.

3. Gaussian 16, Revision A.03, M. J. Frisch, G. W. Trucks, H. B. Schlegel, G. E. Scuseria, M. A. Robb, J. R. Cheeseman, G. Scalmani, V. Barone, G. A. Petersson, H. Nakatsuji, X. Li, M. Caricato, A. V. Marenich, J. Bloino, B. G. Janesko, R. Gomperts, B. Mennucci, H. P. Hratchian, J. V. Ortiz, A. F. Izmaylov, J. L. Sonnenberg, D. Williams-Young, F. Ding, F. Lipparini, F. Egidi, J. Goings, B. Peng, A. Petrone, T. Henderson, D. Ranasinghe, V. G. Zakrzewski, J. Gao, N. Rega, G. Zheng, W. Liang, M. Hada, M. Ehara, K. Toyota, R. Fukuda, J. Hasegawa, M. Ishida, T. Nakajima, Y. Honda, O. Kitao, H. Nakai, T. Vreven, K. Throssell, J. A. Montgomery, Jr., J. E. Peralta, F. Ogliaro, M. J. Bearpark, J. J. Heyd, E. N. Brothers, K. N. Kudin, V. N. Staroverov, T. A. Keith, R. Kobayashi, J. Normand, K. Raghavachari, A. P. Rendell, J. C. Burant, S. S. Iyengar, J. Tomasi, M. Cossi, J. M. Millam, M. Klene, C. Adamo, R. Cammi, J. W. Ochterski, R. L. Martin, K. Morokuma, O. Farkas, J. B. Foresman, and D. J. Fox, *Gaussian, Inc., Wallingford CT*, **2016**.

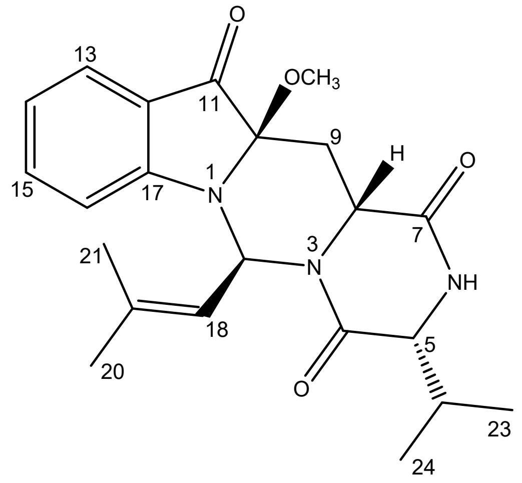

2*S*,5*R*,8*R*,10*R*-**2**

Optimized geometries of predominant conformers for compound 2*S*,5*R*,8*R*,10*R*-**2** with PBE0 function applying 6-311G(d,p) basis set for C, N, and O atoms and 6-31G(d,p) basis set for H atoms in methanol solvent.

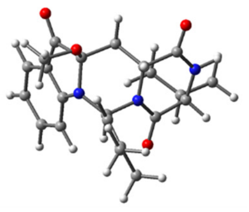

2*S*,5*R*,8*R*,10*R*-**2A**

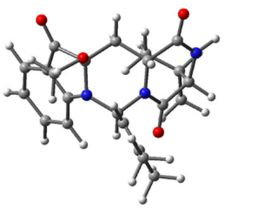

2*S*,5*R*,8*R*,10*R*-**2B**

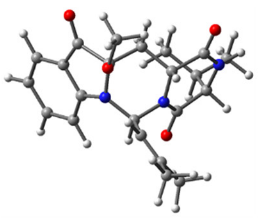

2*S*,5*R*,8*R*,10*R*-**2C**

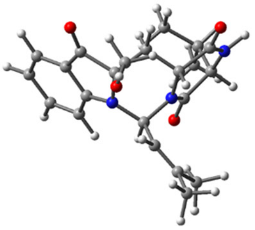

2*S*,5*R*,8*R*,10*R*-**2D**

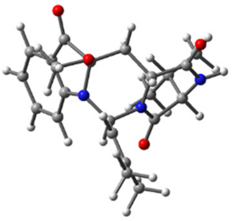

2*S*,5*R*,8*R*,10*R*-**2E**

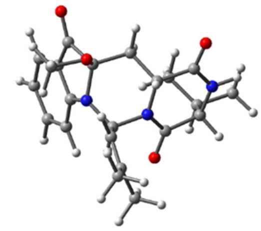

2*S*,5*R*,8*R*,10*R*-**2F**

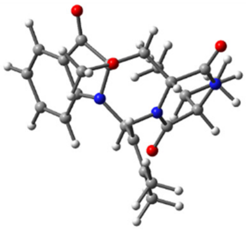

2*S*,5*R*,8*R*,10*R*-**2G**

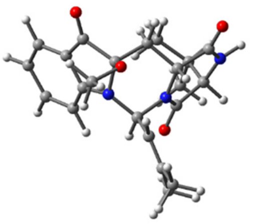

2*S*,5*R*,8*R*,10*R*-**2H**

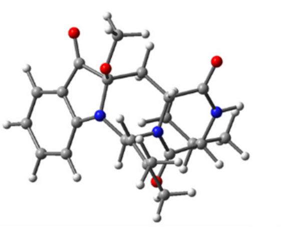

2*S*,5*R*,8*R*,10*R*-**2I**

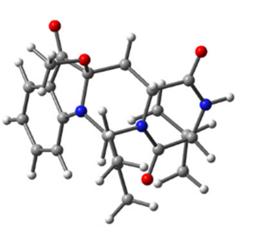

2*S*,5*R*,8*R*,10*R*-**2J**

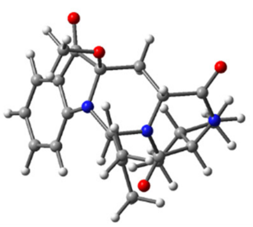

2*S*,5*R*,8*R*,10*R*-**2K**

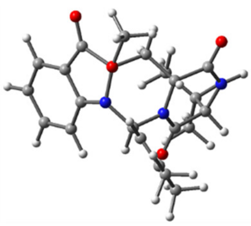

2*S*,5*R*,8*R*,10*R*-**2L**

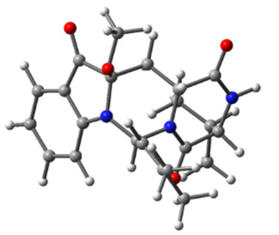

2*S*,5*R*,8*R*,10*R*-**2M**

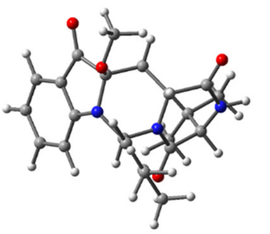

2*S*,5*R*,8*R*,10*R*-**2N**

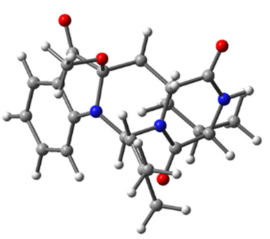

2*S*,5*R*,8*R*,10*R*-**2O**

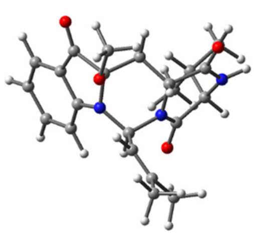

2*S*,5*R*,8*R*,10*R*-**2P**

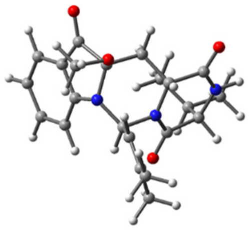

2*S*,5*R*,8*R*,10*R*-**2Q**

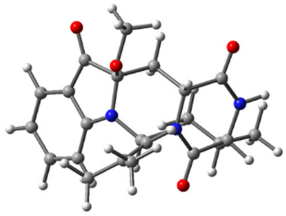

2*S*,5*R*,8*R*,10*R*-**2R**

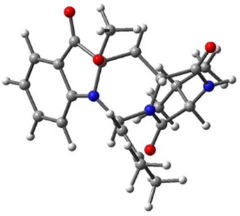

2*S*,5*R*,8*R*,10*R*-**2S**

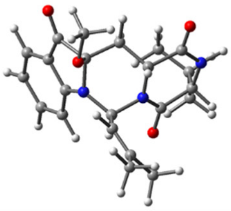

2*S*,5*R*,8*R*,10*R*-**2T**

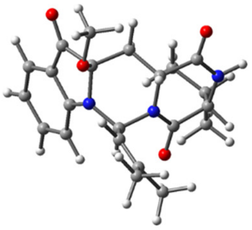

2*S*,5*R*,8*R*,10*R*-**2U**

Important thermodynamic parameters (a.u.) and Boltzmann distributions of the optimized compound 2*S*,5*R*,8*R*,10*R* -**2** with PBE0 function applying 6-311G(d,p) basis set for C, N, and O atoms and 6-31G(d,p) basis set for H atoms in methanol solvent.

Conformations	E+ZPE	G	%
2*S*,5*R*,8*R*,10*R*-**2A**	-1318.202186	-1318.259982	15.766
2*S*,5*R*,8*R*,10*R*-**2B**	-1318.200728	-1318.259150	6.531
2*S*,5*R*,8*R*,10*R*-**2C**	-1318.196436	-1318.254283	0.038
2*S*,5*R*,8*R*,10*R*-**2D**	-1318.194843	-1318.252635	0.007
2*S*,5*R*,8*R*,10*R*-**2E**	-1318.199226	-1318.257294	0.915
2*S*,5*R*,8*R*,10*R*-**2F**	-1318.202185	-1318.259981	15.749
2*S*,5*R*,8*R*,10*R*-**2G**	-1318.202184	-1318.259974	15.633
2*S*,5*R*,8*R*,10*R*-**2H**	-1318.200723	-1318.259146	6.504
2*S*,5*R*,8*R*,10*R*-**2I**	-1318.196435	-1318.254279	0.038
2*S*,5*R*,8*R*,10*R*-**2J**	-1318.200719	-1318.259135	6.428
2*S*,5*R*,8*R*,10*R*-**2K**	-1318.199227	-1318.257300	0.920
2*S*,5*R*,8*R*,10*R*-**2L**	-1318.194842	-1318.252638	0.007
2*S*,5*R*,8*R*,10*R*-**2M**	-1318.194845	-1318.252645	0.007
2*S*,5*R*,8*R*,10*R*-**2N**	-1318.193456	-1318.251800	0.003
2*S*,5*R*,8*R*,10*R*-**2O**	-1318.202183	-1318.259971	15.583
2*S*,5*R*,8*R*,10*R*-**2P**	-1318.193453	-1318.251785	0.003
2*S*,5*R*,8*R*,10*R*-**2Q**	-1318.202186	-1318.259987	15.849
2*S*,5*R*,8*R*,10*R*-**2R**	-1318.194199	-1318.252603	0.006
2*S*,5*R*,8*R*,10*R*-**2S**	-1318.193454	-1318.251786	0.003
2*S*,5*R*,8*R*,10*R*-**2T**	-1318.194842	-1318.252631	0.007
2*S*,5*R*,8*R*,10*R*-**2U**	-1318.194842	-1318.252634	0.007

E+ZPE, G: total energy with zero-point energy (ZPE) and Gibbs free energy in methanol solution. %: Boltzmann distributions, using the relative Gibbs free energies as weighting factors.

Optimized Z-matrixes (Å) of compound 2*S*,5*R*,8*R*,10*R* -**2** with PBE0 function applying 6-311G(d,p) basis set for C,

N, and O atoms and 6-31G(d,p) basis set for H atoms in methanol solvent.

2*S*,5*R*,8*R*,10*R*-**2A**					2*S*,5*R*,8*R*,10*R*-**2B**			
C	-2.801491	-3.267556	1.785982		C	-3.059642	-2.983611	1.734127
C	-2.109062	-2.398858	2.64081		C	-2.14244	-2.32808	2.566952
C	-1.59201	-1.184987	2.21066		C	-1.481131	-1.170248	2.181953
C	-1.784996	-0.829937	0.874113		C	-1.75865	-0.65005	0.91609
C	-2.447661	-1.715971	0.006722		C	-2.650722	-1.326302	0.065469
C	-2.972681	-2.925044	0.456618		C	-3.316787	-2.480054	0.471448
N	-1.418076	0.338317	0.228676		N	-1.27557	0.508129	0.332574
C	-1.541321	0.129696	-1.221894		C	-1.585666	0.471976	-1.104534
C	-2.434688	-1.134061	-1.322175		C	-2.685818	-0.617231	-1.199863
C	-0.330963	1.16496	0.717213		C	-0.020645	1.101395	0.753617
N	0.928209	0.695992	0.128543		N	1.071329	0.500221	-0.019596
C	0.925381	0.699171	-1.322295		C	0.916808	0.648514	-1.453729
C	-0.18439	-0.221127	-1.83477		C	-0.380187	-0.025819	-1.904235
O	-2.953841	-1.507243	-2.35062		O	-3.3704	-0.793959	-2.183006
O	-2.040809	1.235	-1.899834		O	-1.959683	1.705633	-1.623098
C	1.876644	0.159743	0.923521		C	2.029963	-0.203668	0.619074
C	3.111255	-0.419882	0.268276		C	3.072165	-0.908845	-0.222063
N	3.201084	-0.1491	-1.15134		N	3.060941	-0.493203	-1.611124
C	2.240307	0.31932	-1.962728		C	2.080632	0.140505	-2.273299
C	-0.57074	2.624105	0.438104		C	-0.045123	2.599936	0.6266
O	1.777683	0.110825	2.143122		O	2.076532	-0.2999	1.839295
O	2.380866	0.449159	-3.16881		O	2.09589	0.332919	-3.479352
C	-0.071445	3.625475	1.168687		C	0.615465	3.442818	1.426298
C	-0.371399	5.049833	0.81152		C	0.512492	4.923513	1.216361
C	0.815544	3.44813	2.362567		C	1.498639	3.029491	2.563564
C	3.195835	-1.92648	0.59728		C	2.949455	-2.448842	-0.108381
C	4.509279	-2.518255	0.10173		C	1.569331	-2.947449	-0.515852
C	1.999748	-2.700224	0.058139		C	3.349037	-2.968285	1.266808
H	0.721727	1.72355	-1.654122		H	0.853898	1.720649	-1.674429
H	3.955152	0.074894	0.761264		H	4.034286	-0.616827	0.21095
C	-3.349918	1.622495	-1.502827		C	-3.128752	2.258782	-1.032791
H	-3.186726	-4.204519	2.167643		H	-3.550883	-3.884728	2.078551
H	-1.968586	-2.682288	3.67784		H	-1.937733	-2.738145	3.549483
H	-1.055648	-0.548106	2.90148		H	-0.774105	-0.700787	2.853113
H	-3.485313	-3.5817	-0.236669		H	-4.004537	-2.972609	-0.206076
H	-0.232888	0.997033	1.78662		H	0.160629	0.814877	1.785821
H	-0.266691	-0.148588	-2.919217		H	-0.561863	0.169465	-2.961003
H	0.066955	-1.253054	-1.574884		H	-0.284658	-1.105363	-1.762599
H	4.073921	-0.370632	-1.609423		H	3.822484	-0.821236	-2.189583
H	-1.182431	2.853768	-0.425925		H	-0.642892	3.001574	-0.183095
H	-1.021605	5.124343	-0.061496		H	-0.14976	5.173989	0.386209
H	0.554692	5.596719	0.604173		H	1.500247	5.352387	1.016131
H	-0.854786	5.560785	1.651212		H	0.137184	5.412493	2.121843

H	1.024353	2.406821	2.608105		H	1.604092	1.950139	2.671649
H	0.372141	3.936282	3.23688		H	1.121315	3.446868	3.503321
H	1.774721	3.946265	2.183388		H	2.500505	3.448346	2.419757
H	3.181464	-1.973367	1.690436		H	3.677441	-2.835301	-0.832751
H	4.549164	-2.546986	-0.991816		H	0.796628	-2.591217	0.174178
H	5.369412	-1.947709	0.463472		H	1.309405	-2.630577	-1.528965
H	4.614596	-3.546718	0.454332		H	1.543955	-4.039241	-0.500459
H	2.085215	-3.757226	0.319643		H	3.388978	-4.060283	1.248603
H	1.05485	-2.336378	0.473308		H	4.334936	-2.597907	1.560533
H	1.951359	-2.6358	-1.033398		H	2.631318	-2.659678	2.028341
H	-3.561806	2.55997	-2.014997		H	-3.236998	3.260663	-1.445782
H	-4.088746	0.872737	-1.800608		H	-4.013667	1.666707	-1.284062
H	-3.401583	1.778243	-0.420761		H	-3.026358	2.321488	0.054957

2*S*,5*R*,8*R*,10*R*-**2C**				2*S*,5*R*,8*R*,10*R*-**2D**			
C	3.853521	-2.429997	-1.655874	C	3.981059	-2.166577	-1.50137
C	2.97017	-1.857372	-2.581729	C	2.924695	-1.958382	-2.399077
C	2.082264	-0.847163	-2.239927	C	1.921824	-1.027134	-2.168544
C	2.088105	-0.393531	-0.91911	C	1.989153	-0.274101	-0.994371
C	2.95197	-0.986387	0.017567	C	3.02933	-0.503975	-0.077863
C	3.845459	-1.991499	-0.343572	C	4.034922	-1.433779	-0.328891
N	1.350453	0.624975	-0.351666	N	1.156373	0.741629	-0.569549
C	1.494406	0.580365	1.099469	C	1.44227	1.0459	0.82962
C	2.695311	-0.377843	1.31016	C	2.806589	0.351012	1.074097
C	0.112258	1.115975	-0.922947	C	-0.199387	0.89778	-1.058501
N	-1.018686	0.435079	-0.28216	N	-1.11921	0.197179	-0.155273
C	-1.026619	0.567469	1.161983	C	-1.019217	0.643066	1.219991
C	0.250763	-0.05294	1.729995	C	0.397637	0.380556	1.731974
O	3.247762	-0.552458	2.375382	O	3.486906	0.515261	2.064244
O	1.765872	1.888747	1.53101	O	1.517351	2.442125	0.94739
C	-1.782457	-0.416332	-0.998117	C	-1.797044	-0.885831	-0.593037
C	-2.887881	-1.160865	-0.278742	C	-2.672076	-1.62616	0.397754
N	-3.06474	-0.758193	1.101032	N	-2.809798	-0.938502	1.66734
C	-2.237287	-0.015167	1.851425	C	-2.039536	0.044369	2.158203
C	0.004469	2.615647	-0.803621	C	-0.563345	2.353198	-1.216251
O	-1.632393	-0.600741	-2.199031	O	-1.746144	-1.271208	-1.754417
O	-2.416616	0.207145	3.038878	O	-2.133196	0.473404	3.298075
C	-0.871701	3.364007	-1.479773	C	-1.621547	2.798084	-1.899858
C	-0.910187	4.850385	-1.292201	C	-1.903922	4.266918	-1.995229
C	-1.881414	2.8182	-2.442176	C	-2.608841	1.914436	-2.598131
C	-2.66666	-2.680643	-0.433967	C	-2.198069	-3.083965	0.614185
C	-3.849643	-3.463308	0.121916	C	-0.751446	-3.153999	1.084493
C	-1.356302	-3.136473	0.194062	C	-2.434524	-3.965097	-0.605845
H	-1.042714	1.638748	1.394915	H	-1.211324	1.722971	1.23039
H	-3.80152	-0.901956	-0.825771	H	-3.659402	-1.663063	-0.074957

C	1.728109	2.129022	2.931691	C	1.558367	2.978871	2.263002
H	4.527784	-3.216388	-1.970278	H	4.7401	-2.90536	-1.724904
H	2.97708	-2.214681	-3.605376	H	2.884894	-2.546014	-3.309365
H	1.415708	-0.434358	-2.985544	H	1.121321	-0.901791	-2.88555
H	4.50265	-2.422384	0.402881	H	4.82655	-1.581219	0.396589
H	0.077132	0.811535	-1.967447	H	-0.278495	0.378936	-2.012013
H	0.27399	0.046197	2.815225	H	0.501628	0.720402	2.762293
H	0.256031	-1.122214	1.499575	H	0.581833	-0.697289	1.722527
H	-3.878203	-1.112162	1.584578	H	-3.483282	-1.31925	2.318233
H	0.69869	3.082306	-0.115745	H	0.100378	3.05692	-0.729539
H	-0.169334	5.189836	-0.566659	H	-1.166562	4.860825	-1.452912
H	-1.901704	5.16812	-0.951445	H	-2.897178	4.493299	-1.59226
H	-0.728146	5.362196	-2.243399	H	-1.910878	4.589188	-3.042112
H	-1.737479	1.76376	-2.679049	H	-2.305426	0.868384	-2.6447
H	-1.860881	3.391446	-3.3745	H	-2.780577	2.274671	-3.617488
H	-2.889822	2.933356	-2.028315	H	-3.576508	1.959856	-2.085224
H	-2.615069	-2.845145	-1.51448	H	-2.835915	-3.461978	1.423045
H	-3.910041	-3.379277	1.211661	H	-0.065301	-2.787984	0.313033
H	-4.796478	-3.119501	-0.303976	H	-0.596075	-2.57542	1.998616
H	-3.740621	-4.524623	-0.112344	H	-0.475923	-4.187901	1.303717
H	-1.227979	-4.212948	0.060992	H	-2.223185	-5.006894	-0.352043
H	-0.492149	-2.645515	-0.26336	H	-3.472122	-3.903153	-0.945279
H	-1.345869	-2.935389	1.26992	H	-1.789319	-3.671036	-1.434792
H	2.177227	3.111652	3.073478	H	1.834291	4.026332	2.144583
H	0.700587	2.15754	3.309839	H	0.579094	2.929233	2.750899
H	2.305018	1.385968	3.485197	H	2.305134	2.477397	2.881144

2*S*,5*R*,8*R*,10*R*-**2E**				2*S*,5*R*,8*R*,10*R*-**2F**			
C	3.238492	-2.914398	-1.565353	C	2.801831	-3.267321	-1.785944
C	2.479185	-2.172554	-2.480957	C	2.109204	-2.398788	-2.640778
C	1.791042	-1.022876	-2.120103	C	1.592029	-1.18495	-2.210682
C	1.874342	-0.601265	-0.791355	C	1.785099	-0.829763	-0.874183
C	2.607316	-1.36289	0.135632	C	2.447964	-1.715635	-0.00678
C	3.304127	-2.507422	-0.244936	C	2.973101	-2.924676	-0.456624
N	1.332119	0.530728	-0.208342	N	1.418101	0.338506	-0.22881
C	1.425548	0.395941	1.254028	C	1.541474	0.129999	1.221769
C	2.464037	-0.74114	1.438126	C	2.435033	-1.133616	1.322071
C	0.168249	1.192932	-0.772835	C	0.330816	1.164945	-0.717314
N	-1.034882	0.591102	-0.196175	N	-0.928231	0.695796	-0.128537
C	-1.079511	0.677036	1.251088	C	-0.925318	0.69908	1.322303
C	0.099582	-0.08915	1.849824	C	0.18464	-0.221	1.834759
O	2.981894	-1.00836	2.499862	O	2.954332	-1.506637	2.350501
O	1.761964	1.582363	1.895371	O	2.040835	1.235419	1.899603
C	-1.838705	-0.18149	-0.961155	C	-1.876699	0.15948	-0.923424
C	-2.860662	-1.021927	-0.222747	C	-3.111193	-0.420265	-0.268066

N	-3.238226	-0.408641	1.037433	N	-3.200917	-0.149519	1.151563
C	-2.398058	0.248768	1.857409	C	-2.240127	0.31904	1.962855
C	0.217666	2.682507	-0.561394	C	0.570385	2.624146	-0.438297
O	-1.73779	-0.246436	-2.178482	O	-1.777849	0.110574	-2.143034
O	-2.659811	0.535449	3.013949	O	-2.380593	0.448869	3.168949
C	-0.446383	3.57066	-1.306646	C	0.070704	3.625402	-1.168773
C	-0.336068	5.037932	-1.022857	C	0.370474	5.049829	-0.811725
C	-1.347865	3.211283	-2.447504	C	-0.816604	3.44785	-2.362382
C	-2.343445	-2.471519	-0.034419	C	-3.195676	-1.926864	-0.597085
C	-1.998822	-3.143615	-1.3582	C	-4.509083	-2.518716	-0.101531
C	-3.372119	-3.299035	0.729378	C	-1.999538	-2.700547	-0.057959
H	-0.978446	1.735469	1.515759	H	-0.721813	1.723516	1.65405
H	-3.747137	-1.051607	-0.861625	H	-3.955183	0.074438	-0.760968
C	3.032251	2.105824	1.529529	C	3.349864	1.623079	1.502488
H	3.758408	-3.805242	-1.894028	H	3.187159	-4.204262	-2.167565
H	2.424173	-2.506114	-3.511097	H	1.968668	-2.682325	-3.677771
H	1.210549	-0.484566	-2.857548	H	1.055517	-0.548198	-2.901502
H	3.866375	-3.067015	0.493564	H	3.485887	-3.581208	0.236667
H	0.130403	0.96392	-1.835332	H	0.232705	0.996955	-1.786711
H	0.14123	0.048003	2.930723	H	0.267	-0.148372	2.919195
H	-0.018451	-1.156075	1.641294	H	-0.066557	-1.252985	1.57496
H	-4.119159	-0.682809	1.447927	H	-4.073675	-0.37117	1.609738
H	0.828638	3.032301	0.261558	H	1.182238	2.853947	0.42558
H	0.330819	5.242828	-0.183909	H	1.020716	5.124497	0.061251
H	-1.320998	5.460085	-0.796043	H	-0.555677	5.596612	-0.604381
H	0.03724	5.571074	-1.903875	H	0.853758	5.560773	-1.651482
H	-1.402074	2.140921	-2.647445	H	-1.024819	2.406479	-2.608159
H	-1.024328	3.724838	-3.35905	H	-0.373877	3.936582	-3.236709
H	-2.363936	3.563509	-2.237869	H	-1.776054	3.945267	-2.18266
H	-1.431754	-2.41328	0.573303	H	-3.181311	-1.973731	-1.690242
H	-2.875739	-3.1825	-2.011926	H	-4.548959	-2.547446	0.992015
H	-1.202987	-2.623873	-1.892223	H	-5.369251	-1.948215	-0.463263
H	-1.67335	-4.169719	-1.171109	H	-4.614345	-3.547184	-0.454134
H	-3.01211	-4.323535	0.846632	H	-2.084938	-3.757545	-0.319503
H	-3.563854	-2.903437	1.728999	H	-1.054657	-2.336628	-0.473098
H	-4.320354	-3.337505	0.182907	H	-1.951168	-2.636162	1.033581
H	3.104554	3.089465	1.991413	H	3.561603	2.560675	2.014499
H	3.840792	1.470553	1.902756	H	4.088829	0.873494	1.800367
H	3.117171	2.206261	0.442925	H	3.401461	1.778664	0.420396

2*S*,5*R*,8*R*,10*R*-**2G**				2*S*,5*R*,8*R*,10*R*-**2H**			
C	-2.800303	-3.268316	1.786175	C	-3.061735	-2.98085	1.731785
C	-2.108091	-2.399379	2.640935	C	-2.142948	-2.327865	2.564857
C	-1.59146	-1.185353	2.210714	C	-1.480641	-1.170145	2.181225
C	-1.784648	-0.830413	0.874169	C	-1.758813	-0.647408	0.916561

C	-2.447094	-1.716669	0.006848	C	-2.652413	-1.321171	0.065594
C	-2.971707	-2.925893	0.456815	C	-3.319451	-2.474841	0.4702
N	-1.418167	0.337968	0.228687	N	-1.274846	0.511224	0.334563
C	-1.541454	0.129276	-1.22186	C	-1.585734	0.477376	-1.102478
C	-2.434459	-1.134746	-1.322053	C	-2.687575	-0.610063	-1.198603
C	-0.331191	1.164879	0.717105	C	-0.018538	1.101654	0.755476
N	0.928016	0.696125	0.128373	N	1.071829	0.498694	-0.018575
C	0.925146	0.699309	-1.32247	C	0.917223	0.649277	-1.452452
C	-0.184494	-0.221196	-1.834872	C	-0.381498	-0.021407	-1.903542
O	-2.953623	-1.50808	-2.350435	O	-3.373207	-0.784308	-2.181452
O	-2.041323	1.234418	-1.899806	O	-1.958143	1.712224	-1.619297
C	1.876791	0.160495	0.923342	C	2.028077	-0.209582	0.618824
C	3.111449	-0.418988	0.268057	C	3.068284	-0.916181	-0.223589
N	3.201011	-0.148567	-1.151643	N	3.058128	-0.498113	-1.611937
C	2.240082	0.319626	-1.962968	C	2.07962	0.139575	-2.272967
C	-0.571338	2.62395	0.437944	C	-0.039908	2.600353	0.629368
O	1.77805	0.11188	2.142975	O	2.074235	-0.308295	1.838869
O	2.380487	0.449359	-3.169086	O	2.095406	0.334093	-3.478678
C	-0.072528	3.62549	1.168627	C	0.625633	3.441035	1.427265
C	-0.372925	5.049743	0.8114	C	0.525815	4.922122	1.218626
C	0.81431	3.448477	2.362668	C	1.511771	3.024581	2.561064
C	3.196396	-1.925487	0.597406	C	2.94157	-2.456048	-0.112541
C	4.509945	-2.51706	0.101907	C	1.560199	-2.950419	-0.520914
C	2.000451	-2.699625	0.058495	C	3.339719	-2.978907	1.26177
H	0.721315	1.723651	-1.654301	H	0.856806	1.72187	-1.671598
H	3.955309	0.076093	0.7608	H	4.031204	-0.627457	0.20987
C	-3.350437	1.62166	-1.502599	C	-3.126137	2.266359	-1.027786
H	-3.185217	-4.20539	2.167887	H	-3.553698	-3.881994	2.075104
H	-1.967458	-2.682738	3.677962	H	-1.937727	-2.739917	3.546448
H	-1.055273	-0.548269	2.901482	H	-0.772174	-0.702841	2.852365
H	-3.484186	-3.582733	-0.23641	H	-4.008351	-2.965474	-0.207548
H	-0.233011	0.997042	1.786518	H	0.162516	0.81417	1.78746
H	-0.266924	-0.148637	-2.919307	H	-0.563232	0.176157	-2.959877
H	0.067124	-1.253073	-1.57505	H	-0.288173	-1.101408	-1.763965
H	4.073824	-0.370035	-1.609803	H	3.818823	-0.82713	-2.190959
H	-1.182914	2.853432	-0.426217	H	-0.639392	3.003954	-0.178061
H	-1.022734	5.124025	-0.06193	H	-0.13825	5.174834	0.390603
H	0.553021	5.597063	0.604559	H	1.514053	5.348643	1.01577
H	-0.856968	5.560406	1.650891	H	0.154419	5.411435	2.125543
H	1.023235	2.407237	2.608403	H	1.613365	1.944855	2.66917
H	0.370743	3.936737	3.236831	H	1.139936	3.443761	3.502202
H	1.773457	3.946682	2.183512	H	2.514711	3.4394	2.413046
H	3.182102	-1.972127	1.690574	H	3.668571	-2.843154	-0.837552
H	4.549784	-2.546003	-0.991635	H	0.788379	-2.593389	0.169696
H	5.369969	-1.946245	0.463488	H	1.301163	-2.631192	-1.53351

H	4.615511	-3.545426	0.454717	H	1.532029	-4.042166	-0.507329
H	2.086162	-3.756544	0.320261	H	3.376915	-4.070969	1.241641
H	1.055479	-2.335888	0.473587	H	4.326516	-2.611549	1.556273
H	1.952026	-2.635485	-1.033057	H	2.62268	-2.669839	2.023756
H	-3.562909	2.558646	-2.015421	H	-3.233298	3.268761	-1.439792
H	-4.089088	0.871383	-1.799524	H	-4.011988	1.675701	-1.279084
H	-3.401773	1.778205	-0.420634	H	-3.023039	2.327904	0.059963

2*S*,5*R*,8*R*,10*R*-**2I**				2*S*,5*R*,8*R*,10*R*-**2J**			
C	3.852972	-2.430442	-1.656218	C	3.061849	-2.979634	-1.730497
C	2.969698	-1.857506	-2.58195	C	2.141842	-2.328297	-2.563503
C	2.081998	-0.847175	-2.239965	C	1.479235	-1.170453	-2.180744
C	2.087974	-0.393738	-0.919081	C	1.758409	-0.64586	-0.917073
C	2.951771	-0.986901	0.01747	C	2.653191	-1.318017	-0.066089
C	3.845045	-1.992135	-0.343848	C	3.320496	-2.471833	-0.469819
N	1.35051	0.624795	-0.351445	N	1.274271	0.513244	-0.336064
C	1.49445	0.579858	1.099689	C	1.586023	0.480928	1.100877
C	2.695225	-0.378551	1.310178	C	2.688908	-0.605427	1.197266
C	0.112437	1.116149	-0.922649	C	0.017033	1.101953	-0.756587
N	-1.01869	0.43538	-0.282006	N	-1.072159	0.49783	0.01819
C	-1.026559	0.567401	1.162169	C	-0.91724	0.649746	1.451888
C	0.250701	-0.053419	1.730013	C	0.382752	-0.018468	1.903077
O	3.247673	-0.553399	2.375361	O	3.375542	-0.778001	2.179707
O	1.766162	1.888083	1.531536	O	1.957569	1.716528	1.616449
C	-1.782536	-0.415826	-0.99813	C	-2.027106	-0.213017	-0.618304
C	-2.888129	-1.160282	-0.278924	C	-3.065674	-0.920901	0.225044
N	-3.064935	-0.757852	1.100933	N	-3.05593	-0.501548	1.613017
C	-2.237349	-0.015136	1.851486	C	-2.078458	0.138631	2.273193
C	0.004921	2.615817	-0.803064	C	0.036472	2.600738	-0.630989
O	-1.632444	-0.600064	-2.199062	O	-2.073375	-0.312934	-1.838245
O	-2.416646	0.206973	3.038981	O	-2.094287	0.334206	3.478729
C	-0.870704	3.364512	-1.479555	C	-0.632192	3.440073	-1.427688
C	-0.908981	4.850857	-1.291683	C	-0.534304	4.921393	-1.219823
C	-1.879973	2.819157	-2.442675	C	-1.520262	3.021703	-2.559259
C	-2.667222	-2.680069	-0.434437	C	-2.936003	-2.46061	0.115326
C	-3.850366	-3.462612	0.121278	C	-1.553664	-2.951991	0.524034
C	-1.356967	-3.136285	0.193532	C	-3.333229	-2.98545	-1.258499
H	-1.042432	1.638622	1.395382	H	-0.858561	1.722613	1.670155
H	-3.801707	-0.901068	-0.825914	H	-4.029276	-0.63444	-0.208402
C	1.728082	2.128122	2.932249	C	3.124736	2.271245	1.023844
H	4.527065	-3.216923	-1.970761	H	3.553979	-3.880956	-2.073108
H	2.976492	-2.214664	-3.605651	H	1.935831	-2.741804	-3.544316
H	1.415489	-0.434143	-2.985497	H	0.76963	-0.704591	-2.851679
H	4.502177	-2.423259	0.402518	H	4.010292	-2.961208	0.207928
H	0.077268	0.811902	-1.967199	H	-0.164116	0.813923	-1.788409

H	0.273913	0.045346	2.815275	H	0.564704	0.180594	2.959094
H	0.255789	-1.122615	1.499228	H	0.290879	-1.098771	1.764893
H	-3.878501	-1.111717	1.58438	H	-3.815788	-0.831563	2.192572
H	0.698873	3.082184	-0.114717	H	0.637056	3.005528	0.175013
H	-0.168292	5.19002	-0.565839	H	0.130886	5.175463	-0.39312
H	-1.900546	5.168699	-0.951162	H	-1.522826	5.346467	-1.015326
H	-0.726556	5.362839	-2.242715	H	-0.165328	5.410918	-2.127613
H	-1.736581	1.764565	-2.679192	H	-1.619303	1.941753	-2.667505
H	-1.85826	3.392179	-3.375116	H	-1.152076	3.442196	-3.501239
H	-2.888646	2.935129	-2.029692	H	-2.523897	3.433816	-2.408382
H	-2.615638	-2.844379	-1.51498	H	-3.662217	-2.848487	0.840714
H	-3.910739	-3.378801	1.211042	H	-0.782534	-2.59395	-0.166829
H	-4.797136	-3.118518	-0.30453	H	-1.29526	-2.63155	1.536409
H	-3.741564	-4.523897	-0.113217	H	-1.523368	-4.04369	0.511215
H	-1.228846	-4.212755	0.060234	H	-3.368444	-4.077558	-1.23735
H	-0.492704	-2.645385	-0.263748	H	-4.320694	-2.62016	-1.553333
H	-1.346532	-2.935433	1.269433	H	-2.61676	-2.675798	-2.020782
H	2.177177	3.110726	3.074283	H	3.231171	3.274103	1.434927
H	0.700481	2.156589	3.310181	H	4.011268	1.681631	1.275185
H	2.304869	1.38497	3.485756	H	3.021009	2.331745	-0.063904

2*S*,5*R*,8*R*,10*R*-**2K**				2*S*,5*R*,8*R*,10*R*-**2L**			
C	3.236856	-2.915615	-1.565526	C	3.979859	-2.166904	-1.501616
C	2.477816	-2.173402	-2.48105	C	2.922947	-1.95935	-2.398819
C	1.79022	-1.023419	-2.120121	C	1.920263	-1.027875	-2.168377
C	1.873823	-0.601875	-0.791371	C	1.988376	-0.27393	-0.994837
C	2.606522	-1.363855	0.135538	C	3.029068	-0.503204	-0.078772
C	3.302783	-2.508696	-0.245103	C	4.034468	-1.433238	-0.329713
N	1.332129	0.530325	-0.208273	N	1.155915	0.742217	-0.570286
C	1.425563	0.395414	1.254093	C	1.442382	1.047018	0.82869
C	2.463595	-0.742094	1.438067	C	2.806988	0.352564	1.072743
C	0.168534	1.193044	-0.772686	C	-0.200144	0.897842	-1.058561
N	-1.034836	0.591581	-0.196113	N	-1.119255	0.196936	-0.154827
C	-1.079449	0.677375	1.25116	C	-1.018836	0.643064	1.220325
C	0.099423	-0.089175	1.849895	C	0.398419	0.381461	1.731698
O	2.981423	-1.009565	2.499754	O	3.487938	0.517605	2.062317
O	1.762499	1.581659	1.895488	O	1.517058	2.443262	0.94606
C	-1.838868	-0.180704	-0.961199	C	-1.796897	-0.886346	-0.592203
C	-2.860875	-1.021131	-0.222855	C	-2.67098	-1.627102	0.399092
N	-3.238357	-0.407906	1.037381	N	-2.808178	-0.939598	1.668819
C	-2.39811	0.24934	1.85741	C	-2.038233	0.043781	2.159161
C	0.218524	2.682574	-0.561061	C	-0.564717	2.353109	-1.216246
O	-1.738034	-0.24543	-2.178541	O	-1.746503	-1.271701	-1.753616
O	-2.659865	0.536031	3.013945	O	-2.131456	0.472785	3.299081
C	-0.444848	3.571133	-1.306429	C	-1.623222	2.797576	-1.899654

C	-0.333925	5.038318	-1.02241	C	-1.906157	4.266307	-1.994984
C	-1.346089	3.212373	-2.447667	C	-2.610317	1.913594	-2.59778
C	-2.34363	-2.470743	-0.03468	C	-2.196179	-3.084708	0.615099
C	-1.999478	-3.142845	-1.358582	C	-0.749153	-3.15416	1.084252
C	-3.372	-3.29827	0.729498	C	-2.433192	-3.965887	-0.604786
H	-0.978123	1.735754	1.515939	H	-1.211609	1.72285	1.230699
H	-3.747383	-1.050735	-0.861685	H	-3.658592	-1.664414	-0.072992
C	3.032969	2.104636	1.529588	C	1.558255	2.980381	2.261516
H	3.756328	-3.806694	-1.894262	H	4.738721	-2.905901	-1.725052
H	2.422565	-2.506918	-3.511191	H	2.882535	-2.547702	-3.308615
H	1.209904	-0.484838	-2.857507	H	1.119252	-0.903127	-2.884917
H	3.86483	-3.068563	0.493341	H	4.826508	-1.580198	0.395415
H	0.130608	0.964173	-1.835207	H	-0.279539	0.378934	-2.01201
H	0.141107	0.04799	2.930791	H	0.502675	0.721652	2.761876
H	-0.018966	-1.156068	1.641417	H	0.583151	-0.696299	1.722518
H	-4.119355	-0.681944	1.44783	H	-3.480927	-1.320813	2.320198
H	0.829358	3.031981	0.262158	H	0.098812	3.057108	-0.729674
H	0.332814	5.242781	-0.183238	H	-1.16879	4.860501	-1.45299
H	-1.318717	5.460914	-0.795828	H	-2.899331	4.492358	-1.591629
H	0.039906	5.571394	-1.903245	H	-1.913641	4.588525	-3.04188
H	-1.400921	2.142056	-2.647671	H	-2.307011	0.867478	-2.643606
H	-1.021827	3.725753	-3.359055	H	-2.781592	2.273253	-3.617423
H	-2.362012	3.565264	-2.238443	H	-3.578195	1.959456	-2.085314
H	-1.431713	-2.412516	0.572698	H	-2.833242	-3.463027	1.424433
H	-2.876666	-3.181868	-2.011936	H	-0.06375	-2.788143	0.312132
H	-1.203915	-2.623023	-1.892933	H	-0.59322	-2.575293	1.998097
H	-1.673805	-4.168902	-1.171581	H	-0.473132	-4.18792	1.303519
H	-3.011881	-4.322741	0.84667	H	-2.221241	-5.00761	-0.351188
H	-3.563423	-2.902624	1.729157	H	-3.471064	-3.904354	-0.943457
H	-4.320417	-3.336831	0.183349	H	-1.78871	-3.671525	-1.434188
H	3.105776	3.088131	1.991706	H	1.833717	4.02792	2.142722
H	3.841284	1.46892	1.902542	H	0.579149	2.930482	2.74972
H	3.117777	2.20531	0.442997	H	2.305413	2.479375	2.879563

2*S*,5*R*,8*R*,10*R*-**2M**				2*S*,5*R*,8*R*,10*R*-**2N**			
C	-3.981357	-2.165586	1.500614	C	-4.272546	-1.629897	1.441795
C	-2.924589	-1.958627	2.398128	C	-3.32369	-1.287175	2.415171
C	-1.921423	-1.027575	2.168075	C	-2.237012	-0.468167	2.142643
C	-1.988878	-0.273445	0.994615	C	-2.103383	0.027671	0.84377
C	-3.029443	-0.502117	0.078248	C	-3.037302	-0.338564	-0.140666
C	-4.035324	-1.431736	0.328798	C	-4.129325	-1.151716	0.151329
N	-1.155849	0.742364	0.570412	N	-1.154412	0.894805	0.342457
C	-1.441878	1.047611	-0.828539	C	-1.251748	0.930984	-1.113848
C	-2.806679	0.353709	-1.073086	C	-2.617286	0.253549	-1.397488
C	0.200152	0.897419	1.059055	C	0.138209	1.098564	0.971669

N	1.119229	0.196471	0.155349	N	1.114762	0.215164	0.330967
C	1.01928	0.643055	-1.219694	C	1.203	0.407413	-1.103476
C	-0.397915	0.381987	-1.73149	C	-0.144568	0.07361	-1.741339
O	-3.487264	0.519138	-2.06285	O	-3.156936	0.239087	-2.483318
O	-1.516074	2.443928	-0.945555	O	-1.225684	2.281282	-1.496168
C	1.796261	-0.887288	0.592501	C	1.608699	-0.850196	1.002322
C	2.670402	-1.627963	-0.398807	C	2.365771	-1.874965	0.180608
N	2.808249	-0.940004	-1.668216	N	2.938567	-1.28094	-1.013669
C	2.038815	0.043843	-2.158421	C	2.345781	-0.332772	-1.761166
C	0.565128	2.35255	1.217181	C	0.555733	2.54691	0.92118
O	1.745341	-1.273071	1.753748	O	1.44448	-1.012897	2.203468
O	2.132591	0.473295	-3.298127	O	2.699929	-0.029556	-2.888684
C	1.624343	2.796471	1.899839	C	1.568971	3.062607	1.62263
C	1.907686	4.265092	1.995616	C	1.916899	4.515625	1.506571
C	2.611884	1.911886	2.596572	C	2.440666	2.270796	2.548198
C	2.19519	-3.085331	-0.615532	C	1.466435	-3.094968	-0.147704
C	0.748294	-3.154113	-1.085185	C	0.934756	-3.778388	1.106526
C	2.431515	-3.967043	0.6041	C	2.232075	-4.093513	-1.010156
H	1.212325	1.722797	-1.229653	H	1.420565	1.46869	-1.271795
H	3.65785	-1.665786	0.073578	H	3.189409	-2.216664	0.813386
C	-1.556854	2.981412	-2.26087	C	-1.091496	2.560183	-2.883768
H	-4.740612	-2.904268	1.723751	H	-5.103626	-2.272682	1.702576
H	-2.884687	-2.547114	3.307859	H	-3.438932	-1.674801	3.421161
H	-1.12056	-0.903269	2.884858	H	-1.527092	-0.230678	2.923843
H	-4.827243	-1.578233	-0.396556	H	-4.834306	-1.409015	-0.6307
H	0.279152	0.378228	2.012394	H	0.070593	0.756435	2.0032
H	-0.501848	0.722497	-2.761596	H	-0.112289	0.216783	-2.821723
H	-0.582966	-0.69572	-1.722644	H	-0.380909	-0.978695	-1.55863
H	3.481198	-1.321086	-2.319465	H	3.708289	-1.764145	-1.454143
H	-0.098713	3.056906	0.731557	H	-0.027608	3.180324	0.264483
H	1.170075	4.859696	1.454403	H	1.261694	5.038056	0.807761
H	2.900626	4.491087	1.591652	H	2.952133	4.63598	1.168943
H	1.915993	4.586848	3.042648	H	1.848105	5.006136	2.483584
H	2.307717	0.866057	2.643284	H	2.08795	1.253607	2.719893
H	2.785211	2.271717	3.615802	H	2.522489	2.779777	3.51402
H	3.578923	1.956701	2.082429	H	3.456742	2.210007	2.141818
H	2.832386	-3.463556	-1.424805	H	0.614041	-2.724052	-0.730679
H	0.062774	-2.788071	-0.313181	H	1.76012	-4.133202	1.731693
H	0.592886	-2.574915	-1.998909	H	0.31704	-3.113854	1.710688
H	0.471966	-4.187704	-1.304861	H	0.332821	-4.644535	0.821492
H	2.219226	-5.008593	0.350071	H	1.604562	-4.96337	-1.216203
H	3.469321	-3.90604	0.943071	H	2.529773	-3.670101	-1.971653
H	1.786922	-3.672723	1.433431	H	3.130565	-4.444114	-0.491364
H	-1.832104	4.028985	-2.141866	H	-1.321093	3.619625	-2.994098
H	-0.57765	2.931419	-2.74887	H	-0.068261	2.387003	-3.233752

H	-2.303992	2.480752	-2.879224	H	-1.791331	1.975408	-3.483283

2*S*,5*R*,8*R*,10*R*-**2O**				2*S*,5*R*,8*R*,10*R*-**2P**			
C	2.798459	-3.269702	-1.787471	C	4.271446	-1.630605	-1.442729
C	2.107391	-2.399475	-2.641838	C	3.322509	-1.287285	-2.415817
C	1.591437	-1.185437	-2.210828	C	2.236162	-0.467993	-2.142831
C	1.784146	-0.831829	-0.873859	C	2.102963	0.027512	-0.843788
C	2.445468	-1.719354	-0.006963	C	3.036936	-0.339342	0.140357
C	2.969398	-2.928577	-0.457713	C	4.128637	-1.152767	-0.152092
N	1.418165	0.336212	-0.227533	N	1.154406	0.894893	-0.342054
C	1.54066	0.126262	1.22289	C	1.251956	0.930448	1.114259
C	2.432562	-1.138596	1.322444	C	2.617371	0.252568	1.397425
C	0.332261	1.164597	-0.715847	C	-0.138309	1.099054	-0.970971
N	-0.927667	0.69693	-0.127776	N	-1.114865	0.215477	-0.330506
C	-0.925253	0.698838	1.323069	C	-1.202904	0.40715	1.104027
C	0.183115	-0.223408	1.835028	C	0.144712	0.07303	1.741593
O	2.950874	-1.513253	2.350777	O	3.157268	0.237688	2.483127
O	2.041202	1.230451	1.901899	O	1.226315	2.280574	1.497182
C	-1.877132	0.163712	-0.923554	C	-1.609056	-0.849493	-1.002292
C	-3.112786	-0.414643	-0.269151	C	-2.366099	-1.874552	-0.180921
N	-3.202476	-0.14511	1.150711	N	-2.938636	-1.281004	1.013718
C	-2.2409	0.320353	1.962852	C	-2.345658	-0.333237	1.761558
C	0.574016	2.623197	-0.435785	C	-0.555661	2.547413	-0.919789
O	-1.778179	0.116252	-2.143215	O	-1.445008	-1.011665	-2.203536
O	-2.381341	0.448822	3.169092	O	-2.69961	-0.030497	2.889267
C	0.078968	3.625796	-1.167573	C	-1.567843	3.063882	-1.6222
C	0.380922	5.04941	-0.809107	C	-1.915595	4.516893	-1.50547
C	-0.804747	3.450784	-2.364236	C	-2.438389	2.273117	-2.549729
C	-3.199578	-1.920801	-0.599666	C	-1.466813	-3.094788	0.146658
C	-4.513908	-2.511147	-0.104761	C	-0.936025	-3.778021	-1.108049
C	-2.00467	-2.696855	-0.061229	C	-2.232177	-4.093321	1.009363
H	-0.720288	1.722638	1.655897	H	-1.420432	1.468363	1.272817
H	-3.955827	0.081916	-0.761796	H	-3.189888	-2.215907	-0.81369
C	3.350757	1.616965	1.505426	C	1.091918	2.558801	2.884896
H	3.182861	-4.206733	-2.169803	H	5.102266	-2.273582	-1.703866
H	1.967126	-2.681792	-3.679201	H	3.437425	-1.674659	-3.421941
H	1.05621	-0.5473	-2.901372	H	1.526151	-0.230066	-2.923813
H	3.481012	-3.586403	0.235216	H	4.833685	-1.410535	0.629721
H	0.234214	0.997423	-1.78536	H	-0.070881	0.757376	-2.002647
H	0.265177	-0.151965	2.919565	H	0.112568	0.2159	2.822023
H	-0.069489	-1.254763	1.574101	H	0.380911	-0.979252	1.558569
H	-4.075783	-0.365652	1.608376	H	-3.708405	-1.764284	1.454034
H	1.183697	2.851417	0.430065	H	0.026867	3.180195	-0.261756
H	1.029122	5.122269	0.065536	H	-1.261215	5.038619	-0.805361
H	-0.544574	5.598083	-0.603836	H	-2.95126	4.637241	-1.169174

H	0.867404	5.559711	-1.647408	H	-1.845328	5.008154	-2.482003
H	-1.01684	2.410076	-2.609484	H	-2.086929	1.255284	-2.720147
H	-0.356481	3.936367	-3.237512	H	-2.51685	2.781901	-3.515949
H	-1.762524	3.952891	-2.188757	H	-3.455629	2.214212	-2.146011
H	-3.18522	-1.966629	-1.69287	H	-0.613991	-2.72417	0.729202
H	-4.553861	-2.540921	0.988754	H	-1.761838	-4.132638	-1.73274
H	-5.373198	-1.938992	-0.465956	H	-0.318669	-3.113406	-1.712492
H	-4.620706	-3.539099	-0.458408	H	-0.333973	-4.644277	-0.823597
H	-2.091792	-3.753502	-0.323621	H	-1.604861	-4.963509	1.214608
H	-1.059228	-2.334121	-0.47611	H	-2.52893	-3.670091	1.97123
H	-1.95616	-2.63344	1.030361	H	-3.131233	-4.443375	0.491177
H	3.563638	2.553605	2.018708	H	1.322605	3.61793	2.99596
H	4.088752	0.866052	1.802374	H	0.068364	2.38649	3.234384
H	3.402658	1.773914	0.423548	H	1.790927	1.972931	3.484307

2*S*,5*R*,8*R*,10*R*-**2Q**				2*S*,5*R*,8*R*,10*R*-**2R**			
C	2.803113	-3.266492	-1.785887	C	-2.430036	3.95866	-0.759712
C	2.110319	-2.398136	-2.640765	C	-1.990145	3.211382	-1.860828
C	1.592703	-1.184483	-2.210674	C	-1.561691	1.896	-1.747315
C	1.785485	-0.829303	-0.874131	C	-1.584858	1.309501	-0.479298
C	2.448527	-1.715007	-0.006691	C	-1.993923	2.068419	0.632224
C	2.974108	-2.923858	-0.45653	C	-2.430467	3.383561	0.49927
N	1.41803	0.338812	-0.228747	N	-1.277849	0.017505	-0.116144
C	1.541357	0.130317	1.221835	C	-1.180532	-0.073522	1.333388
C	2.435264	-1.133049	1.322183	C	-1.843033	1.242289	1.817012
C	0.330571	1.164964	-0.717332	C	-0.491779	-0.88396	-0.935548
N	-0.928402	0.695525	-0.128613	N	0.896841	-0.873023	-0.477351
C	-0.92555	0.69882	1.322227	C	1.067264	-1.10905	0.945028
C	0.184574	-0.221033	1.834723	C	0.296016	-0.055183	1.738096
O	2.954547	-1.505966	2.350659	O	-2.126125	1.480642	2.971925
O	2.040382	1.235868	1.899712	O	-1.870722	-1.227168	1.725218
C	-1.876689	0.158937	-0.923532	C	1.86968	-0.515874	-1.345014
C	-3.11113	-0.420978	-0.268223	C	3.285926	-0.399893	-0.824303
N	-3.201002	-0.150145	1.15138	N	3.457096	-0.896928	0.524539
C	-2.240324	0.318556	1.962724	C	2.500278	-1.179506	1.420238
C	0.569765	2.624234	-0.438329	C	-1.027917	-2.292696	-0.976981
O	-1.77774	0.109958	-2.143133	O	1.65009	-0.264283	-2.52099
O	-2.38087	0.448367	3.16881	O	2.728918	-1.501145	2.576048
C	0.069747	3.625354	-1.168759	C	-2.134648	-2.662002	-1.623491
C	0.36914	5.049865	-0.811737	C	-2.581203	-4.092196	-1.633308
C	-0.817597	3.447534	-2.362301	C	-3.032321	-1.709685	-2.351761
C	-3.195347	-1.927612	-0.597145	C	3.762641	1.060725	-0.983984
C	-4.508659	-2.519659	-0.10157	C	5.241298	1.190495	-0.640942
C	-1.999073	-2.701037	-0.057956	C	2.916358	2.029798	-0.168836
H	-0.722252	1.7233	1.653968	H	0.646324	-2.093922	1.180701

H	-3.955166	0.073557	-0.761217	H	3.88909	-1.022238	-1.493901
C	3.349356	1.623849	1.502724	C	-1.738452	-1.623073	3.084201
H	3.188786	-4.203292	-2.167506	H	-2.754194	4.982147	-0.898344
H	1.970007	-2.681661	-3.677792	H	-1.980198	3.67474	-2.840983
H	1.056098	-0.547859	-2.90154	H	-1.220603	1.360425	-2.623826
H	3.487023	-3.580256	0.236793	H	-2.745796	3.937992	1.375648
H	0.232557	0.99692	-1.786731	H	-0.460983	-0.475594	-1.945063
H	0.266849	-0.148442	2.919169	H	0.411291	-0.228711	2.808018
H	-0.066365	-1.253062	1.574846	H	0.704746	0.934993	1.51641
H	-4.073761	-0.371901	1.609503	H	4.403123	-0.984841	0.868176
H	1.181652	2.854196	0.425481	H	-0.43982	-3.047722	-0.464692
H	1.019564	5.124717	0.061086	H	-1.896164	-4.73725	-1.080714
H	-0.557142	5.596335	-0.604152	H	-2.658965	-4.464825	-2.660411
H	0.852051	5.560998	-1.651594	H	-3.578589	-4.185543	-1.189765
H	-1.025743	2.406105	-2.607894	H	-2.641219	-0.694119	-2.40569
H	-0.374906	3.936124	-3.236728	H	-4.006685	-1.661408	-1.853358
H	-1.777066	3.944943	-2.182664	H	-3.217435	-2.064116	-3.370767
H	-3.180959	-1.974549	-1.690299	H	3.629511	1.283381	-2.047031
H	-4.548544	-2.548328	0.991976	H	5.42159	1.030636	0.42692
H	-5.36892	-1.949327	-0.46335	H	5.849065	0.476817	-1.204293
H	-4.613739	-3.548166	-0.454112	H	5.594995	2.196104	-0.879244
H	-2.084309	-3.758084	-0.319353	H	3.270983	3.052926	-0.311466
H	-1.054269	-2.337018	-0.473183	H	1.863908	2.00374	-0.466052
H	-1.950671	-2.636498	1.033573	H	2.982945	1.805448	0.900345
H	3.560799	2.561523	2.014712	H	-2.490256	-2.396395	3.239658
H	4.08848	0.874468	1.80072	H	-0.751139	-2.051727	3.287968
H	3.401035	1.779395	0.42063	H	-1.926178	-0.793309	3.768025

2*S*,5*R*,8*R*,10*R*-**2S**				2*S*,5*R*,8*R*,10*R*-**2T**			
C	4.269114	-1.635155	-1.443896	C	3.980853	-2.167156	-1.501544
C	3.320547	-1.289992	-2.416692	C	2.924536	-1.958683	-2.399247
C	2.235265	-0.469492	-2.143102	C	1.921781	-1.027342	-2.1686
C	2.102776	0.025325	-0.843723	C	1.98918	-0.27449	-0.994311
C	3.036387	-0.343301	0.140095	C	3.029306	-0.50464	-0.077815
C	4.127025	-1.157936	-0.152951	C	4.034783	-1.434542	-0.328956
N	1.155313	0.893532	-0.341352	N	1.156532	0.741271	-0.569352
C	1.253091	0.928119	1.114976	C	1.442465	1.045354	0.829843
C	2.61768	0.248331	1.397583	C	2.806659	0.350206	1.074262
C	-0.137198	1.099646	-0.970031	C	-0.199157	0.897799	-1.058377
N	-1.114853	0.21701	-0.329876	N	-1.119182	0.19739	-0.155202
C	-1.202447	0.407982	1.104777	C	-1.019101	0.643151	1.220094
C	0.144833	0.071747	1.741949	C	0.39766	0.38018	1.732113
O	3.157651	0.232048	2.483224	O	3.486975	0.514178	2.064457
O	1.229146	2.278058	1.498648	O	1.517753	2.441561	0.947753
C	-1.610712	-0.846814	-1.002227	C	-1.797438	-0.885293	-0.593096

C	-2.368941	-1.871402	-0.181367	C	-2.672667	-1.625475	0.397626
N	-2.940372	-1.277913	1.013826	N	-2.810149	-0.937935	1.667301
C	-2.345958	-0.331398	1.762122	C	-2.039604	0.044678	2.158245
C	-0.552678	2.548504	-0.918174	C	-0.562692	2.353321	-1.216113
O	-1.44719	-1.00845	-2.203616	O	-1.746718	-1.270526	-1.754535
O	-2.699176	-0.028987	2.890146	O	-2.133139	0.473637	3.298155
C	-1.563078	3.06698	-1.621672	C	-1.620638	2.798586	-1.899867
C	-1.90892	4.520396	-1.504274	C	-1.902464	4.267528	-1.995253
C	-2.433322	2.278288	-2.551237	C	-2.60813	1.915327	-2.598347
C	-1.471247	-3.093095	0.145168	C	-2.199078	-3.083451	0.613855
C	-0.941143	-3.775828	-1.110104	C	-0.752488	-3.153981	1.08418
C	-2.23801	-4.091451	1.006835	C	-2.435736	-3.964324	-0.60632
H	-1.418618	1.469363	1.274235	H	-1.210874	1.723116	1.230569
H	-3.193339	-2.211204	-0.814186	H	-3.660013	-1.662037	-0.075066
C	1.095753	2.55578	2.88657	C	1.559042	2.978159	2.263417
H	5.099083	-2.279037	-1.705507	H	4.7398	-2.906005	-1.725176
H	3.434902	-1.676855	-3.423078	H	2.884686	-2.546176	-3.309623
H	1.525539	-0.230118	-2.923899	H	1.121324	-0.901775	-2.885619
H	4.831811	-1.417075	0.628646	H	4.826368	-1.582193	0.396529
H	-0.070342	0.758332	-2.001854	H	-0.278362	0.378977	-2.011895
H	0.113028	0.214031	2.822464	H	0.5017	0.719943	2.762456
H	0.379604	-0.980744	1.558279	H	0.581557	-0.697717	1.722607
H	-3.710464	-1.760654	1.454168	H	-3.483695	-1.31858	2.318191
H	0.029736	3.179926	-0.258727	H	0.101164	3.056823	-0.729264
H	-1.254657	5.040668	-0.802975	H	-1.165047	4.861151	-1.4527
H	-2.944825	4.641966	-1.169165	H	-2.895758	4.494254	-1.592579
H	-1.836752	5.012289	-2.480352	H	-1.908981	4.589833	-3.04213
H	-2.084078	1.259527	-2.720644	H	-2.305191	0.869127	-2.644729
H	-2.508127	2.787151	-3.517714	H	-2.779399	2.275556	-3.617786
H	-3.451696	2.222084	-2.150007	H	-3.575928	1.961254	-2.085734
H	-0.618029	-2.724103	0.728165	H	-2.837063	-3.461405	1.422633
H	-1.767291	-4.128528	-1.73543	H	-0.066215	-2.788172	0.312735
H	-0.322583	-3.111519	-1.713655	H	-0.596932	-2.575486	1.998324
H	-0.340482	-4.643288	-0.826379	H	-0.477313	-4.187982	1.303372
H	-1.611795	-4.96258	1.211459	H	-2.224685	-5.00622	-0.352682
H	-2.534455	-3.668736	1.969026	H	-3.473307	-3.902049	-0.945778
H	-3.137374	-4.439955	0.488146	H	-1.79042	-3.670297	-1.435192
H	1.327289	3.61469	2.997933	H	1.835409	4.025515	2.145103
H	0.072278	2.384083	3.236583	H	0.579757	2.928881	2.751334
H	1.794666	1.96913	3.485324	H	2.305607	2.47631	2.881495

2*S*,5*R*,8*R*,10*R*-**2U**			
C	3.980934	-2.166813	-1.501401
C	2.924529	-1.958628	-2.399063
C	1.921699	-1.027341	-2.168522

C	1.989106	-0.274254	-0.994385
C	3.02933	-0.504109	-0.077922
C	4.034881	-1.433957	-0.328963
N	1.156367	0.741497	-0.56957
C	1.442349	1.045854	0.829561
C	2.806655	0.350929	1.07401
C	-0.199389	0.897718	-1.058488
N	-1.119207	0.197176	-0.155203
C	-1.019156	0.64307	1.22005
C	0.39773	0.380618	1.731991
O	3.487004	0.515201	2.064132
O	1.517546	2.44209	0.947204
C	-1.797252	-0.885686	-0.592997
C	-2.672218	-1.626026	0.397833
N	-2.809849	-0.93838	1.667439
C	-2.039364	0.044303	2.158342
C	-0.563252	2.353153	-1.216263
O	-1.746534	-1.270961	-1.75442
O	-2.132781	0.473163	3.298298
C	-1.621472	2.798155	-1.899762
C	-1.90365	4.26703	-1.995132
C	-2.608972	1.914656	-2.597944
C	-2.198198	-3.083839	0.614167
C	-0.751552	-3.153934	1.084391
C	-2.434679	-3.964879	-0.605924
H	-1.211327	1.722966	1.23047
H	-3.659575	-1.662916	-0.074809
C	1.558516	2.979	2.262741
H	4.739941	-2.905629	-1.724938
H	2.884663	-2.546304	-3.309321
H	1.121168	-0.902003	-2.885496
H	4.826545	-1.581383	0.396482
H	-0.278564	0.378839	-2.011977
H	0.501739	0.720532	2.762285
H	0.581924	-0.697231	1.722635
H	-3.483138	-1.319261	2.318458
H	0.100619	3.056824	-0.729677
H	-1.166106	4.860853	-1.452974
H	-2.896798	4.493561	-1.591981
H	-1.91076	4.589265	-3.042026
H	-2.305723	0.868556	-2.644552
H	-2.780715	2.274932	-3.617287
H	-3.576594	1.960231	-2.084973
H	-2.836027	-3.461911	1.423013
H	-0.065431	-2.7881	0.312823

H	-0.596049	-2.575247	1.998424
H	-0.476118	-4.187832	1.303739
H	-2.223291	-5.00669	-0.352219
H	-3.472292	-3.902943	-0.945313
H	-1.789513	-3.67072	-1.434868
H	1.834207	4.026505	2.144169
H	0.579284	2.929236	2.750709
H	2.305412	2.477759	2.88092

Key transitions, oscillator strengths, and rotatory strengths in the ECD of conformers 2*S*,5*R*,8*R*,10*R*-**2Q** with cam-b3lyp function applying def2tzvpp basis set in methanol solvent.

Species	Excited State	$\Delta E(eV)^a$	$\lambda(nm)^b$	f^c	R_{vel}^d
2*S*,5*R*,8*R*,10*R*-**2Q**	104 -> 107	3.6897	336.03	0.0783	-81.0027
	98 -> 107	3.9232	316.03	0.0088	67.2601
	102 -> 107	5.1335	241.52	0.0394	-11.7459
	98 -> 107	5.6173	220.72	0.0592	8.0938
	102 -> 107	5.6603	219.04	0.664	48.7233
	99 -> 108	5.8675	211.31	0.0009	-7.4216
	99 -> 109	5.902	210.07	0.001	-10.2424
	97 -> 107	6.0048	206.47	0.0019	10.6335
	97 -> 107	6.1387	201.97	0.001	-3.3008
	97 -> 107	6.2133	199.55	0.0068	10.5978
	105 -> 109	6.2913	197.07	0.1044	61.8223
	98 -> 107	6.4549	192.08	0.0034	3.3763
	97 -> 107	6.6671	185.96	0.0552	3.7931
	101 -> 109	6.6759	185.72	0.3209	34.3924
	104 -> 108	6.736	184.06	0.0458	-41.8299
	91 -> 107	6.8092	182.08	0.0249	2.1246
	97 -> 107	6.8118	182.01	0.0955	-9.9517
	97 -> 107	6.9145	179.31	0.0762	-5.9539
	90 -> 107	6.9738	177.79	0.1206	127.549
	100 -> 109	7.0248	176.49	0.0241	-20.0374
	99 -> 109	7.0841	175.02	0.0706	56.7961
	97 -> 107	7.1072	174.45	0.0427	17.8326
	90 -> 107	7.1267	173.97	0.0644	22.5748
	99 -> 109	7.1759	172.78	0.0502	-127.5026
	100 -> 109	7.2286	171.52	0.2363	19.9014
	100 -> 108	7.3394	168.93	0.0126	9.1669
	102 -> 110	7.3705	168.22	0.0067	-4.7516
	86 -> 107	7.3909	167.75	0.0096	6.4454
	99 -> 109	7.4584	166.24	0.135	-101.5766
	86 -> 107	7.5456	164.31	0.0119	-2.8817
	86 -> 107	7.5482	164.26	0.0977	-82.8472
	86 -> 107	7.5837	163.49	0.0997	7.6977

	99 -> 110	7.6178	162.76	0.0478	20.9224
	101 -> 108	7.7136	160.73	0.0286	-20.6678
	98 -> 109	7.7517	159.94	0.0024	-0.9398
	101 -> 108	7.8042	158.87	0.0005	3.6099
	86 -> 107	7.8198	158.55	0.0077	33.7572
	86 -> 107	7.8265	158.42	0.0306	8.1684
	101 -> 110	7.8817	157.31	0.0012	0.0803
	99 -> 109	7.9184	156.58	0.04	-47.9497
	101 -> 110	7.9406	156.14	0.0101	-10.5431
	99 -> 109	8.0104	154.78	0.0275	37.3779
	97 -> 108	8.0233	154.53	0.0469	-34.4391
	99 -> 108	8.0371	154.27	0.0245	-13.4474
	99 -> 108	8.1312	152.48	0.0224	-19.9433
	81 -> 107	8.151	152.11	0.0076	5.0395
	81 -> 107	8.1595	151.95	0.0055	-11.3085
	95 -> 109	8.2074	151.06	0.0137	-33.67
	100 -> 109	8.2176	150.88	0.0041	-1.2146
	99 -> 109	8.246	150.36	0.0063	-22.8385

[a]Excitation energy. [b]Wavelength. [c]Oscillator strength. [d]Rotatory strength in velocity form (10^{-40}cgs.).

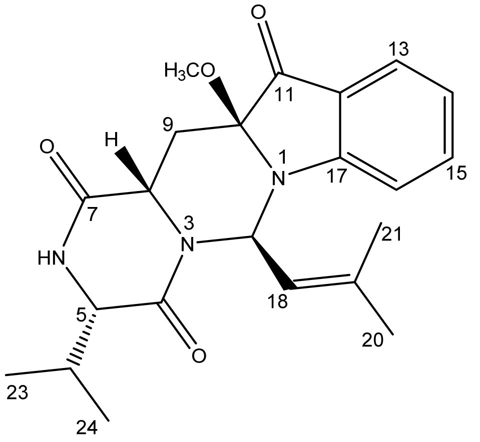

2*R*,5*S*,8*S*,10*S*-**2**

Optimized geometries of predominant conformers for compound 2*R*,5*S*,8*S*,10*S*-**2** with PBE0 function applying 6-311G(d,p) basis set for C, N, and O atoms and 6-31G(d,p) basis set for H atoms in methanol solvent.

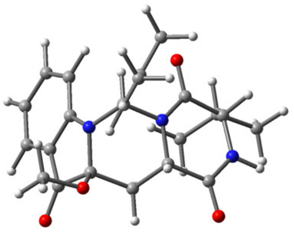

2*R*, 5*S*, 8*S*, 10*S*-**2A**

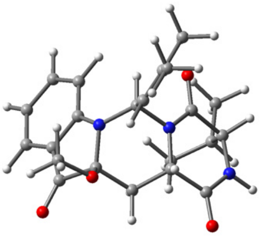

2*R*, 5*S*, 8*S*, 10*S*-**2B**

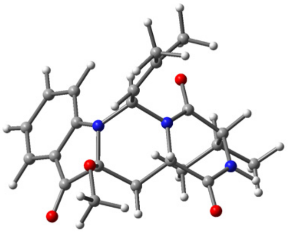

2*R*, 5*S*, 8*S*, 10*S*-**2C**

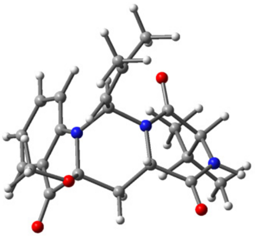

2*R*, 5*S*, 8*S*, 10*S*-**2D**

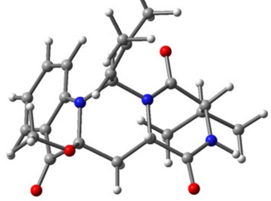

2*R*, 5*S*, 8*S*, 10*S*-**2E**

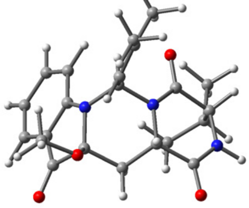

2*R*, 5*S*, 8*S*, 10*S*-**2F**

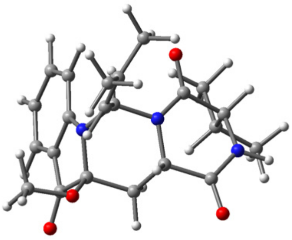

2*R*, 5*S*, 8*S*, 10*S*-**2G**

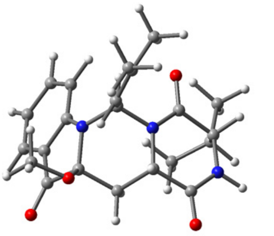

2*R*, 5*S*, 8*S*, 10*S*-**2H**

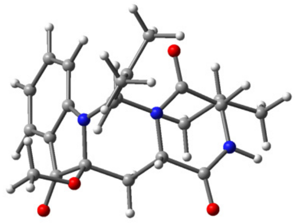

2*R*, 5*S*, 8*S*, 10*S*-**2I**

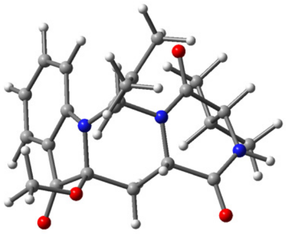

2*R*, 5*S*, 8*S*, 10*S*-**2J**

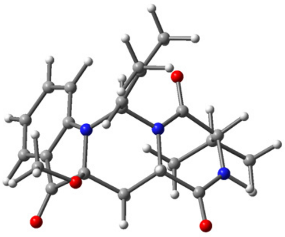

2*R*, 5*S*, 8*S*, 10*S*-**2K**

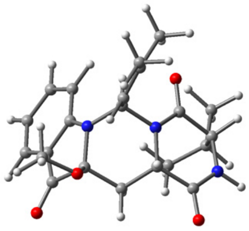

2*R*, 5*S*, 8*S*, 10*S*-**2L**

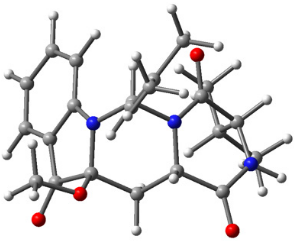

2*R*, 5*S*, 8*S*, 10*S*-**2M**

Important thermodynamic parameters (a.u.) and Boltzmann distributions of the optimized compound 2*R*,5*S*,8*S*,10*S*-**2** with PBE0 function applying 6-311G(d,p) basis set for C, N, and O atoms and 6-31G(d,p) basis set for H atoms in methanol solvent.

Conformations	E+ZPE	G	%
2*R*,5*S*,8*S*,10*S*-**2A**	-1318.202184	-1318.259972	16.80
2*R*,5*S*,8*S*,10*S*-**2B**	-1318.200720	-1318.259145	7.00
2*R*,5*S*,8*S*,10*S*-**2C**	-1318.196430	-1318.254262	0.04
2*R*,5*S*,8*S*,10*S*-**2D**	-1318.199226	-1318.257297	0.99
2*R*,5*S*,8*S*,10*S*-**2E**	-1318.202184	-1318.259974	16.84
2*R*,5*S*,8*S*,10*S*-**2F**	-1318.200721	-1318.259151	7.04

2*R*,5*S*,8*S*,10*S*-**2G**	-1318.199223	-1318.257275	0.97
2*R*,5*S*,8*S*,10*S*-**2H**	-1318.200722	-1318.259145	7.00
2*R*,5*S*,8*S*,10*S*-**2I**	-1318.202186	-1318.259988	17.09
2*R*,5*S*,8*S*,10*S*-**2J**	-1318.199227	-1318.257296	0.99
2*R*,5*S*,8*S*,10*S*-**2K**	-1318.202187	-1318.259992	17.16
2*R*,5*S*,8*S*,10*S*-**2L**	-1318.200724	-1318.259151	7.04
2*R*,5*S*,8*S*,10*S*-**2M**	-1318.199236	-1318.257346	1.04

E+ZPE, G: total energy with zero-point energy (ZPE) and Gibbs free energy in methanol solution. %: Boltzmann distributions, using the relative Gibbs free energies as weighting factors.

Optimized Z-matrixes (Å) of compound 2*R*,5*S*,8*S*,10*S*-**2** with PBE0 function applying 6-311G(d,p) basis set for C, N, and O atoms and 6-31G(d,p) basis set for H atoms in methanol solvent.

2*R*,5*S*,8*S*,10*S*-**2A**				2*R*,5*S*,8*S*,10*S*-**2B**			
C	2.799403	-3.269062	1.786489	C	3.063265	-2.978681	1.730086
C	2.10766	-2.399647	2.64114	C	2.143202	-2.327673	2.563292
C	1.591337	-1.185572	2.210683	C	1.48014	-1.170041	2.180686
C	1.784376	-0.831073	0.873997	C	1.758883	-0.645342	0.916966
C	2.446339	-1.717809	0.006789	C	2.653722	-1.31717	0.065786
C	2.97063	-2.927084	0.456992	C	3.321498	-2.470769	0.469369
N	1.418154	0.337227	0.228242	N	1.274254	0.513631	0.336105
C	1.541077	0.128091	-1.222268	C	1.585828	0.481561	-1.100864
C	2.433581	-1.136291	-1.322287	C	2.689004	-0.60448	-1.197526
C	0.331706	1.164802	0.716688	C	0.016859	1.101862	0.756852
N	-0.927869	0.696657	0.128235	N	-1.072215	0.497509	-0.017899
C	-0.925169	0.699351	-1.322608	C	-0.917497	0.649807	-1.45158
C	0.183836	-0.221967	-1.834885	C	0.382602	-0.018014	-1.903034
O	2.952356	-1.510129	-2.350683	O	3.375529	-0.776774	-2.180092
O	2.041226	1.232865	-1.900598	O	1.956998	1.717331	-1.616321
C	-1.876745	0.161598	0.923487	C	-2.026657	-0.214081	0.618519
C	-3.111831	-0.417348	0.268539	C	-3.065283	-0.921829	-0.224874
N	-3.201529	-0.147069	-1.151179	N	-3.055878	-0.502018	-1.612708
C	-2.240466	0.320369	-1.962794	C	-2.078718	0.138667	-2.272854
C	0.572616	2.623687	0.437256	C	0.035802	2.600675	0.631457
O	-1.777806	0.11317	2.143109	O	-2.072519	-0.314629	1.838428
O	-2.381047	0.449976	-3.168898	O	-2.094836	0.334653	-3.47832
C	0.07523	3.625619	1.168369	C	-0.63359	3.439615	1.42796
C	0.376348	5.049637	0.810804	C	-0.536227	4.921007	1.220373
C	-0.810561	3.449345	2.363299	C	-1.521993	3.020665	2.559054
C	-3.197554	-1.923747	0.598164	C	-2.935445	-2.461565	-0.115696
C	-4.51149	-2.514696	0.102931	C	-1.553092	-2.952675	-0.524672
C	-2.002118	-2.698665	0.059255	C	-3.332513	-2.986883	1.25799
H	-0.720727	1.723452	-1.654809	H	-0.859063	1.722742	-1.669574
H	-3.95533	0.078263	0.761373	H	-4.028837	-0.635619	0.208856
C	3.350587	1.619679	-1.50377	C	3.124126	2.27224	-1.023815
H	3.184093	-4.206149	2.168397	H	3.555768	-3.879845	2.072578

H	1.967146	-2.682662	3.678278	H	1.937525	-2.74127	3.544137
H	1.055491	-0.548122	2.901378	H	0.770498	-0.704423	2.851753
H	3.482735	-3.584296	-0.236157	H	4.011327	-2.959906	-0.208515
H	0.233598	0.997185	1.786134	H	-0.164075	0.813625	1.788657
H	0.266088	-0.149844	-2.919364	H	0.564419	0.18129	-2.959028
H	-0.068238	-1.253619	-1.574627	H	0.291001	-1.098362	-1.765034
H	-4.074623	-0.367929	-1.609099	H	-3.815814	-0.83196	-2.192206
H	1.183587	2.852694	-0.427462	H	0.636617	3.005822	-0.174189
H	1.025705	5.123397	-0.062907	H	0.129261	5.17548	0.394034
H	-0.549363	5.597531	0.604428	H	-1.524826	5.345698	1.015454
H	0.861198	5.560094	1.649954	H	-0.167902	5.41055	2.128418
H	-1.76913	3.949127	2.18548	H	-1.620528	1.940651	2.667164
H	-1.020793	2.408304	2.608755	H	-1.154449	3.441224	3.501257
H	-0.365254	3.936477	3.237214	H	-2.525765	3.432293	2.40777
H	-3.183149	-1.9702	1.691338	H	-3.661665	-2.849268	-0.841168
H	-4.551447	-2.543834	-0.990601	H	-0.781965	-2.594874	0.166315
H	-5.37116	-1.943327	0.464475	H	-1.294758	-2.631807	-1.536928
H	-4.617589	-3.542927	0.455968	H	-1.522722	-4.044377	-0.512299
H	-2.088467	-3.755507	0.321118	H	-3.367543	-4.078991	1.236525
H	-1.056906	-2.335474	0.474271	H	-4.320028	-2.621854	1.552984
H	-1.953722	-2.634656	-1.032305	H	-2.616048	-2.677319	2.020311
H	3.56316	2.556679	-2.016522	H	3.230176	3.27524	-1.434652
H	4.088908	0.869218	-1.801047	H	4.010781	1.682957	-1.275501
H	3.402316	1.776061	-0.4218	H	3.020615	2.332423	0.063972

2*R*,5*S*,8*S*,10*S*-**2C**				2*R*,5*S*,8*S*,10*S*-**2D**			
C	-3.85213	-2.431225	-1.65716	C	-3.240784	-2.912882	-1.565358
C	-2.969084	-1.857558	-2.582658	C	-2.48125	-2.17133	-2.481013
C	-2.08171	-0.84709	-2.240231	C	-1.792296	-1.022147	-2.120126
C	-2.087777	-0.39429	-0.919131	C	-1.874971	-0.600734	-0.791274
C	-2.951362	-0.988147	0.017169	C	-2.608191	-1.362088	0.135753
C	-3.844314	-1.993513	-0.344593	C	-3.305824	-2.506106	-0.244849
N	-1.350648	0.624248	-0.351051	N	-1.331956	0.530803	-0.208174
C	-1.494627	0.578703	1.100045	C	-1.425379	0.396039	1.254172
C	-2.695055	-0.380241	1.31013	C	-2.464324	-0.740614	1.438306
C	-0.112695	1.116239	-0.922034	C	-0.167951	1.192679	-0.772766
N	1.018589	0.435637	-0.28152	N	1.03503	0.590642	-0.196027
C	1.026379	0.56718	1.162704	C	1.079668	0.676546	1.251237
C	-0.250685	-0.054269	1.730281	C	-0.099563	-0.089455	1.849944
O	-3.247452	-0.555755	2.375231	O	-2.982062	-1.007776	2.500118
O	-1.766781	1.886708	1.532323	O	-1.761305	1.582627	1.895497
C	1.78336	-0.414363	-0.998075	C	1.838975	-0.181767	-0.961063
C	2.889073	-1.158787	-0.279039	C	2.860915	-1.022301	-0.222757
N	3.065352	-0.757157	1.101101	N	3.238296	-0.409335	1.037638
C	2.237331	-0.015171	1.851887	C	2.398074	0.247992	1.857614

C	-0.005838	2.615899	-0.801965	C	-0.216986	2.682299	-0.561504
O	1.633818	-0.597773	-2.199209	O	1.738179	-0.246507	-2.178411
O	2.416352	0.206429	3.039522	O	2.659704	0.534404	3.014256
C	0.868727	3.365364	-1.478977	C	0.447401	3.570206	-1.306747
C	0.906206	4.85167	-1.290628	C	0.33751	5.037526	-1.023051
C	1.877554	2.82097	-2.443103	C	1.348912	3.210467	-2.447464
C	2.668802	-2.678582	-0.435526	C	2.343705	-2.471951	-0.034856
C	3.85204	-3.460918	0.120267	C	1.998893	-3.143648	-1.358796
C	1.358475	-3.135687	0.191627	C	3.372443	-3.299788	0.728501
H	1.041884	1.63832	1.396313	H	0.978758	1.734981	1.515945
H	3.802704	-0.898929	-0.825622	H	3.74745	-1.051738	-0.861562
C	-1.729342	2.126147	2.933156	C	-3.031433	2.106533	1.529759
H	-4.525976	-3.217777	-1.972049	H	-3.761353	-3.803328	-1.894074
H	-2.975805	-2.214224	-3.606532	H	-2.426721	-2.50471	-3.511238
H	-1.415405	-0.433452	-2.985608	H	-1.211752	-0.48402	-2.857665
H	-4.501299	-2.425179	0.401591	H	-3.86825	-3.065455	0.493701
H	-0.077337	0.812347	-1.966678	H	-0.130128	0.963464	-1.835227
H	-0.274041	0.04428	2.815561	H	-0.141166	0.047648	2.930852
H	-0.25531	-1.123411	1.499269	H	0.018261	-1.156383	1.641331
H	3.87912	-1.110705	1.584443	H	4.119088	-0.683714	1.448295
H	-0.699394	3.081628	-0.112777	H	-0.827999	3.032359	0.261305
H	0.166226	5.190055	-0.5637	H	-0.329099	5.242648	-0.183941
H	1.897991	5.170141	-0.95135	H	1.322605	5.459456	-0.796526
H	0.722126	5.363819	-2.241255	H	-0.035891	5.57069	-1.904015
H	1.734469	1.766418	-2.679991	H	1.402284	2.140125	-2.647728
H	1.855044	3.394473	-3.375217	H	1.026035	3.724558	-3.358936
H	2.886445	2.937092	-2.03067	H	2.365226	3.561765	-2.237442
H	2.617767	-2.84229	-1.516184	H	1.4321	-2.413826	0.573004
H	3.911916	-3.37764	1.2101	H	2.875755	-3.182417	-2.012606
H	4.798838	-3.116184	-0.304964	H	1.203066	-2.623696	-1.892632
H	3.743796	-4.522126	-0.114839	H	1.673372	-4.169779	-1.171945
H	1.230897	-4.212149	0.057739	H	3.012216	-4.32421	0.845781
H	0.494206	-2.644947	-0.26581	H	3.564659	-2.904349	1.728095
H	1.347481	-2.93538	1.267625	H	4.320457	-3.338442	0.181664
H	-2.179363	3.108279	3.075533	H	-3.103346	3.09021	1.991629
H	-0.701866	2.155341	3.31138	H	-3.840177	1.471567	1.903064
H	-2.305628	1.382242	3.486167	H	-3.116421	2.206983	0.44316

2*R*,5*S*,8*S*,10*S*-**2E**				2*R*,5*S*,8*S*,10*S*-**2F**			
C	2.800136	-3.268423	1.786154	C	3.063379	-2.979207	1.731132
C	2.10794	-2.399476	2.640916	C	2.144087	-2.327107	2.56434
C	1.591366	-1.185421	2.210709	C	1.481124	-1.16964	2.181075
C	1.784598	-0.830462	0.874175	C	1.759144	-0.646242	0.916651
C	2.447027	-1.716728	0.00685	C	2.653258	-1.31913	0.065536
C	2.971582	-2.925981	0.456805	C	3.320946	-2.472553	0.469777

N	1.418178	0.337948	0.228709	N	1.274532	0.512326	0.335033
C	1.541472	0.12927	-1.221841	C	1.58555	0.47915	-1.102006
C	2.434439	-1.134779	-1.32204	C	2.688114	-0.607534	-1.198395
C	0.331216	1.16488	0.717122	C	0.017732	1.101682	0.755989
N	-0.927997	0.696152	0.128383	N	-1.072099	0.49801	-0.018245
C	-0.92512	0.699356	-1.32246	C	-0.917578	0.649246	-1.45206
C	0.184508	-0.221159	-1.83487	C	0.381724	-0.020189	-1.903358
O	2.953609	-1.508112	-2.350419	O	3.373976	-0.780977	-2.181225
O	2.041381	1.234404	-1.899769	O	1.957198	1.714407	-1.618388
C	-1.876779	0.160519	0.923341	C	-2.027515	-0.211658	0.61885
C	-3.111438	-0.418945	0.268042	C	-3.067077	-0.918855	-0.223859
N	-3.200995	-0.148494	-1.151652	N	-3.057389	-0.500103	-1.612006
C	-2.240059	0.319702	-1.962968	C	-2.079526	0.138849	-2.272775
C	0.571394	2.623947	0.437964	C	0.037909	2.600431	0.630158
O	-1.778041	0.111884	2.142974	O	-2.073462	-0.311065	1.838847
O	-2.380462	0.449459	-3.169083	O	-2.095556	0.333929	-3.478391
C	0.072593	3.625497	1.168639	C	-0.629241	3.440332	1.427531
C	0.37302	5.049743	0.811413	C	-0.530623	4.921555	1.219299
C	-0.814264	3.448503	2.362669	C	-1.516175	3.022768	2.560293
C	-3.196392	-1.925451	0.597362	C	-2.938909	-2.458653	-0.113567
C	-4.509936	-2.517013	0.101838	C	-1.557121	-2.951527	-0.522337
C	-2.000443	-2.699581	0.058449	C	-3.33641	-2.982559	1.260533
H	-0.721273	1.7237	-1.654274	H	-0.858061	1.721975	-1.670786
H	-3.955297	0.07613	0.760793	H	-4.030247	-0.63125	0.209791
C	3.350502	1.621604	-1.502543	C	3.124835	2.269066	-1.026661
H	3.185004	-4.205521	2.167856	H	3.555844	-3.880187	2.07416
H	1.967272	-2.682851	3.677934	H	1.938985	-2.739672	3.545741
H	1.055186	-0.548331	2.901476	H	0.772248	-0.703056	2.852282
H	3.484049	-3.582828	-0.236423	H	4.010222	-2.96251	-0.208078
H	0.233028	0.997044	1.786535	H	-0.16315	0.813861	1.787913
H	0.266949	-0.148579	-2.919303	H	0.563376	0.177988	-2.959593
H	-0.067133	-1.253036	-1.575074	H	0.289231	-1.100326	-1.764284
H	-4.073812	-0.369939	-1.609816	H	-3.817798	-0.829568	-2.19115
H	1.182984	2.853417	-0.42619	H	0.637827	3.004723	-0.176597
H	1.022838	5.124012	-0.061912	H	0.133982	5.175065	0.391955
H	-0.552913	5.597082	0.604564	H	-1.51907	5.347187	1.015597
H	0.857067	5.560398	1.650907	H	-0.160547	5.411029	2.126667
H	-1.773398	3.946728	2.183501	H	-1.616423	1.942913	2.668385
H	-1.023214	2.407268	2.608404	H	-1.146049	3.442536	3.501841
H	-0.370698	3.936756	3.236837	H	-2.519503	3.436204	2.411028
H	-3.182109	-1.972112	1.690529	H	-3.665628	-2.846091	-0.838683
H	-4.549763	-2.545936	-0.991704	H	-0.785553	-2.594078	0.168337
H	-5.369963	-1.946204	0.463421	H	-1.298523	-2.631595	-1.534823
H	-4.615507	-3.545385	0.454629	H	-1.527911	-4.043253	-0.509246
H	-2.086159	-3.756505	0.320194	H	-3.372599	-4.074644	1.239861

H	-1.055474	-2.335854	0.473557	H	-4.323512	-2.616266	1.555339
H	-1.952006	-2.635421	-1.033101	H	-2.619563	-2.673209	2.022585
H	3.563012	2.558581	-2.015365	H	3.231343	3.271703	-1.438264
H	4.089133	0.871302	-1.799454	H	4.011067	1.679082	-1.2782
H	3.401825	1.77815	-0.420578	H	3.021707	2.330106	0.061114

2*R*,5*S*,8*S*,10*S*-**2G**				2*R*,5*S*,8*S*,10*S*-**2H**			
C	3.239342	-2.913306	1.565381	C	-3.061878	-2.980402	-1.731541
C	2.479955	-2.171574	2.481004	C	-2.142812	-2.327793	-2.564597
C	1.791481	-1.0221	2.120122	C	-1.480383	-1.170083	-2.181138
C	1.8745	-0.600604	0.791324	C	-1.758731	-0.646963	-0.916671
C	2.607611	-1.362089	-0.135665	C	-2.652608	-1.320357	-0.065705
C	3.304758	-2.506406	0.244926	C	-3.319758	-2.474021	-0.470137
N	1.331894	0.531194	0.208272	N	-1.274664	0.511731	-0.334858
C	1.425322	0.396364	-1.254094	C	-1.585738	0.478207	1.102158
C	2.464119	-0.740425	-1.438179	C	-2.687849	-0.608955	1.198326
C	0.167787	1.193	0.772782	C	-0.018139	1.10175	-0.755692
N	-1.035125	0.590732	0.196139	N	1.071956	0.498486	0.018499
C	-1.079821	0.676634	-1.251128	C	0.917301	0.649319	1.452343
C	0.09951	-0.089177	-1.84987	C	-0.381729	-0.020769	1.903454
O	2.98202	-1.007544	-2.499917	O	-3.3737	-0.782828	2.181087
O	1.761373	1.582859	-1.895492	O	-1.957916	1.713227	1.618725
C	-1.838756	-0.182017	0.96115	C	2.027866	-0.2104	-0.618732
C	-2.860303	-1.022964	0.222761	C	3.067659	-0.917396	0.223852
N	-3.238093	-0.409931	-1.037472	N	3.057658	-0.499061	1.612123
C	-2.398237	0.247922	-1.857406	C	2.079423	0.139209	2.273001
C	0.216702	2.682588	0.561318	C	-0.03903	2.600467	-0.629676
O	-1.737957	-0.246761	2.178499	O	2.074033	-0.309328	-1.838758
O	-2.660169	0.534623	-3.013901	O	2.095248	0.333915	3.478679
C	-0.447528	3.570604	1.306572	C	0.627221	3.440835	-1.42731
C	-0.337603	5.03789	1.022693	C	0.527876	4.921976	-1.218847
C	-1.348796	3.211109	2.447556	C	1.513741	3.023915	-2.560637
C	-2.342367	-2.472318	0.034516	C	2.940135	-2.457212	0.113077
C	-1.996919	-3.144076	1.358256	C	1.558536	-2.950788	0.521641
C	-3.370836	-3.30051	-0.728819	C	3.337914	-2.980523	-1.26117
H	-0.979085	1.735093	-1.515822	H	0.857325	1.721969	1.671333
H	-3.746787	-1.05301	0.86161	H	4.030749	-0.629258	-0.20962
C	3.031475	2.106751	-1.529623	C	-3.125709	2.267538	1.026985
H	3.759514	-3.803993	1.894073	H	-3.55392	-3.881556	-2.074719
H	2.425151	-2.505046	3.511184	H	-1.937452	-2.740145	-3.546033
H	1.211005	-0.483821	2.857606	H	-0.771656	-0.703109	-2.852232
H	3.867096	-3.065897	-0.493582	H	-4.008864	-2.964367	0.207611
H	0.130035	0.96394	1.83527	H	0.162918	0.814146	-1.787645
H	0.14111	0.047974	-2.930771	H	-0.563488	0.177107	2.959727
H	-0.018163	-1.15614	-1.641336	H	-0.288778	-1.100837	1.764156

H	-4.118938	-0.68441	-1.44795	H	3.818141	-0.828389	2.191248
H	0.827493	3.032523	-0.26171	H	-0.638735	3.004339	0.17745
H	0.329087	5.242908	0.183618	H	-0.13637	5.175009	-0.391068
H	-1.322668	5.459821	0.796051	H	1.5162	5.348161	-1.01571
H	0.035753	5.571145	1.903623	H	0.156954	5.411335	-2.125933
H	-2.364955	3.563131	2.238008	H	1.614745	1.944134	-2.668744
H	-1.402782	2.140748	2.647554	H	1.142679	3.443357	-3.501963
H	-1.025259	3.724801	3.359026	H	2.516846	3.438128	-2.412042
H	-1.430912	-2.413661	-0.573512	H	3.666994	-2.844571	0.838095
H	-2.873538	-3.183255	2.012363	H	0.786831	-2.593387	-0.168909
H	-1.201078	-2.623927	1.891881	H	1.299794	-2.631329	1.53424
H	-1.671084	-4.170063	1.171157	H	1.529759	-4.04252	0.508151
H	-3.010161	-4.324746	-0.846334	H	3.374585	-4.072598	-1.240826
H	-3.563383	-2.904981	-1.728312	H	4.324866	-2.613701	-1.555822
H	-4.318762	-3.339684	-0.181862	H	2.620964	-2.671263	-2.02316
H	3.103524	3.090344	-1.991651	H	-3.232709	3.270017	1.438845
H	3.840239	1.471666	-1.902683	H	-4.011725	1.677107	1.278236
H	3.11628	2.207376	-0.443027	H	-3.022454	2.328917	-0.060759

2*R*,5*S*,8*S*,10*S*-**2I**				2*R*,5*S*,8*S*,10*S*-**2J**			
C	-2.803347	-3.266373	-1.785946	C	-3.238903	-2.913993	-1.565027
C	-2.110558	-2.398004	-2.640815	C	-2.478909	-2.172902	-2.480668
C	-1.592864	-1.184399	-2.210686	C	-1.790579	-1.023242	-2.120107
C	-1.785558	-0.829279	-0.874115	C	-1.87444	-0.600853	-0.791648
C	-2.448596	-1.714999	-0.006686	C	-2.60805	-1.361761	0.135415
C	-2.974256	-2.923801	-0.456562	C	-3.305031	-2.506286	-0.244858
N	-1.418027	0.338788	-0.228693	N	-1.332194	0.531349	-0.209009
C	-1.541322	0.130248	1.221881	C	-1.426061	0.397154	1.253409
C	-2.435254	-1.133103	1.322213	C	-2.465104	-0.739393	1.437657
C	-0.330569	1.164942	-0.717285	C	-0.167852	1.192828	-0.773385
N	0.928409	0.695493	-0.128584	N	1.034843	0.590594	-0.196261
C	0.925576	0.698745	1.322255	C	1.079147	0.677031	1.250983
C	-0.184534	-0.221134	1.83473	C	-0.100462	-0.088366	1.8497
O	-2.954511	-1.506043	2.350694	O	-2.983525	-1.005861	2.499304
O	-2.040318	1.235787	1.899809	O	-1.762076	1.583958	1.89423
C	1.876716	0.15898	-0.923529	C	1.838462	-0.182663	-0.960782
C	3.111183	-0.420919	-0.268253	C	2.860061	-1.023122	-0.221902
N	3.201042	-0.150175	1.151367	N	3.237457	-0.409617	1.038223
C	2.240357	0.31847	1.962733	C	2.397346	0.248382	1.857777
C	-0.569749	2.624208	-0.438253	C	-0.2166	2.682497	-0.562358
O	1.777768	0.110059	-2.143132	O	1.737719	-0.248062	-2.178101
O	2.380896	0.448219	3.168826	O	2.658924	0.535311	3.014296
C	-0.069817	3.625342	-1.168722	C	0.448795	3.570054	-1.307116
C	-0.369166	5.049844	-0.811622	C	0.339118	5.037463	-1.023788
C	0.817374	3.447565	-2.362386	C	1.351273	3.209837	-2.446909

C	3.195487	-1.927527	-0.597265	C	2.342615	-2.472607	-0.033369
C	4.508881	-2.519498	-0.101818	C	1.998552	-3.145055	-1.35712
C	1.999314	-2.701074	-0.058023	C	3.370908	-3.299961	0.731125
H	0.722275	1.723211	1.654036	H	0.97851	1.735613	1.515231
H	3.955192	0.073691	-0.761219	H	3.746684	-1.05309	-0.860564
C	-3.349281	1.623815	1.502845	C	-3.032061	2.107845	1.527946
H	-3.189088	-4.203134	-2.167595	H	-3.758936	-3.804856	-1.893463
H	-1.970318	-2.681479	-3.677865	H	-2.423473	-2.507066	-3.510589
H	-1.056271	-0.54776	-2.901548	H	-1.209466	-0.485577	-2.857532
H	-3.487168	-3.580211	0.236752	H	-3.867779	-3.065324	0.493681
H	-0.232566	0.996909	-1.786686	H	-0.129864	0.963549	-1.835822
H	-0.266789	-0.14859	2.91918	H	-0.1423	0.049181	2.930541
H	0.066399	-1.253152	1.574803	H	0.017097	-1.155423	1.64157
H	4.073799	-0.37196	1.60948	H	4.118197	-0.683932	1.449028
H	-1.181544	2.854153	0.425627	H	-0.828205	3.032862	0.259874
H	-1.019478	5.124671	0.061286	H	-0.328413	5.24297	-0.185503
H	0.557146	5.596302	-0.604134	H	1.324094	5.459077	-0.796176
H	-0.852178	5.56101	-1.651401	H	-0.03306	5.570613	-1.90528
H	1.02551	2.406147	-2.608029	H	1.404337	2.139472	-2.647123
H	0.374554	3.936168	-3.23674	H	1.029611	3.724115	-3.358708
H	1.776856	3.94499	-2.182867	H	2.367537	3.560653	-2.235825
H	3.181024	-1.974409	-1.69042	H	1.430659	-2.414117	0.573938
H	4.548845	-2.548233	0.991725	H	2.875849	-3.184489	-2.010307
H	5.369069	-1.949071	-0.46362	H	1.203211	-2.625212	-1.891779
H	4.614022	-3.547974	-0.45443	H	1.672612	-4.170981	-1.169864
H	2.084587	-3.758092	-0.319529	H	3.011049	-4.324552	0.848036
H	1.054442	-2.337074	-0.473113	H	3.561775	-2.904378	1.730919
H	1.951023	-2.636639	1.033517	H	4.319575	-3.33816	0.185373
H	-3.560714	2.561455	2.0149	H	-3.103973	3.091739	1.989354
H	-4.088421	0.874425	1.800783	H	-3.840957	1.473128	1.901341
H	-3.400957	1.779438	0.420761	H	-3.116767	2.207804	0.441281

2*R*,5*S*,8*S*,10*S*-**2K**				2*R*,5*S*,8*S*,10*S*-**2L**			
C	2.803391	-3.266236	1.785547	C	3.061737	-2.980949	1.732015
C	2.110287	-2.398224	2.640523	C	2.143104	-2.327719	2.565065
C	1.59257	-1.184535	2.210657	C	1.480821	-1.170038	2.181271
C	1.785565	-0.828956	0.874252	C	1.758867	-0.647583	0.91646
C	2.448923	-1.714318	0.006705	C	2.652303	-1.321604	0.065516
C	2.974601	-2.92321	0.456318	C	3.319312	-2.475234	0.470283
N	1.418057	0.33929	0.229116	N	1.274904	0.510941	0.334265
C	1.541636	0.131151	-1.221505	C	1.585608	0.476774	-1.102804
C	2.435818	-1.132008	-1.322015	C	2.687309	-0.61081	-1.198858
C	0.330278	1.165036	0.717697	C	0.018764	1.101674	0.755261
N	-0.92847	0.695274	0.128757	N	-1.07182	0.498864	-0.018589
C	-0.925467	0.698947	-1.322082	C	-0.917308	0.64913	-1.45251

C	0.185028	-0.220402	-1.834679	C	0.381185	-0.022005	-1.90358
O	2.955366	-1.504555	-2.350491	O	3.372743	-0.785361	-2.181792
O	2.040512	1.236956	-1.899068	O	1.958105	1.711475	-1.619918
C	-1.876653	0.158156	0.923435	C	-2.028179	-0.209073	0.61902
C	-3.110777	-0.422114	0.267844	C	-3.06868	-0.915499	-0.223172
N	-3.20056	-0.151069	-1.151722	N	-3.058609	-0.497582	-1.611568
C	-2.240023	0.318343	-1.96282	C	-2.079952	0.139649	-2.272819
C	0.569014	2.624428	0.438902	C	0.040412	2.600349	0.628981
O	-1.777851	0.108969	2.14304	O	-2.074224	-0.307602	1.839083
O	-2.38051	0.448469	-3.168879	O	-2.095799	0.333959	-3.478561
C	0.068013	3.625269	1.169037	C	-0.624511	3.441273	1.427137
C	0.366858	5.04995	0.81223	C	-0.524462	4.922312	1.21829
C	-0.820007	3.44693	2.361997	C	-1.510139	3.025132	2.561453
C	-3.194452	-1.928841	0.596476	C	-2.942357	-2.455382	-0.111979
C	-4.507548	-2.521256	0.100767	C	-1.56127	-2.950188	-0.520781
C	-1.997895	-2.701731	0.057144	C	-3.340158	-2.977954	1.262542
H	-0.722502	1.723586	-1.653533	H	-0.856584	1.721664	-1.671863
H	-3.955072	0.071997	0.76082	H	-4.031464	-0.626486	0.210393
C	3.349356	1.625116	-1.501828	C	3.126188	2.265635	-1.028613
H	3.189134	-4.203081	2.166984	H	3.553689	-3.882048	2.075465
H	1.969807	-2.682058	3.677442	H	1.937981	-2.739546	3.546771
H	1.055709	-0.548193	2.901583	H	0.772463	-0.702554	2.8524
H	3.487759	-3.579338	-0.23708	H	4.008082	-2.966062	-0.207458
H	0.232199	0.996824	1.787066	H	-0.162215	0.814341	1.787299
H	0.26744	-0.147479	-2.919092	H	0.562829	0.175181	-2.960002
H	-0.065641	-1.252584	-1.575147	H	0.287638	-1.101938	-1.763629
H	-4.073173	-0.373078	-1.610002	H	-3.819462	-0.826477	-2.190451
H	1.181355	2.854708	-0.424498	H	0.639516	3.003716	-0.17885
H	1.01792	5.125168	-0.060087	H	0.139316	5.174787	0.389965
H	-0.559593	5.59585	0.603887	H	-1.512693	5.34902	1.015791
H	0.848788	5.561405	1.652452	H	-0.15259	5.411653	2.124996
H	-1.779697	3.943673	2.181688	H	-1.612116	1.94544	2.66956
H	-1.027595	2.405376	2.607536	H	-1.137568	3.444112	3.502389
H	-0.378229	3.935889	3.236679	H	-2.512995	3.440364	2.414049
H	-3.180064	-1.975983	1.689621	H	-3.669733	-2.842368	-0.836678
H	-4.547414	-2.549705	-0.992786	H	-0.789091	-2.593181	0.169437
H	-5.368012	-1.951301	0.462657	H	-1.302551	-2.631263	-1.533555
H	-4.612268	-3.549874	0.453091	H	-1.533358	-4.041938	-0.506947
H	-2.082679	-3.758835	0.318464	H	-3.377841	-4.070001	1.242507
H	-1.053203	-2.33736	0.472323	H	-4.326663	-2.610133	1.557443
H	-1.949601	-2.637085	-1.034382	H	-2.622655	-2.669151	2.024196
H	3.560672	2.562941	-2.013593	H	3.233558	3.267838	-1.441047
H	4.088672	0.875949	-1.799887	H	4.011941	1.674705	-1.279627
H	3.400866	1.780443	-0.419694	H	3.023074	2.327666	0.059107

2*R*,5*S*,8*S*,10*S*-**2M**			
C	3.233173	-2.919962	1.565161
C	2.475116	-2.176818	2.480753
C	1.789062	-1.025894	2.11992
C	1.873264	-0.604326	0.791221
C	2.604869	-1.367241	-0.135786
C	3.299613	-2.513038	0.24477
N	1.333084	0.528674	0.208301
C	1.426155	0.393708	-1.254073
C	2.462708	-0.745166	-1.438239
C	0.170321	1.19278	0.772857
N	-1.033838	0.592901	0.196287
C	-1.078462	0.678918	-1.250988
C	0.099327	-0.089241	-1.84974
O	2.980109	-1.013188	-2.500002
O	1.764467	1.579555	-1.89548
C	-1.838535	-0.178788	0.961266
C	-2.86198	-1.01745	0.222897
N	-3.238825	-0.403429	-1.037144
C	-2.397863	0.252933	-1.85713
C	0.222271	2.682246	0.561396
O	-1.737376	-0.244231	2.178548
O	-2.659489	0.540412	-3.013506
C	-0.440093	3.57158	1.306726
C	-0.327011	5.038667	1.023069
C	-1.34221	3.213839	2.447602
C	-2.347475	-2.467973	0.034217
C	-2.003844	-3.140928	1.357809
C	-3.378017	-3.293343	-0.729393
H	-0.975652	1.737172	-1.515683
H	-3.748362	-1.045681	0.861975
C	3.035532	2.101091	-1.529598
H	3.751468	-3.811748	1.893838
H	2.419397	-2.510368	3.510859
H	1.209387	-0.486632	2.857313
H	3.860868	-3.073613	-0.493737
H	0.132208	0.963895	1.835369
H	0.141159	0.047682	-2.930662
H	-0.020395	-1.155941	-1.641026
H	-4.120471	-0.675791	-1.447298
H	0.833808	3.030969	-0.2616
H	0.339765	5.24234	0.183737
H	-1.311218	5.46289	0.797002
H	0.047988	5.57087	1.903944
H	-2.357746	3.567439	2.237725

H	-1.397959	2.143605	2.647845
H	-1.018044	3.727185	3.359035
H	-1.435842	-2.411438	-0.573756
H	-2.880812	-3.178642	2.011537
H	-1.207182	-2.622521	1.891892
H	-1.67997	-4.167491	1.170447
H	-3.020296	-4.318656	-0.846513
H	-3.569037	-2.897493	-1.729042
H	-4.326278	-3.329618	-0.182801
H	3.109635	3.084307	-1.992105
H	3.843124	1.464224	-1.902161
H	3.120299	2.202095	-0.443037

Key transitions, oscillator strengths, and rotatory strengths in the ECD of conformers 2*R*,5*S*,8*S*,10*S*-**2K** with cam-b3lyp function applying def2tzvpp basis set in methanol solvent.

Species	Excited State	$\Delta E(eV)^a$	$\lambda(nm)^b$	f^c	R_{vel}^d
2*R*,5*S*,8*S*,10*S*-**2K**	104 -> 107	3.6898	336.02	0.0782	81.0465
	98 -> 107	3.9232	316.02	0.0088	-67.2873
	102 -> 107	5.1336	241.52	0.0394	11.7554
	98 -> 107	5.6172	220.72	0.059	-8.0761
	102 -> 107	5.6603	219.04	0.6641	-48.8242
	99 -> 108	5.8675	211.31	0.0009	7.4133
	99 -> 109	5.902	210.07	0.001	10.2483
	97 -> 107	6.0048	206.48	0.0019	-10.6372
	97 -> 107	6.1388	201.97	0.001	3.3009
	97 -> 107	6.2134	199.54	0.0068	-10.6007
	105 -> 109	6.2915	197.07	0.1044	-61.8421
	98 -> 107	6.4548	192.08	0.0034	-3.3723
	97 -> 107	6.6671	185.96	0.0554	-3.6749
	101 -> 109	6.6759	185.72	0.3208	-34.5654
	104 -> 108	6.7359	184.07	0.046	41.8873
	91 -> 107	6.8091	182.09	0.0247	-2.1455
	97 -> 107	6.8117	182.02	0.0957	10.0024
	97 -> 107	6.9145	179.31	0.0761	6.0325
	90 -> 107	6.9737	177.79	0.1206	-127.4564
	100 -> 109	7.0248	176.5	0.024	19.9779
	99 -> 109	7.0841	175.02	0.0707	-56.7047
	97 -> 107	7.1073	174.45	0.0427	-17.9939
	90 -> 107	7.1267	173.97	0.0644	-22.5211
	99 -> 109	7.1759	172.78	0.05	127.1872
	100 -> 109	7.2286	171.52	0.2363	-19.7235
	100 -> 108	7.3392	168.93	0.0126	-9.1887
	102 -> 110	7.3704	168.22	0.0067	4.7305
	86 -> 107	7.3908	167.75	0.0096	-6.4449
	99 -> 109	7.4583	166.24	0.1351	101.6966

	86 -> 107	7.5458	164.31	0.0133	3.4142
	86 -> 107	7.5482	164.26	0.0965	82.4922
	86 -> 107	7.5836	163.49	0.0991	-7.7133
	99 -> 110	7.6178	162.76	0.048	-21.1836
	101 -> 108	7.7136	160.73	0.0286	20.6891
	98 -> 109	7.7517	159.94	0.0024	0.942
	101 -> 108	7.8042	158.87	0.0006	-3.7867
	86 -> 107	7.8198	158.55	0.008	-34.4234
	86 -> 107	7.8264	158.42	0.0303	-7.3201
	101 -> 110	7.8818	157.3	0.0012	-0.0077
	99 -> 109	7.9184	156.58	0.0401	47.9556
	101 -> 110	7.9406	156.14	0.0101	10.5189
	99 -> 109	8.0104	154.78	0.0274	-37.5008
	97 -> 108	8.0233	154.53	0.0469	34.4456
	99 -> 108	8.037	154.27	0.0245	13.5068
	99 -> 108	8.1312	152.48	0.0224	19.9703
	81 -> 107	8.1511	152.11	0.0076	-5.0236
	81 -> 107	8.1595	151.95	0.0055	11.336
	95 -> 109	8.2073	151.07	0.0136	33.6118
	100 -> 109	8.2177	150.87	0.0042	1.2194
	99 -> 109	8.2461	150.36	0.0063	22.8511

[a]Excitation energy. [b]Wavelength. [c]Oscillator strength. [d]Rotatory strength in velocity form (10^{-40}cgs.).

Quantum-chemical computation for ^{13}C NMR

To verify the structure of **2**, the quantum chemical prediction on the ^{13}C NMR shifts of 2*S**,5*R**,8*R**,10*R**-**2** was executed with scaling methods [1,2]. Conformational analyses of **2** were performed using the same method as the calculated ECD, the above mentioned. Conformations were optimized with M062X function applying 6-31G(d) basis set in the gas phase. NMR calculations were carried out with mPW1PW91 function applying 6-31G(d) basis set in chloroform solvent with SMD solvation model.

1. Li, J.; Liu, J. K.; Wang, W. X. GIAO ^{13}C NMR Calculation with Sorted Training Sets Improves Accuracy and Reliability for Structural Assignation. *J. Org. Chem.* **2020**, *85*, 11350–11358.
2. Lodewyk, M. W.; Siebert, M. R.; Tantillo, D. J. Computational prediction of ^{1}H and ^{13}C chemical shifts: a useful tool for natural product, mechanistic, and synthetic organic chemistry. *Chem. Rev.* **2012**, *112*, 1839–1862.

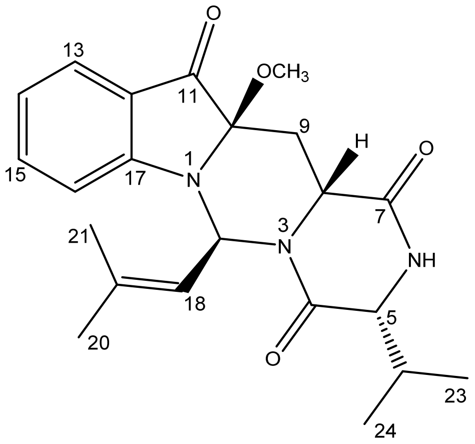

2*S**,5*R**,8*R**,10*R**-**2**

Optimized coordinates (Å) of **2** in gas phase at M062X/6-31G(d) level.

C	-2.701627	-3.318374	-1.617503
C	-1.881821	-2.587171	-2.488203
C	-1.3895	-1.324344	-2.170649
C	-1.746343	-0.781685	-0.932264
C	-2.531205	-1.532856	-0.0427
C	-3.027875	-2.7887	-0.376771
N	-1.43321	0.476068	-0.404756
C	-1.649774	0.412232	1.059137
C	-2.64107	-0.77296	1.206049
C	-0.255447	1.185806	-0.876529
N	0.939061	0.664122	-0.192849
C	0.865896	0.780334	1.259411
C	-0.355399	0.011097	1.784957
O	-3.298066	-0.988938	2.197294
O	-2.089709	1.609755	1.605789
C	1.830333	-0.079932	-0.892833
C	2.980976	-0.727212	-0.13643
N	3.053095	-0.326916	1.256782
C	2.106097	0.305565	1.994573
C	-0.389948	2.682844	-0.69478
O	1.756704	-0.253567	-2.103596
O	2.211315	0.493551	3.191913
C	0.445066	3.570629	-1.241811
C	0.262305	5.046498	-1.011753
C	1.628329	3.197632	-2.095276
C	2.894146	-2.259911	-0.32353
C	4.10595	-2.950826	0.299611
C	1.589441	-2.825937	0.234156
H	0.751566	1.843535	1.50333
H	3.888579	-0.378021	-0.645086
C	-3.3718	2.023642	1.155824

H	-3.059828	-4.299369	-1.909583
H	-1.611622	-3.020332	-3.446739
H	-0.727021	-0.807679	-2.855898
H	-3.63611	-3.331763	0.340316
H	-0.120745	0.949378	-1.931486
H	-0.483061	0.190254	2.854464
H	-0.181679	-1.059683	1.628169
H	3.858446	-0.627038	1.794173
H	-1.208629	3.025895	-0.071455
H	-0.587291	5.253759	-0.356892
H	1.161834	5.480502	-0.55912
H	0.10407	5.567815	-1.963201
H	1.631273	2.151938	-2.411619
H	1.662176	3.829722	-2.989841
H	2.559171	3.383354	-1.54413
H	2.912358	-2.412154	-1.408229
H	4.087045	-2.874638	1.393186
H	5.047128	-2.521818	-0.061799
H	4.106311	-4.016152	0.052344
H	1.548998	-3.909055	0.086067
H	0.711855	-2.393806	-0.262589
H	1.514922	-2.637395	1.311961
H	-3.524569	3.028525	1.551179
H	-4.150474	1.358039	1.540717
H	-3.409648	2.048634	0.060202

Figure S4. HRESIMS of compound **1**

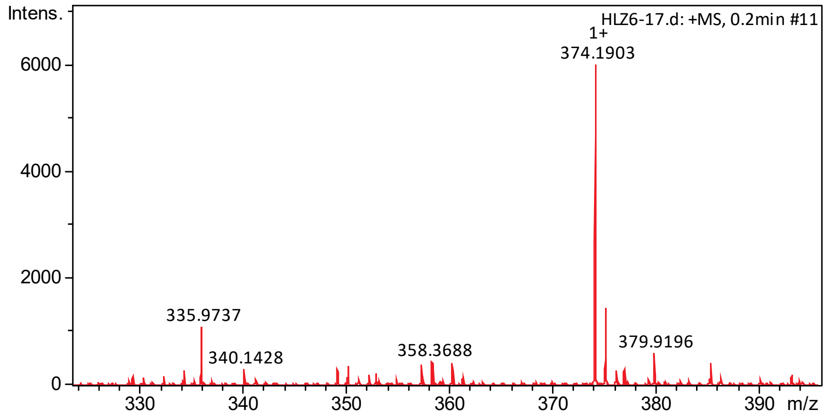

Figure S5. UV of compound **1** (in MeOH)

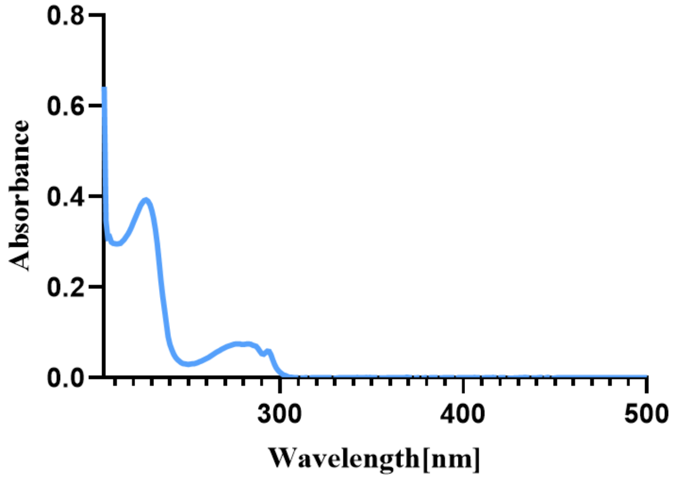

Figure S6. IR of compound **1** (KBr disc)

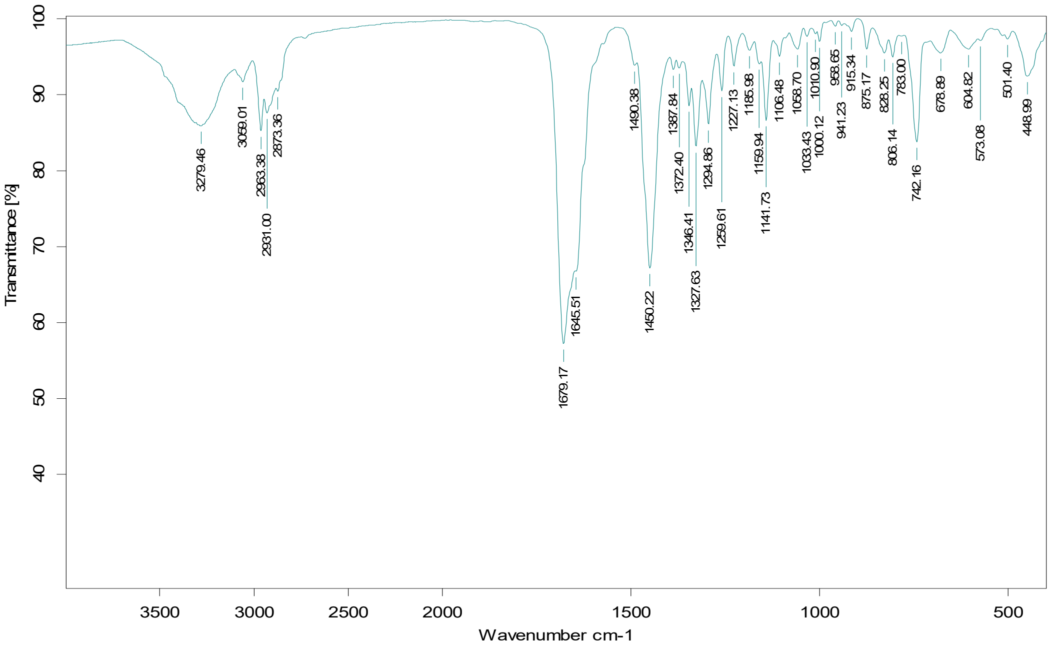

Figure S7. ^{1}H NMR spectrum of compound **1** (in $CDCl_3$)

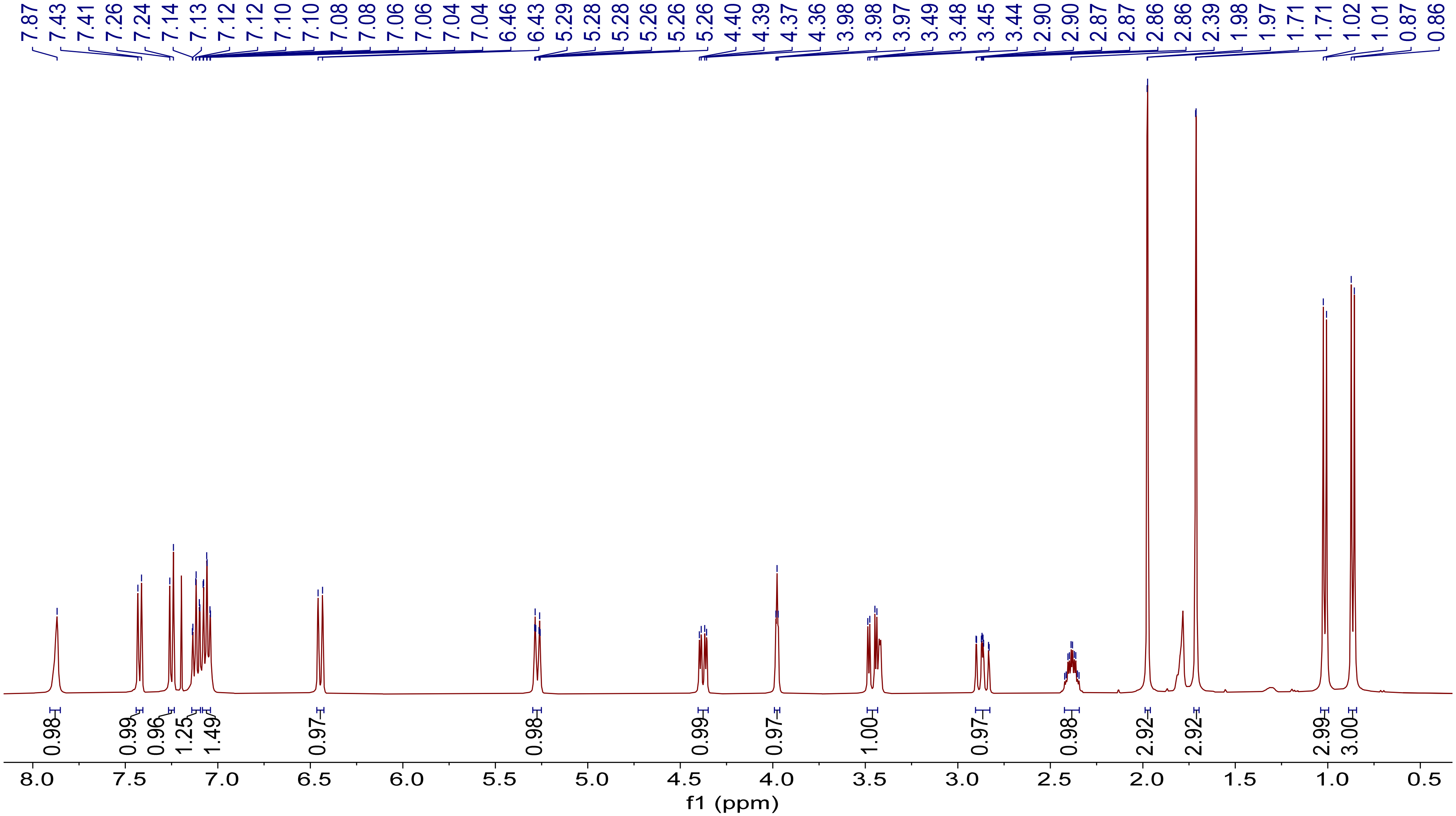

Figure S8. ^{13}C NMR spectra of compound **1** (in $CDCl_3$)

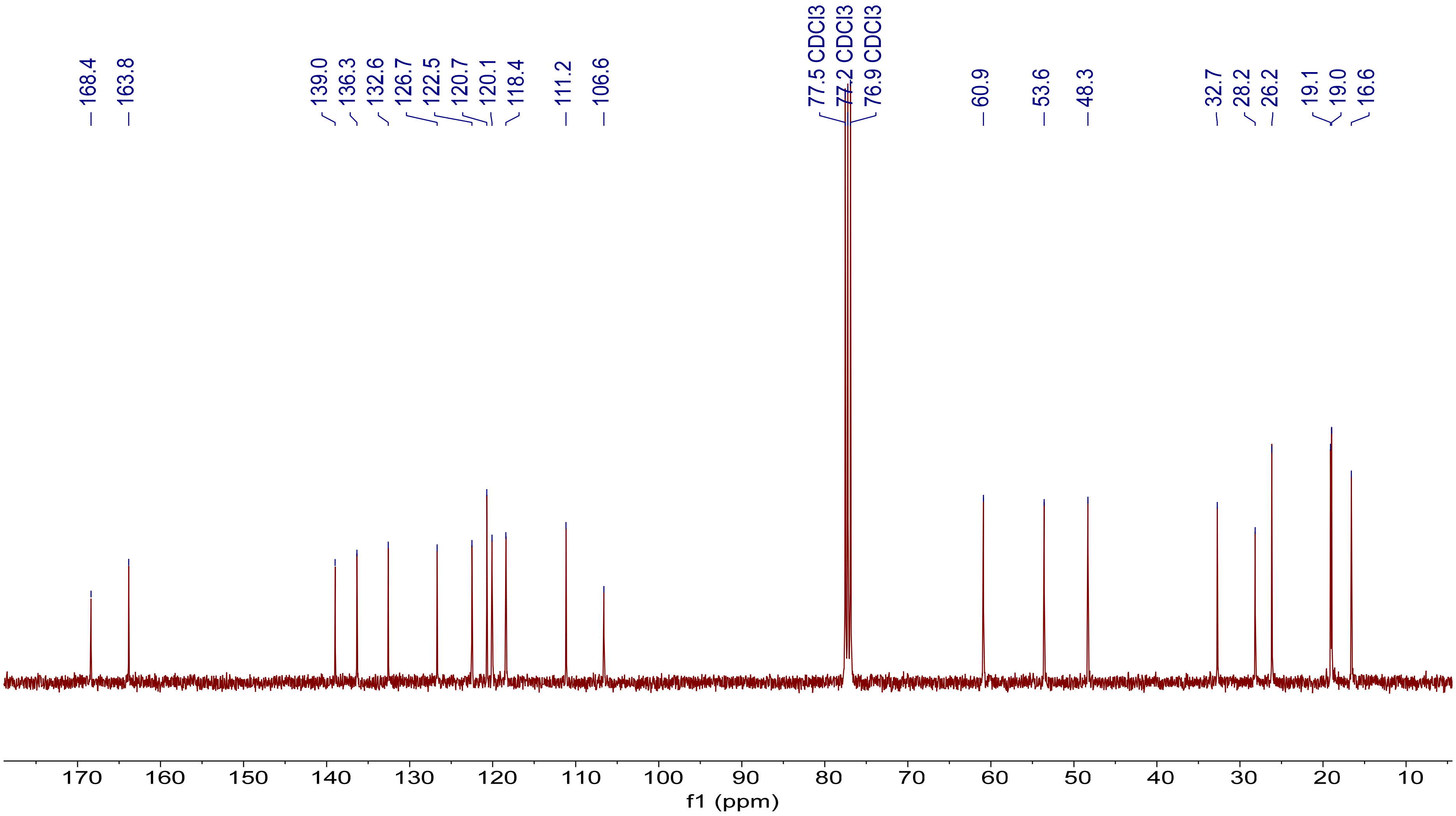

Figure S9. DEPT135 spectrum of compound **1** (in $CDCl_3$)

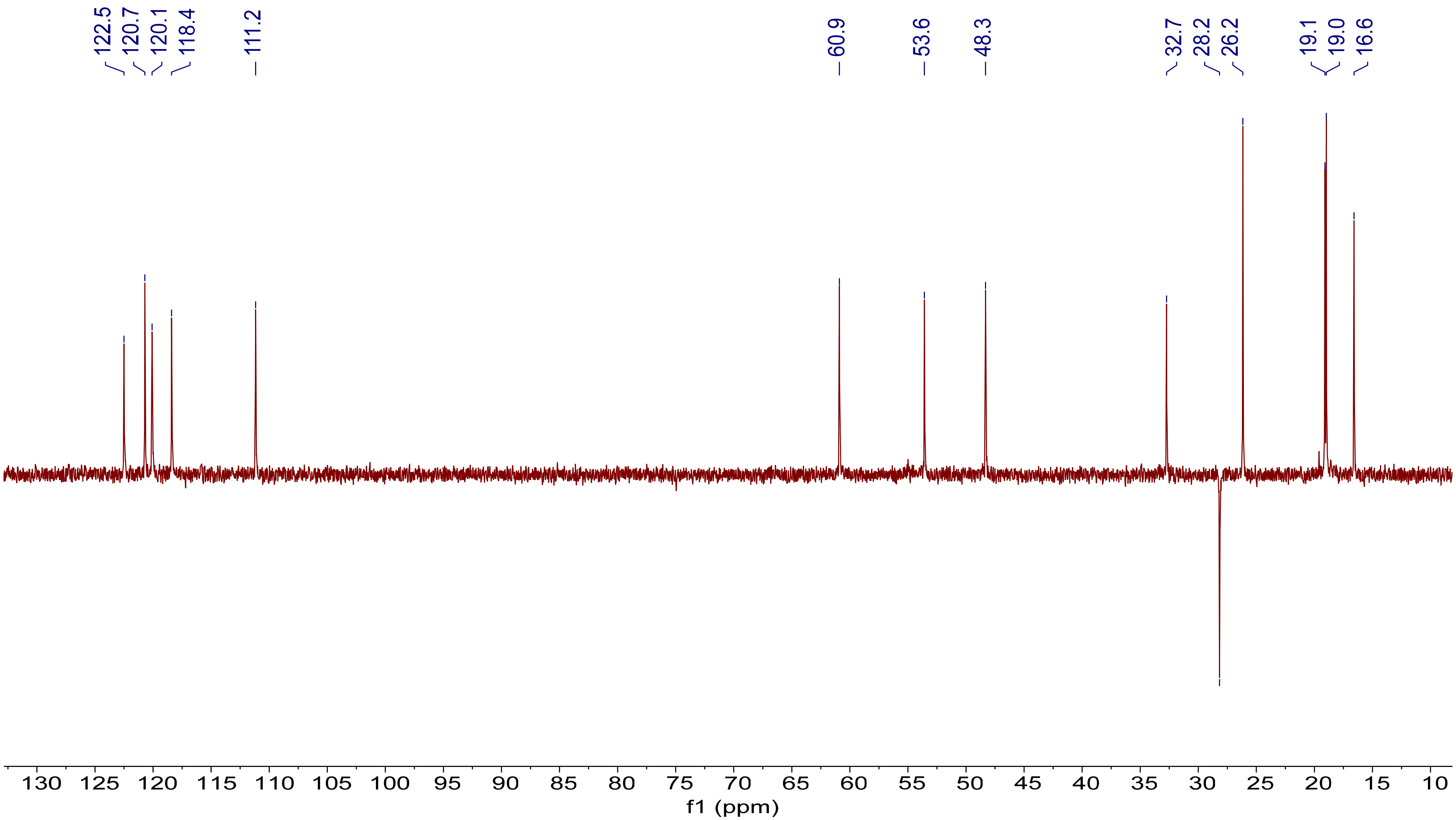

Figure S10. HSQC spectrum of compound **1** (in $CDCl_3$)

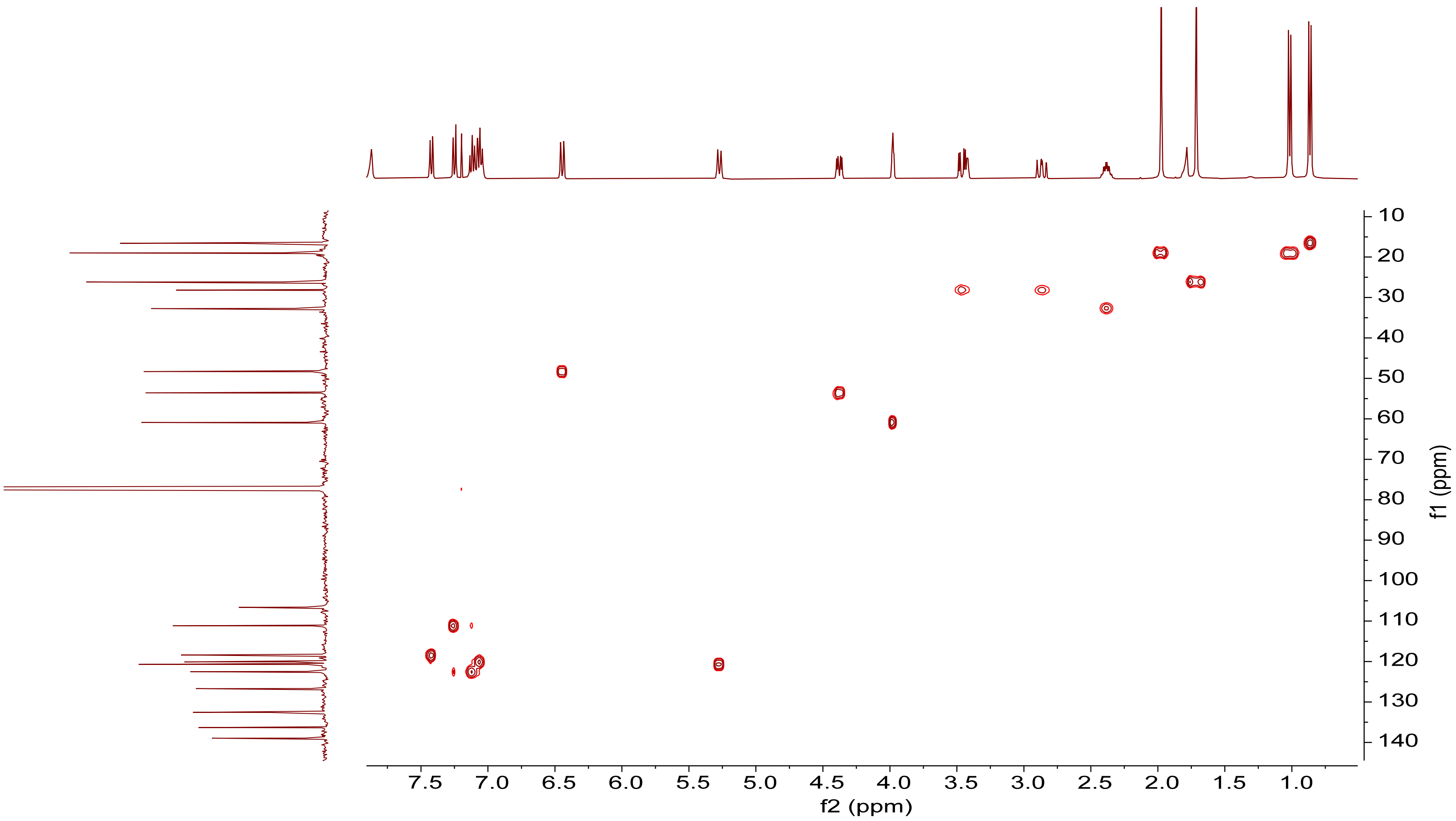

Figure S11. HMBC spectrum of compound **1** (in $CDCl_3$)

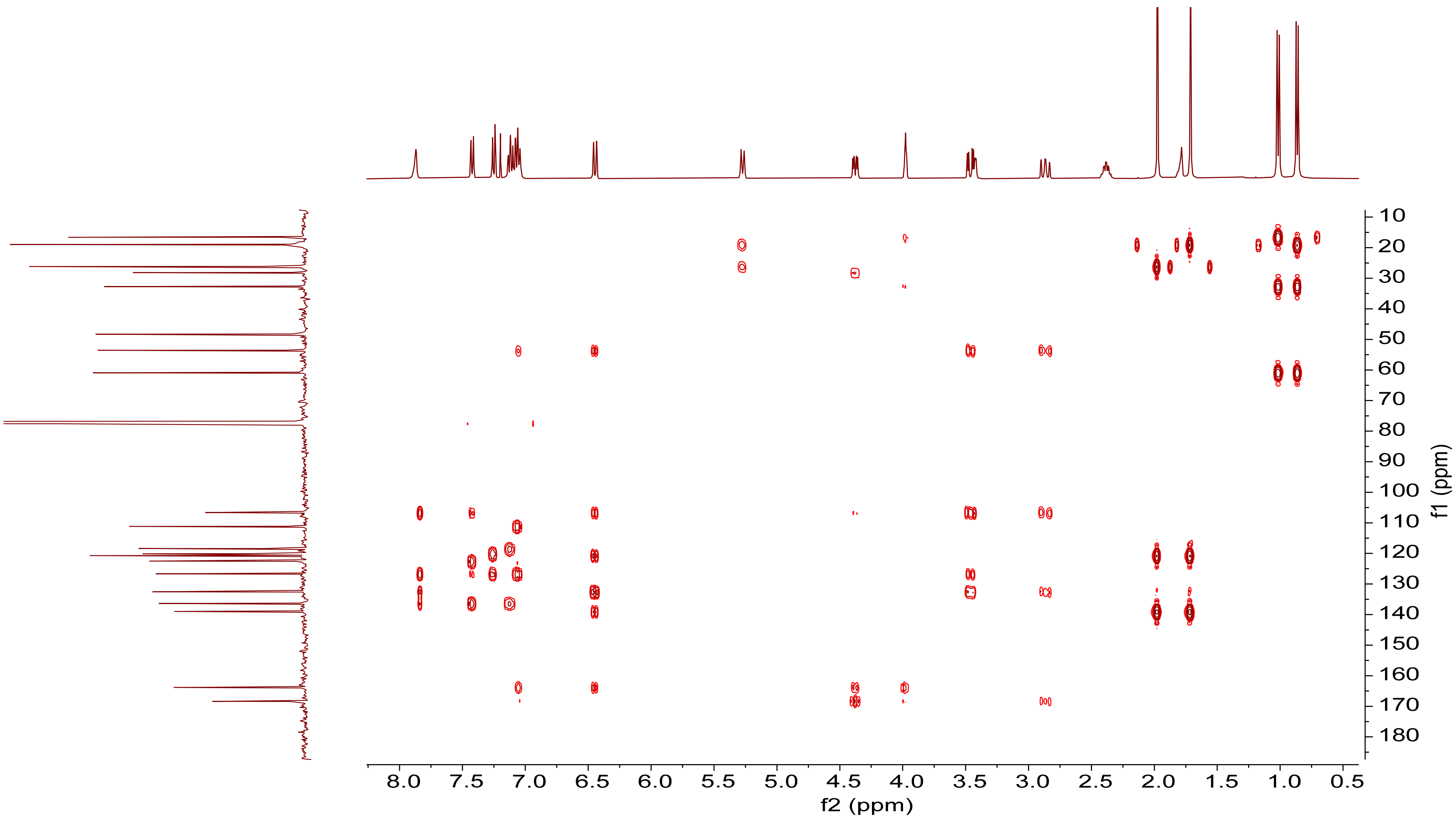

Figure S12. ^1H–^1H COSY spectrum of compound **1** (in $CDCl_3$)

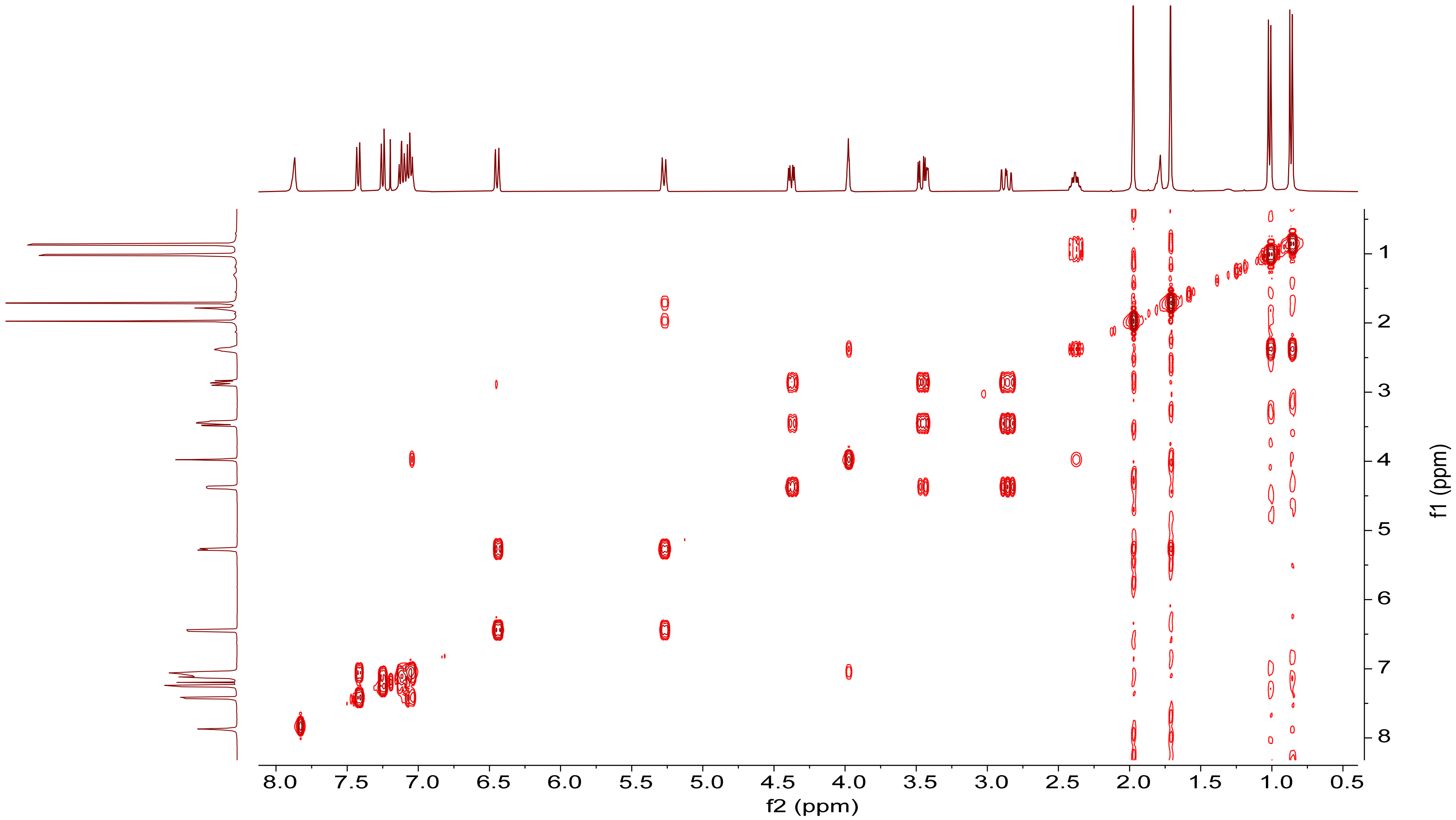

Figure S13. NOESY spectrum of compound **1** (in $CDCl_3$)

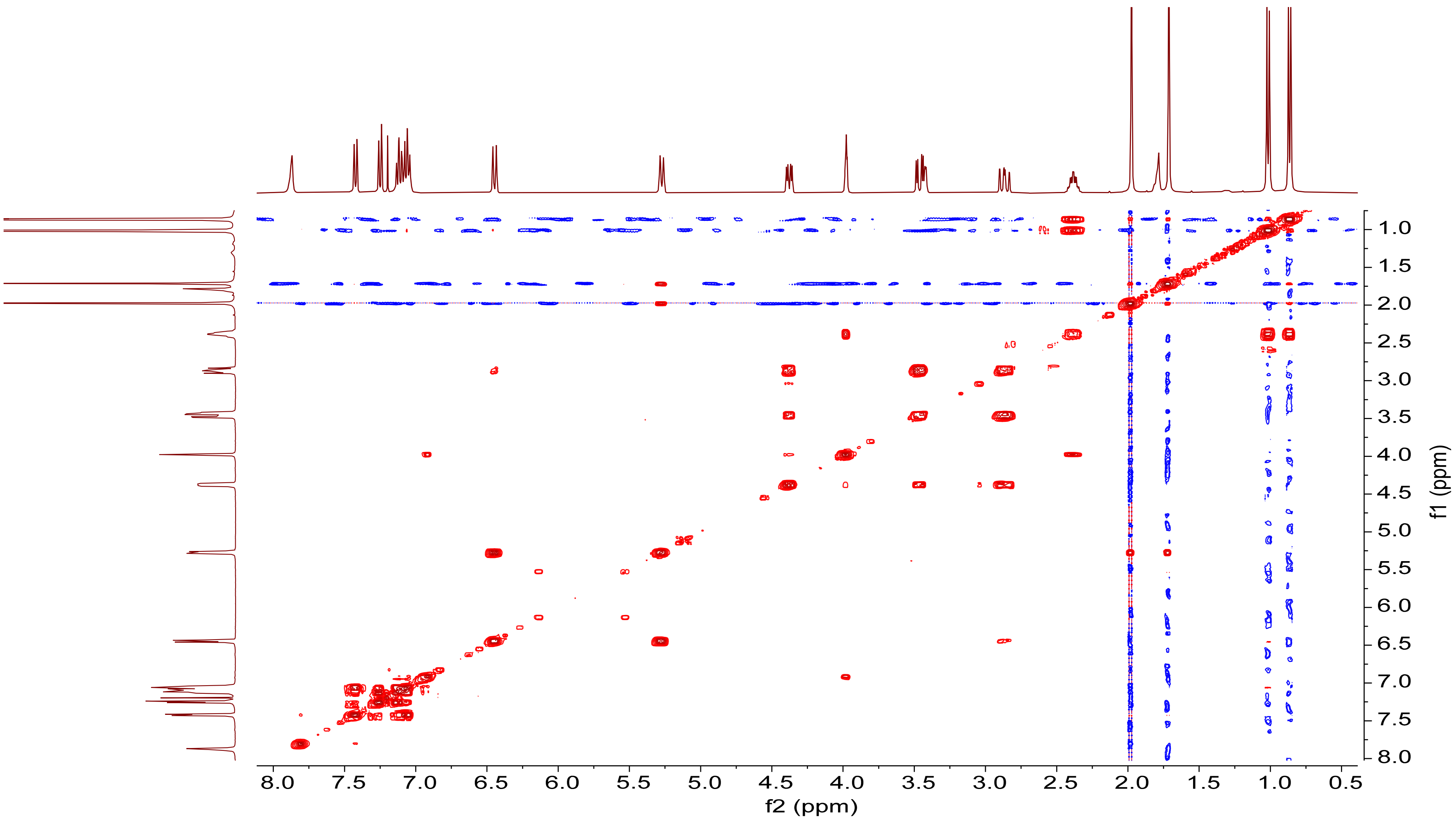

Figure S14. HRESIMS of compound **2**

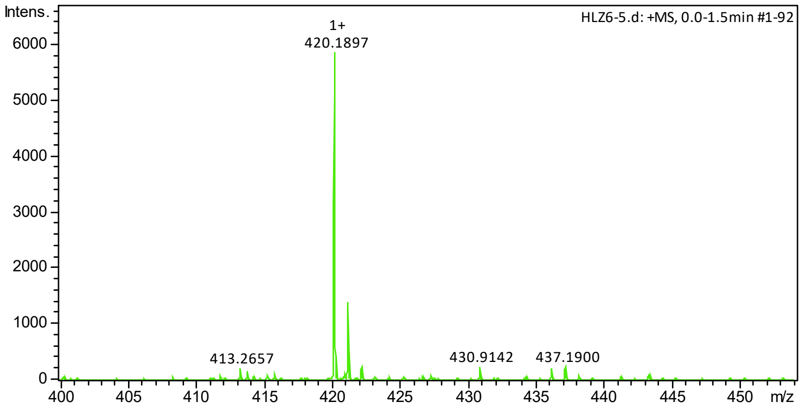

Figure S15. UV of compound **2** (in MeOH)

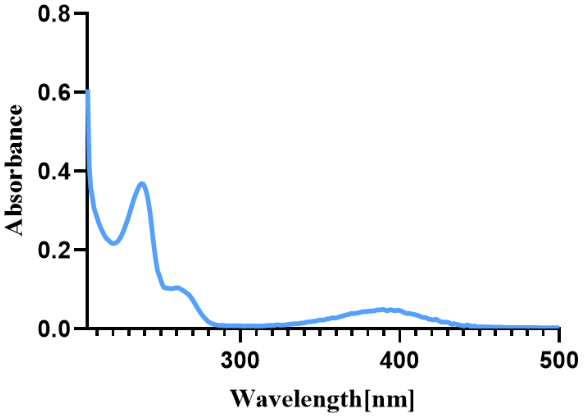

Figure S16. IR of compound **2** (KBr disc)

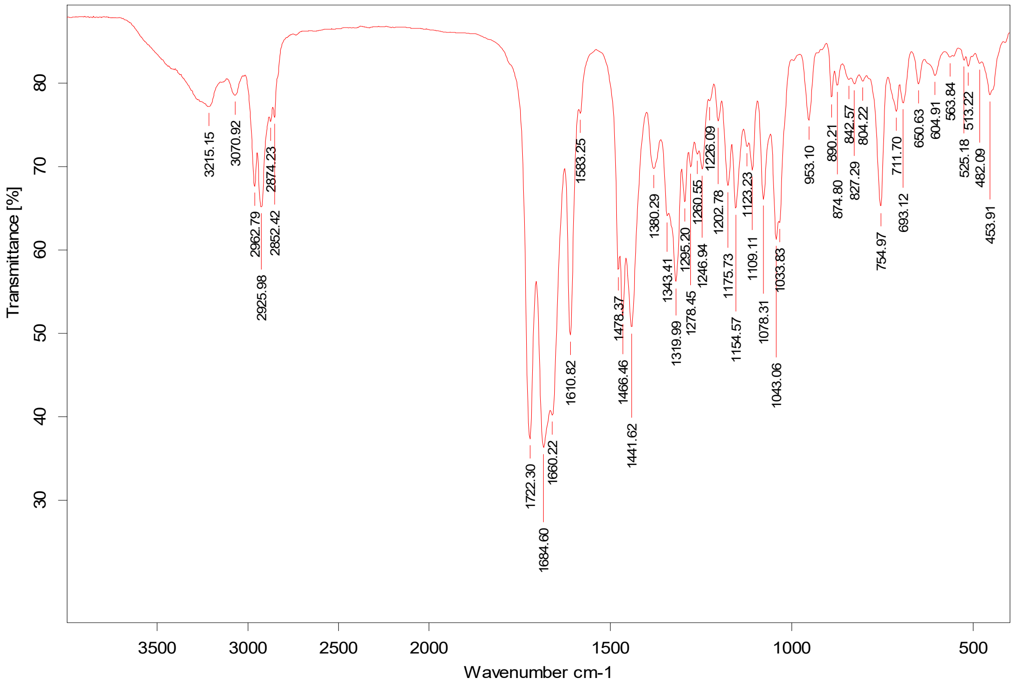

Figure S17. ^{1}H NMR spectrum of compound **2** (in $CDCl_3$)

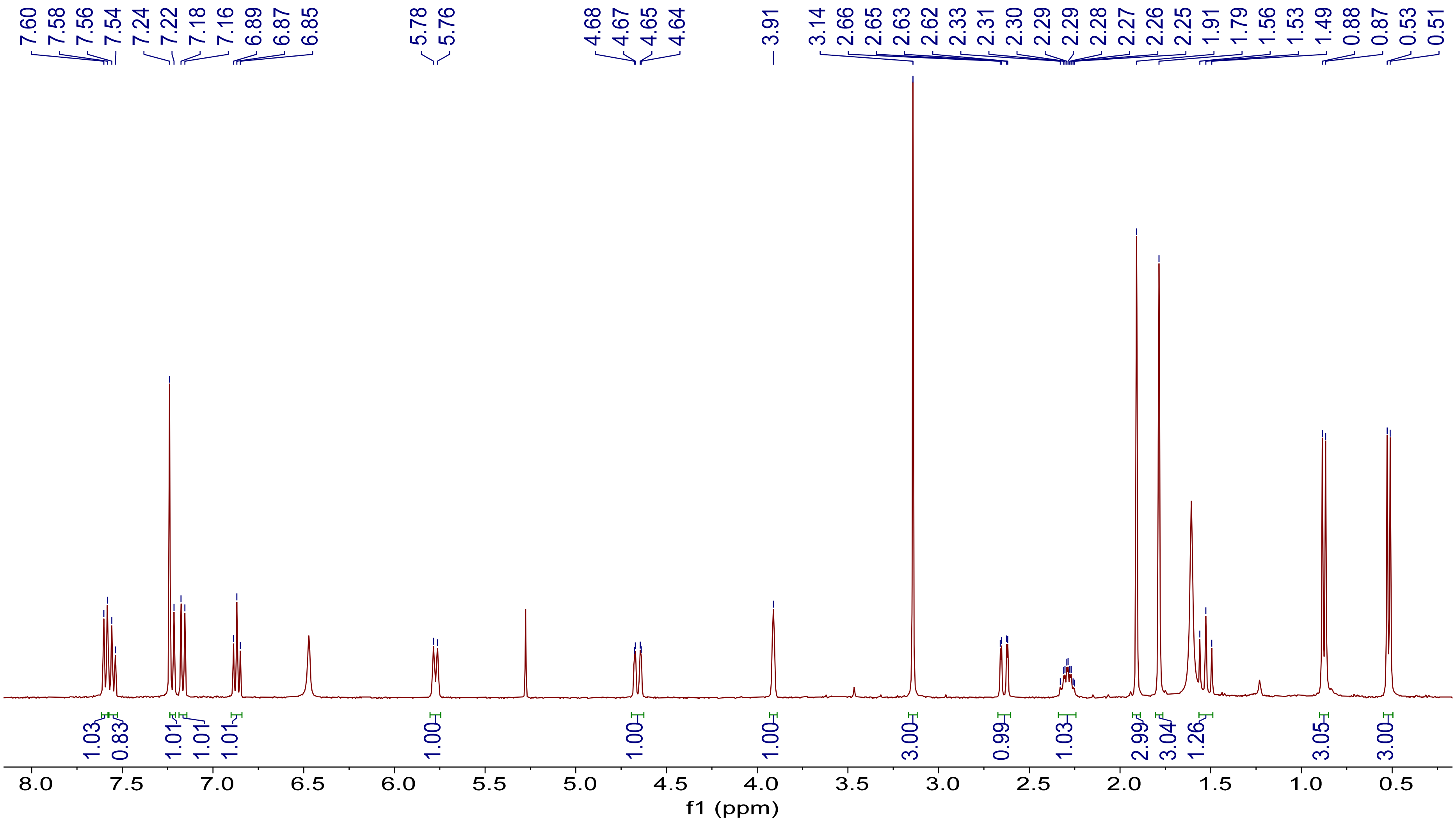

Figure S18. ^{13}C NMR spectrum of compound **2** (in $CDCl_3$)

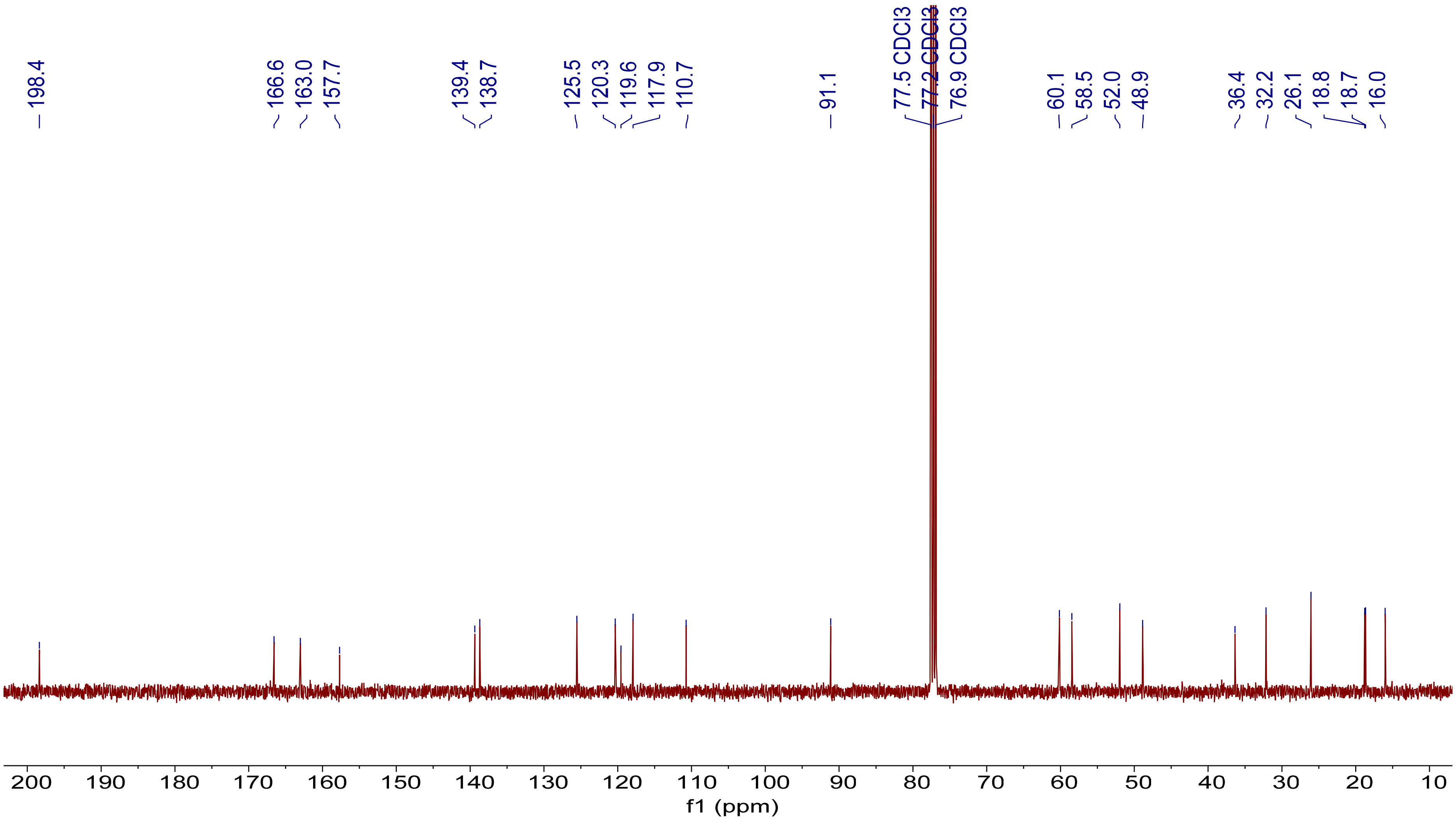

Figure S19. DEPT135 spectrum of compound **2** (in $CDCl_3$)

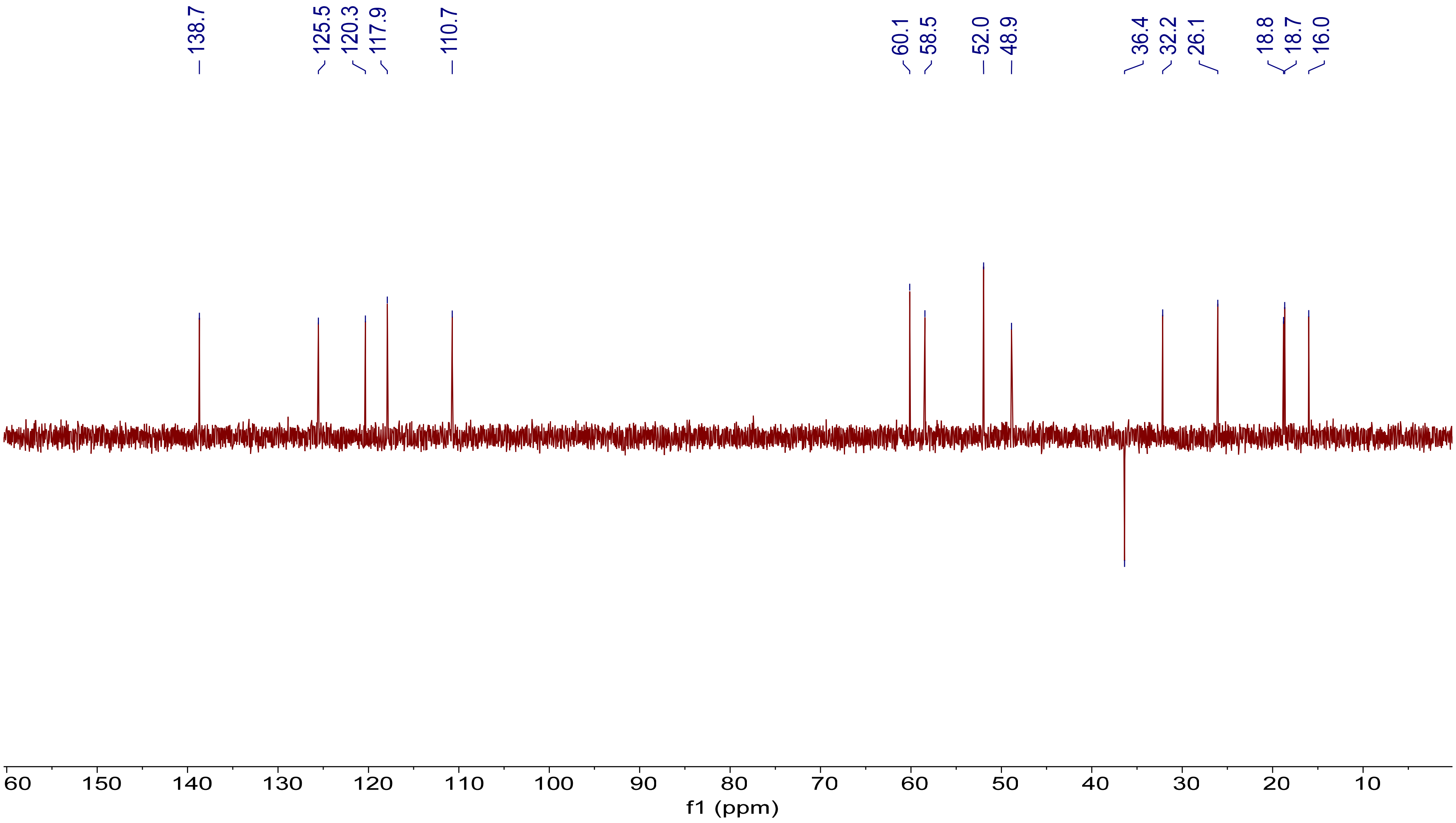

Figure S20. HSQC spectrum of compound **2** (in $CDCl_3$)

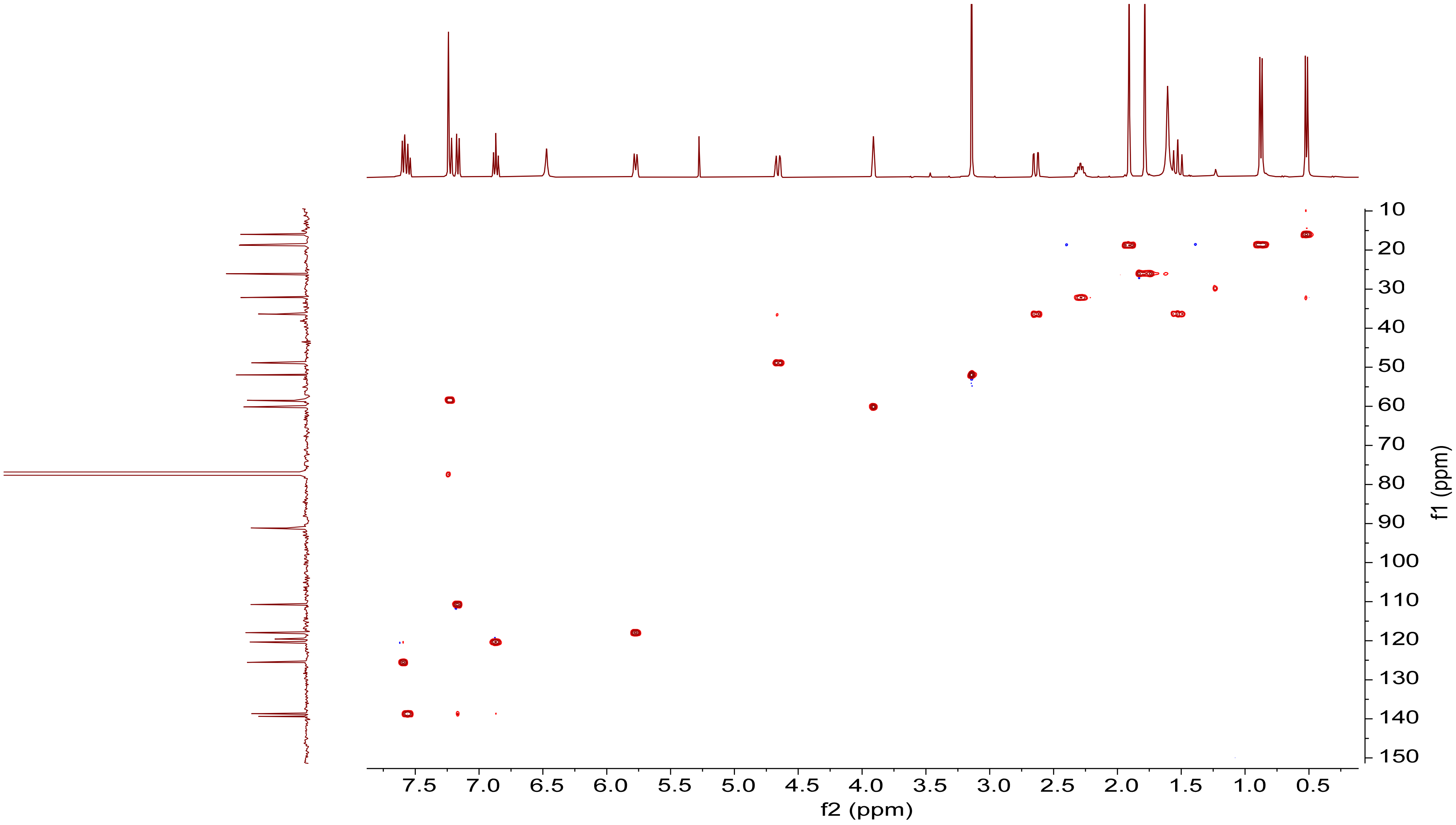

Figure S21. HMBC spectrum of compound **2** (in $CDCl_3$)

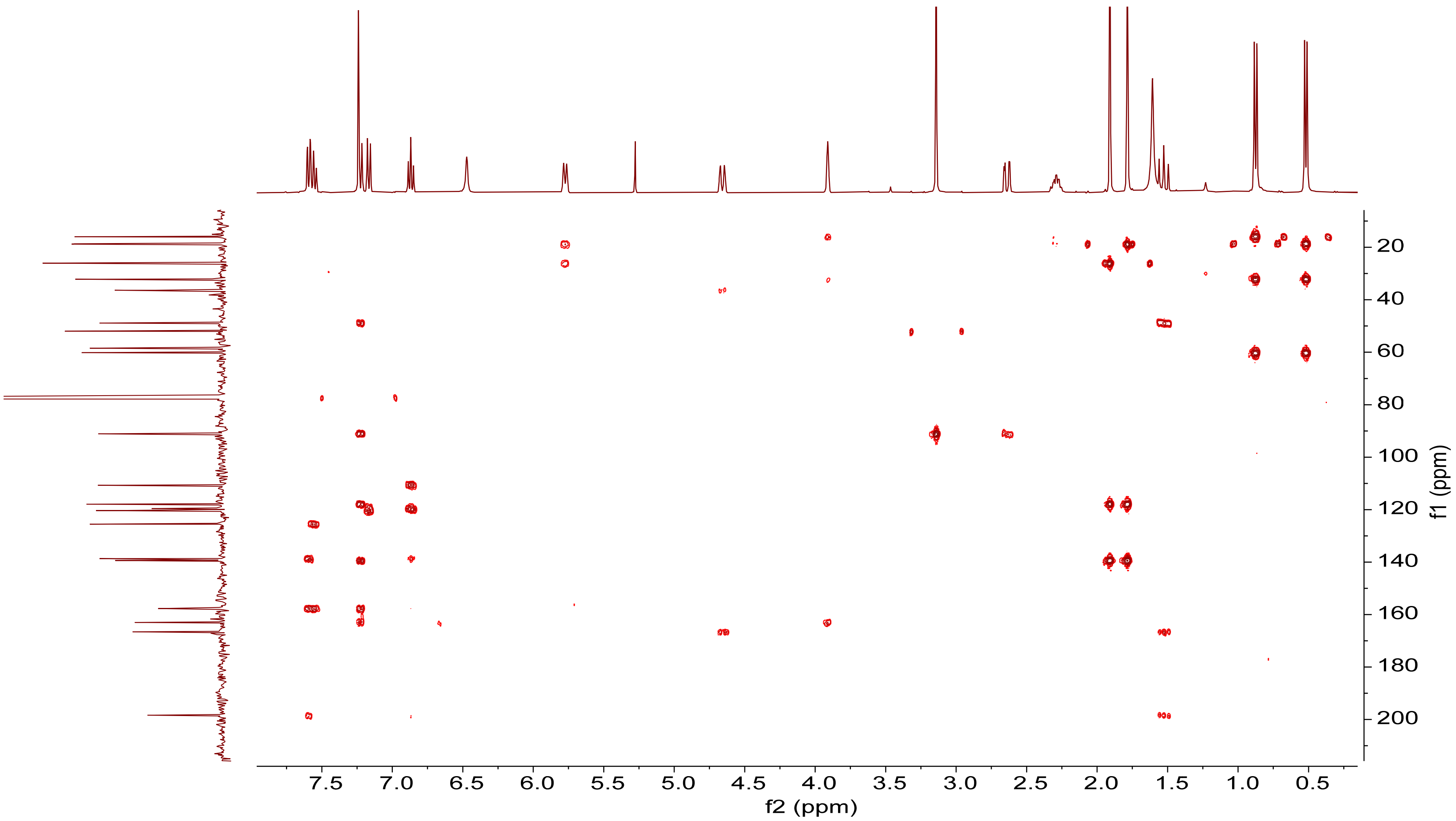

Figure S22. ^1H–^1H COSY spectrum of compound **2** (in $CDCl_3$)

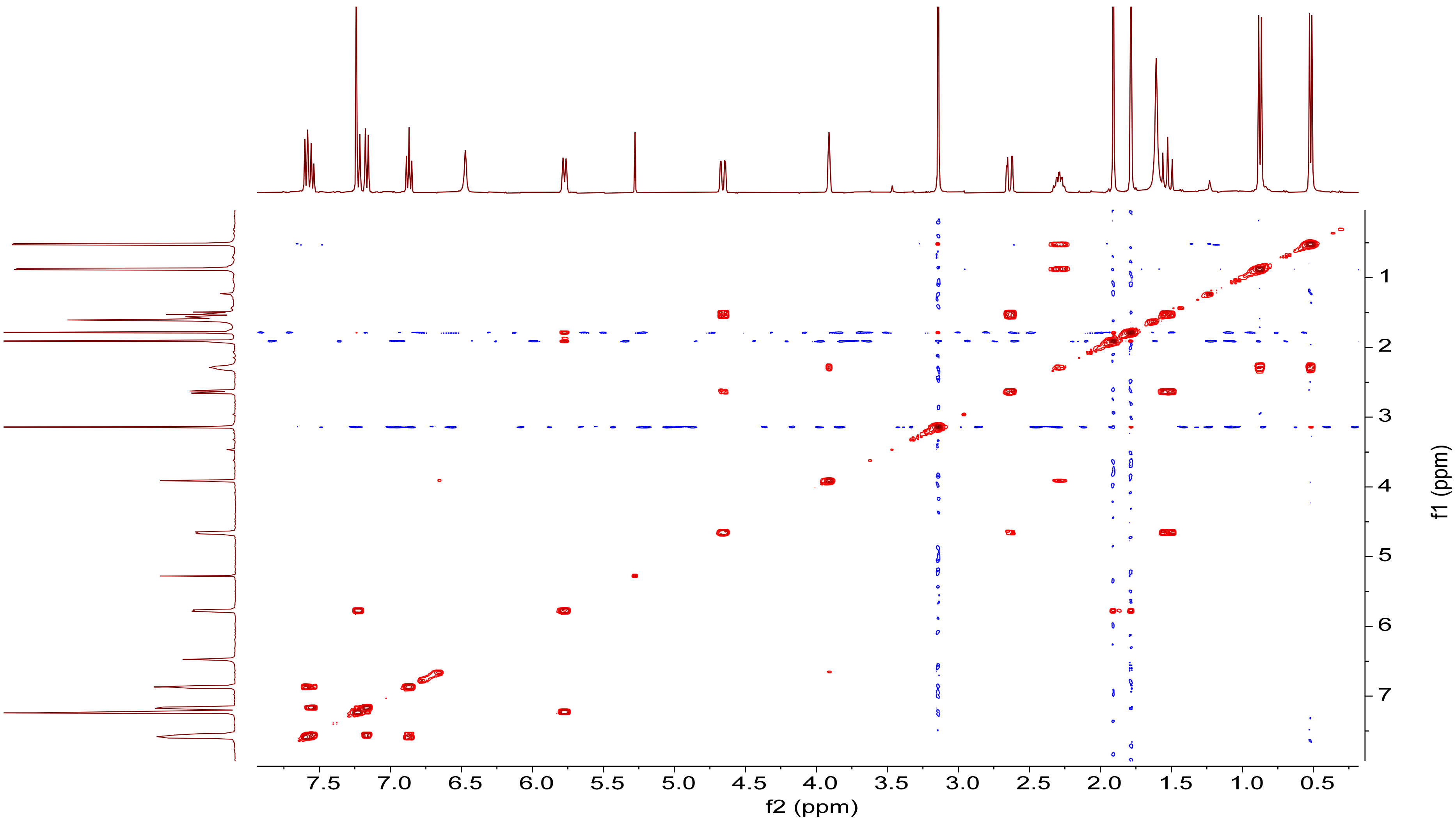

Figure S23. NOESY spectrum of compound **2** (in $CDCl_3$)

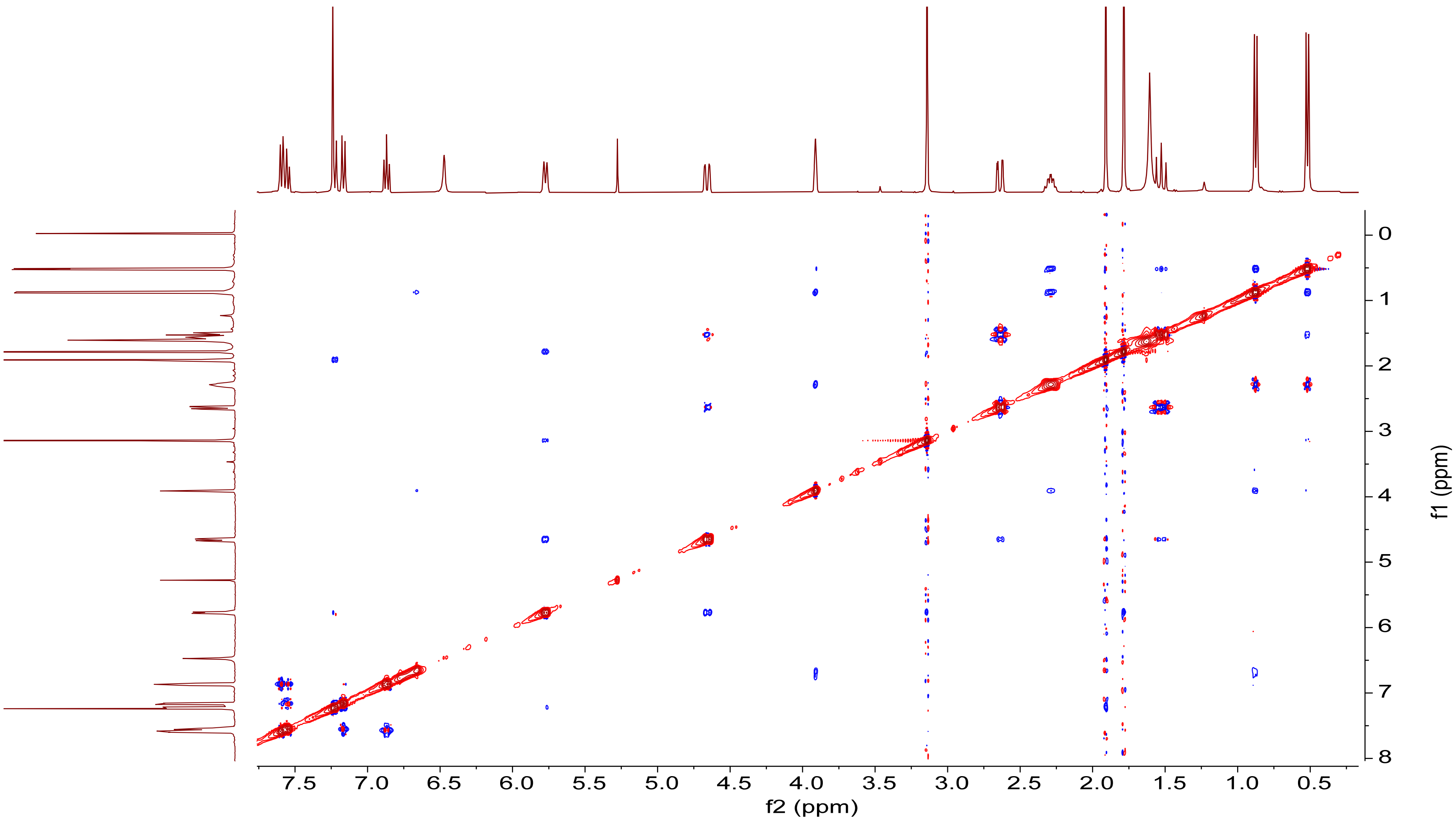

Supplement: Supplementary file 1 [file marinedrugs-20-00191-s001.zip › marinedrugs-1608098-supplementary.pdf]
